# Supplementary material for: Population density and spreading of COVID-19 in England and Wales
Source: PLoS One. 2022 Mar 31;17(3):e0261725. doi: 10.1371/journal.pone.0261725 (PMC8970409; doi:10.1371/journal.pone.0261725)
Supplement: S1 Fig — Black line represents the generalised logistic distribution and the red dashed line represents the normal distribution. (PDF) [file pone.0261725.s001.pdf]

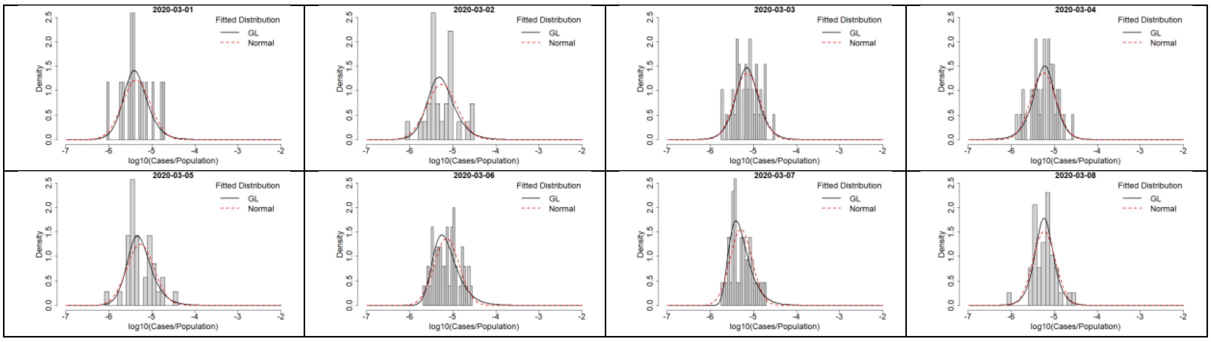
2020-03-01
2020-03-02
2020-03-03
2020-03-04
2020-03-05
2020-03-06
2020-03-07
2020-03-08
Fitted Distribution
GL
Normal
Density
log10(Cases/Population)

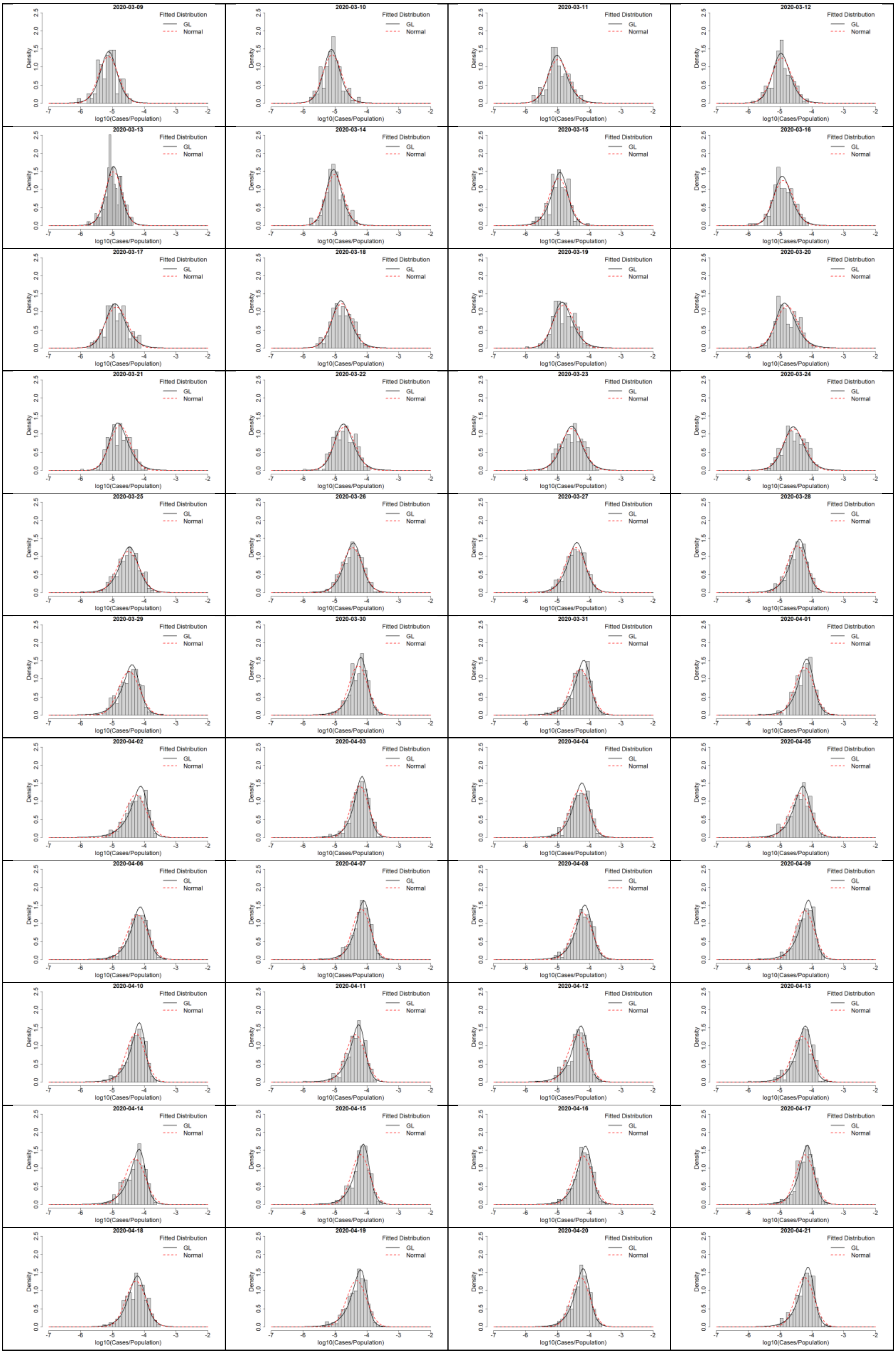

2020-03-09
2020-03-10
2020-03-11
2020-03-12
2020-03-13
2020-03-14
2020-03-15
2020-03-16
2020-03-17
2020-03-18
2020-03-19
2020-03-20
2020-03-21
2020-03-22
2020-03-23
2020-03-24
2020-03-25
2020-03-26
2020-03-27
2020-03-28
2020-03-29
2020-03-30
2020-03-31
2020-04-01
2020-04-02
2020-04-03
2020-04-04
2020-04-05
2020-04-06
2020-04-07
2020-04-08
2020-04-09
2020-04-10
2020-04-11
2020-04-12
2020-04-13
2020-04-14
2020-04-15
2020-04-16
2020-04-17
2020-04-18
2020-04-19
2020-04-20
2020-04-21
Fitted Distribution
GL
Normal
Density
log10(Cases/Population)

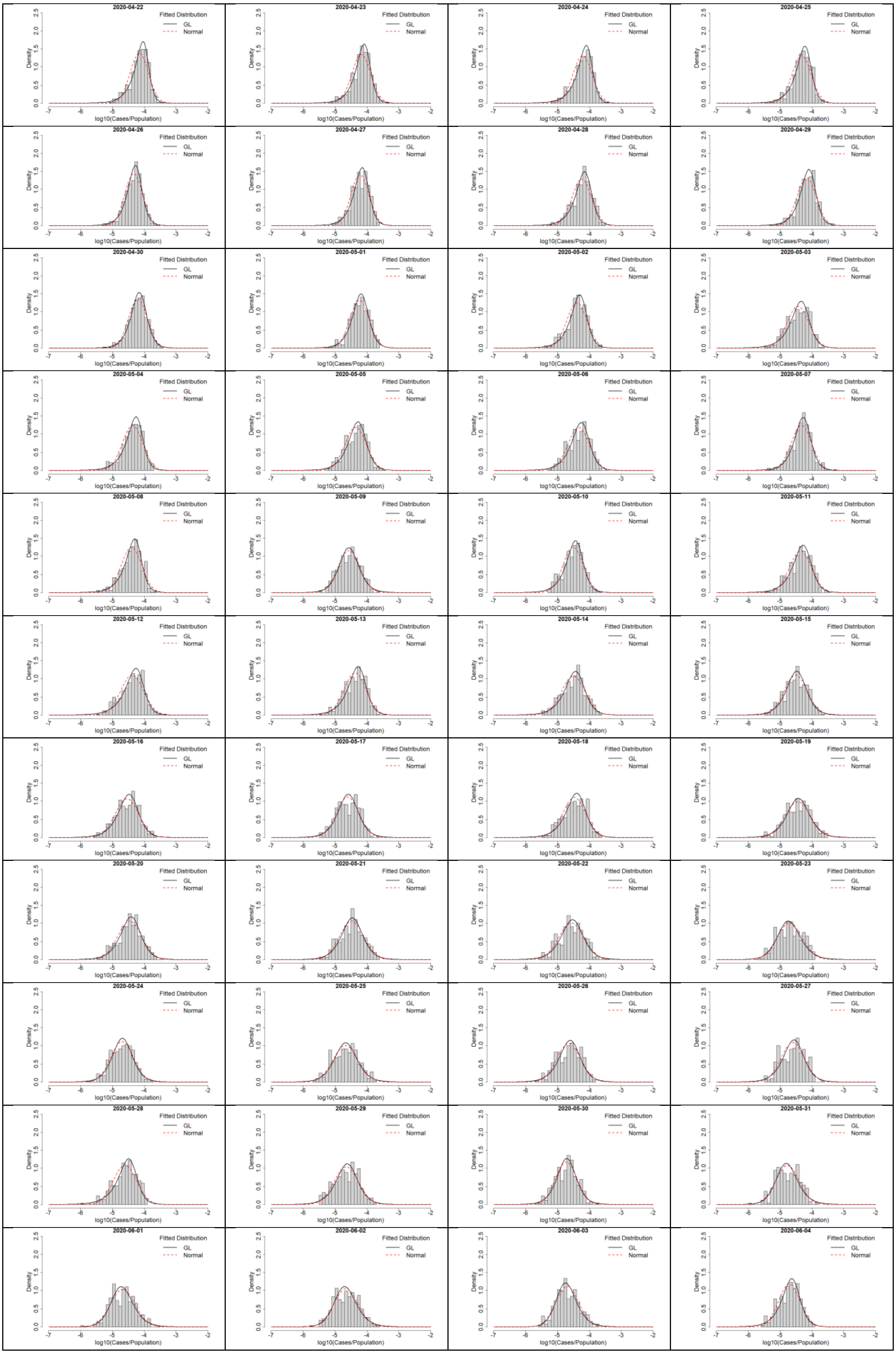
2020-04-22
2020-04-23
2020-04-24
2020-04-25
2020-04-26
2020-04-27
2020-04-28
2020-04-29
2020-04-30
2020-05-01
2020-05-02
2020-05-03
2020-05-04
2020-05-05
2020-05-06
2020-05-07
2020-05-08
2020-05-09
2020-05-10
2020-05-11
2020-05-12
2020-05-13
2020-05-14
2020-05-15
2020-05-16
2020-05-17
2020-05-18
2020-05-19
2020-05-20
2020-05-21
2020-05-22
2020-05-23
2020-05-24
2020-05-25
2020-05-26
2020-05-27
2020-05-28
2020-05-29
2020-05-30
2020-05-31
2020-06-01
2020-06-02
2020-06-03
2020-06-04
Fitted Distribution
GL
Normal
Density
log10(Cases/Population)

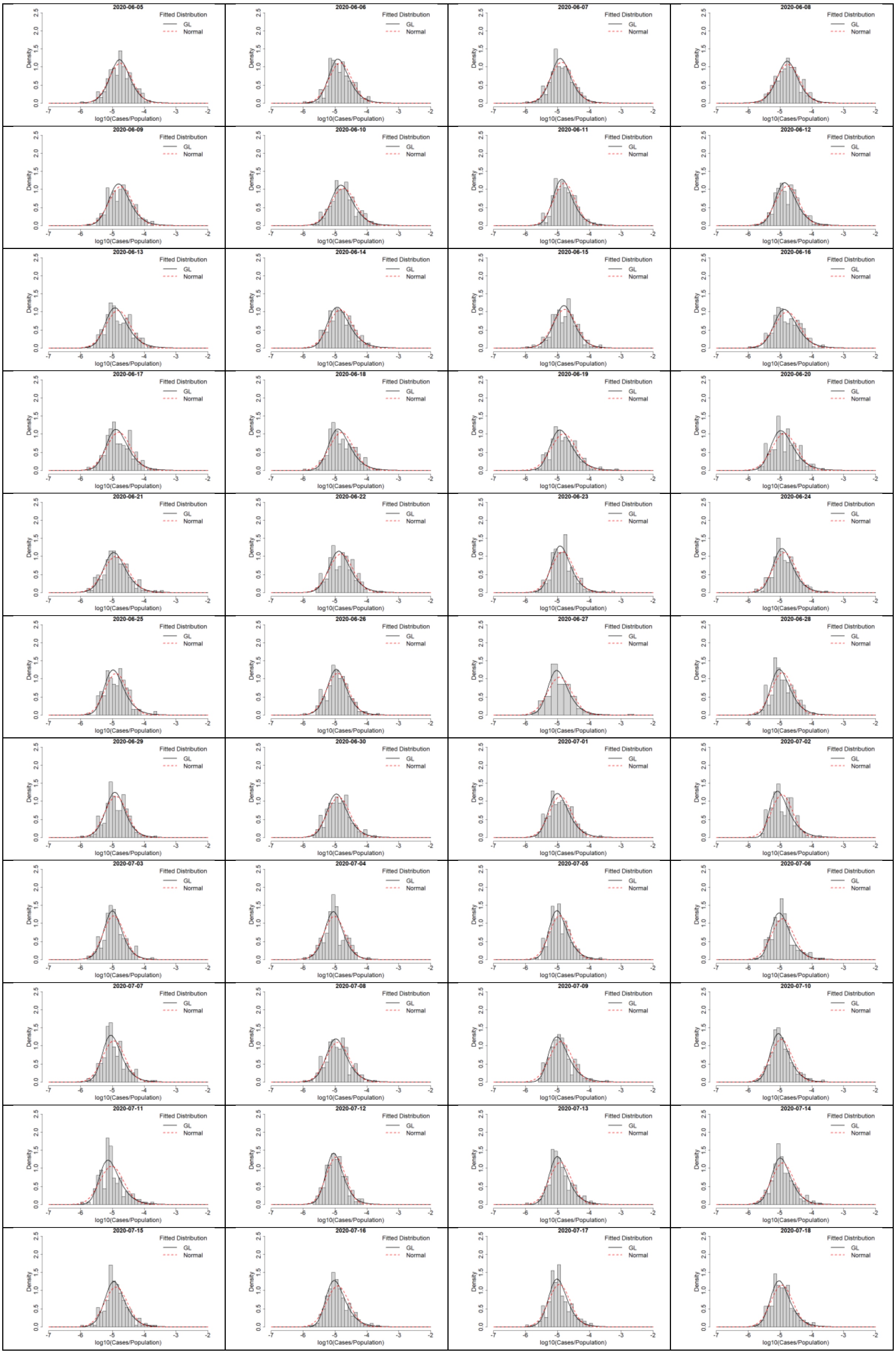
2020-06-05
2020-06-06
2020-06-07
2020-06-08
2020-06-09
2020-06-10
2020-06-11
2020-06-12
2020-06-13
2020-06-14
2020-06-15
2020-06-16
2020-06-17
2020-06-18
2020-06-19
2020-06-20
2020-06-21
2020-06-22
2020-06-23
2020-06-24
2020-06-25
2020-06-26
2020-06-27
2020-06-28
2020-06-29
2020-06-30
2020-07-01
2020-07-02
2020-07-03
2020-07-04
2020-07-05
2020-07-06
2020-07-07
2020-07-08
2020-07-09
2020-07-10
2020-07-11
2020-07-12
2020-07-13
2020-07-14
2020-07-15
2020-07-16
2020-07-17
2020-07-18
Fitted Distribution
GL
Normal
Density
log10(Cases/Population)

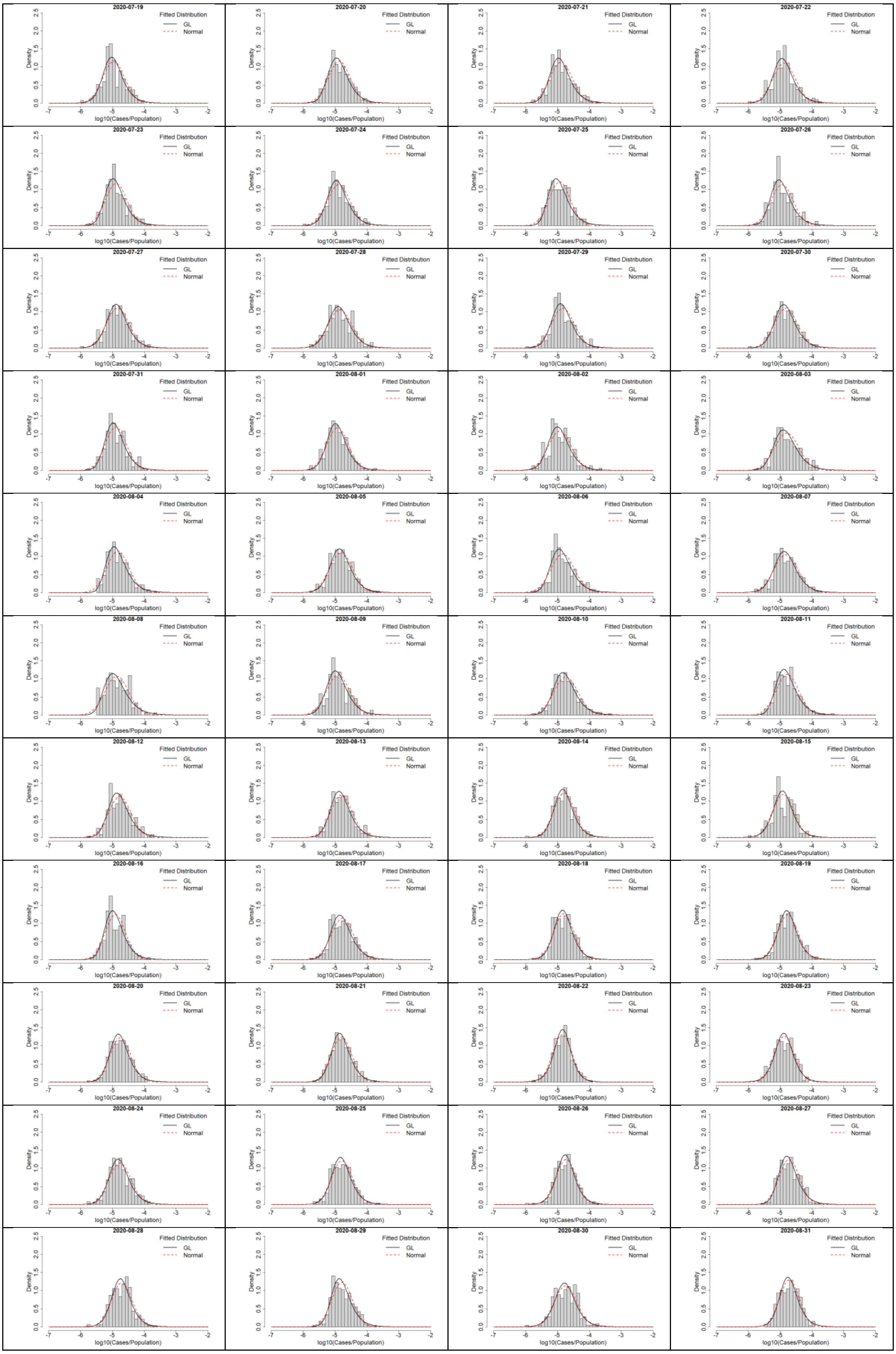
2020-07-19
2020-07-20
2020-07-21
2020-07-22
2020-07-23
2020-07-24
2020-07-25
2020-07-26
2020-07-27
2020-07-28
2020-07-29
2020-07-30
2020-07-31
2020-08-01
2020-08-02
2020-08-03
2020-08-04
2020-08-05
2020-08-06
2020-08-07
2020-08-08
2020-08-09
2020-08-10
2020-08-11
2020-08-12
2020-08-13
2020-08-14
2020-08-15
2020-08-16
2020-08-17
2020-08-18
2020-08-19
2020-08-20
2020-08-21
2020-08-22
2020-08-23
2020-08-24
2020-08-25
2020-08-26
2020-08-27
2020-08-28
2020-08-29
2020-08-30
2020-08-31
Fitted Distribution
GL
Normal
Density
log10(Cases/Population)

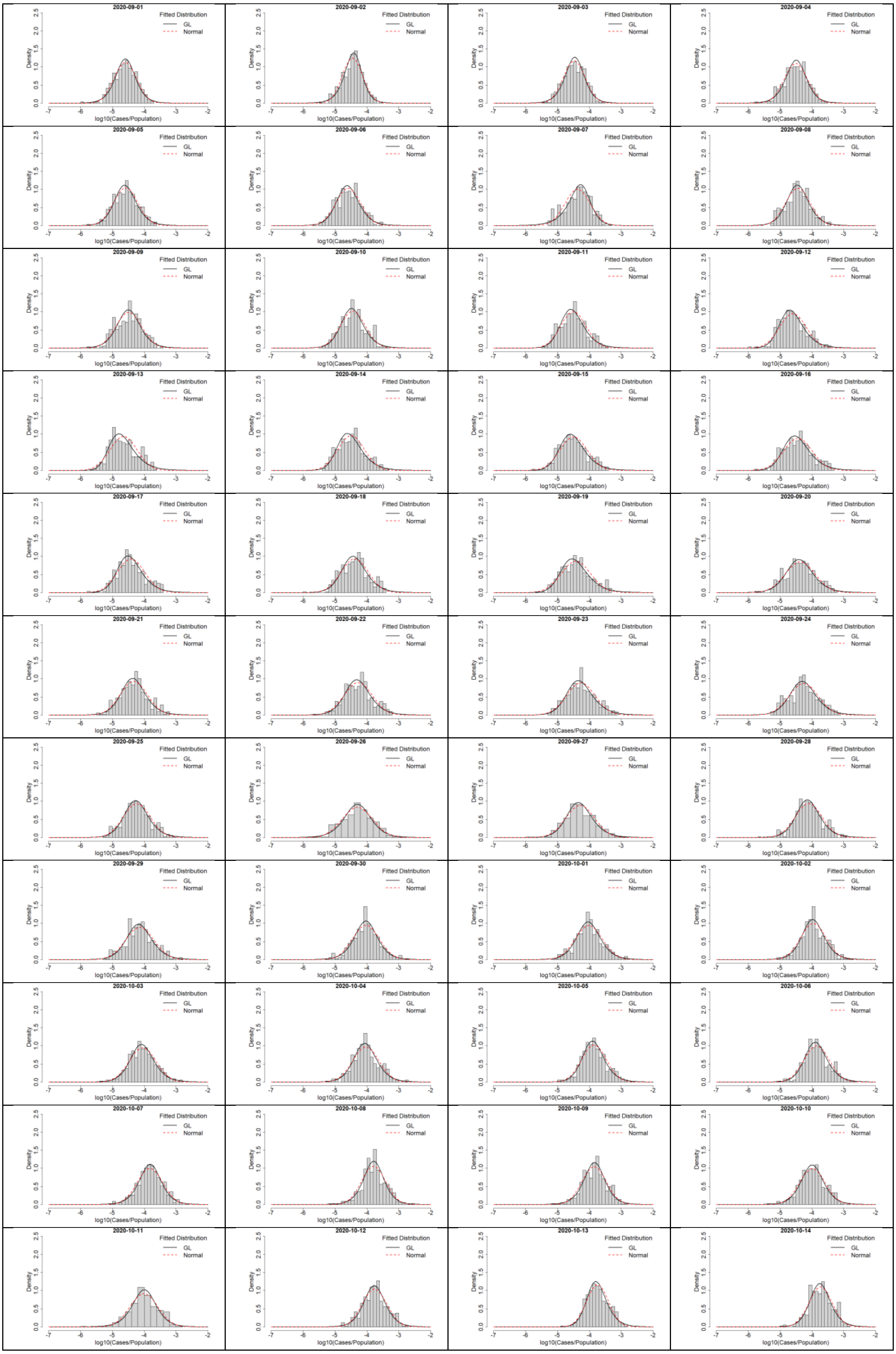
2020-09-01
2020-09-02
2020-09-03
2020-09-04
2020-09-05
2020-09-06
2020-09-07
2020-09-08
2020-09-09
2020-09-10
2020-09-11
2020-09-12
2020-09-13
2020-09-14
2020-09-15
2020-09-16
2020-09-17
2020-09-18
2020-09-19
2020-09-20
2020-09-21
2020-09-22
2020-09-23
2020-09-24
2020-09-25
2020-09-26
2020-09-27
2020-09-28
2020-09-29
2020-09-30
2020-10-01
2020-10-02
2020-10-03
2020-10-04
2020-10-05
2020-10-06
2020-10-07
2020-10-08
2020-10-09
2020-10-10
2020-10-11
2020-10-12
2020-10-13
2020-10-14
Fitted Distribution
GL
Normal
Density
log10(Cases/Population)

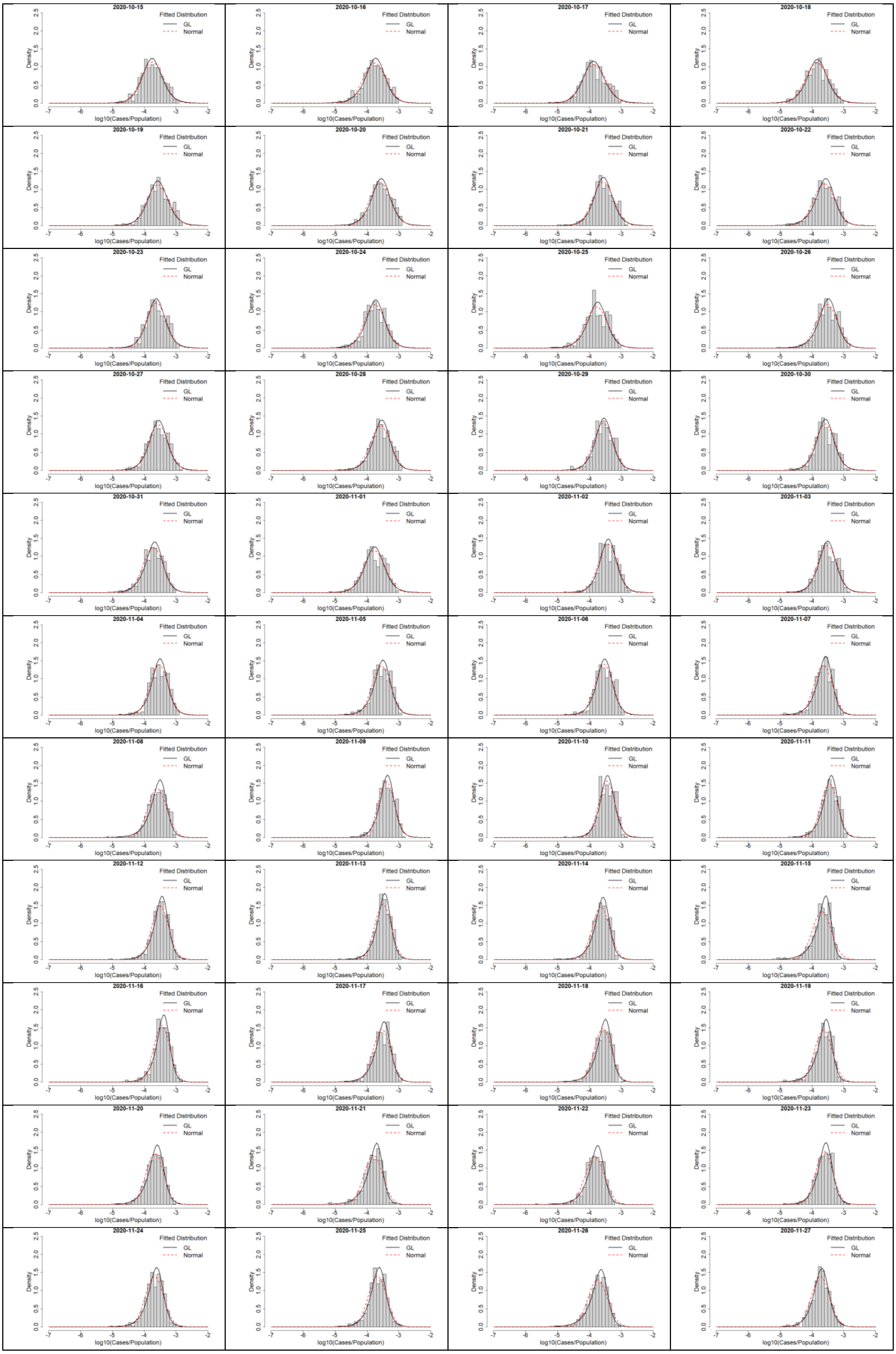

2020-10-15
2020-10-16
2020-10-17
2020-10-18
2020-10-19
2020-10-20
2020-10-21
2020-10-22
2020-10-23
2020-10-24
2020-10-25
2020-10-26
2020-10-27
2020-10-28
2020-10-29
2020-10-30
2020-10-31
2020-11-01
2020-11-02
2020-11-03
2020-11-04
2020-11-05
2020-11-06
2020-11-07
2020-11-08
2020-11-09
2020-11-10
2020-11-11
2020-11-12
2020-11-13
2020-11-14
2020-11-15
2020-11-16
2020-11-17
2020-11-18
2020-11-19
2020-11-20
2020-11-21
2020-11-22
2020-11-23
2020-11-24
2020-11-25
2020-11-26
2020-11-27
Fitted Distribution
GL
Normal
Density
log10(Cases/Population)

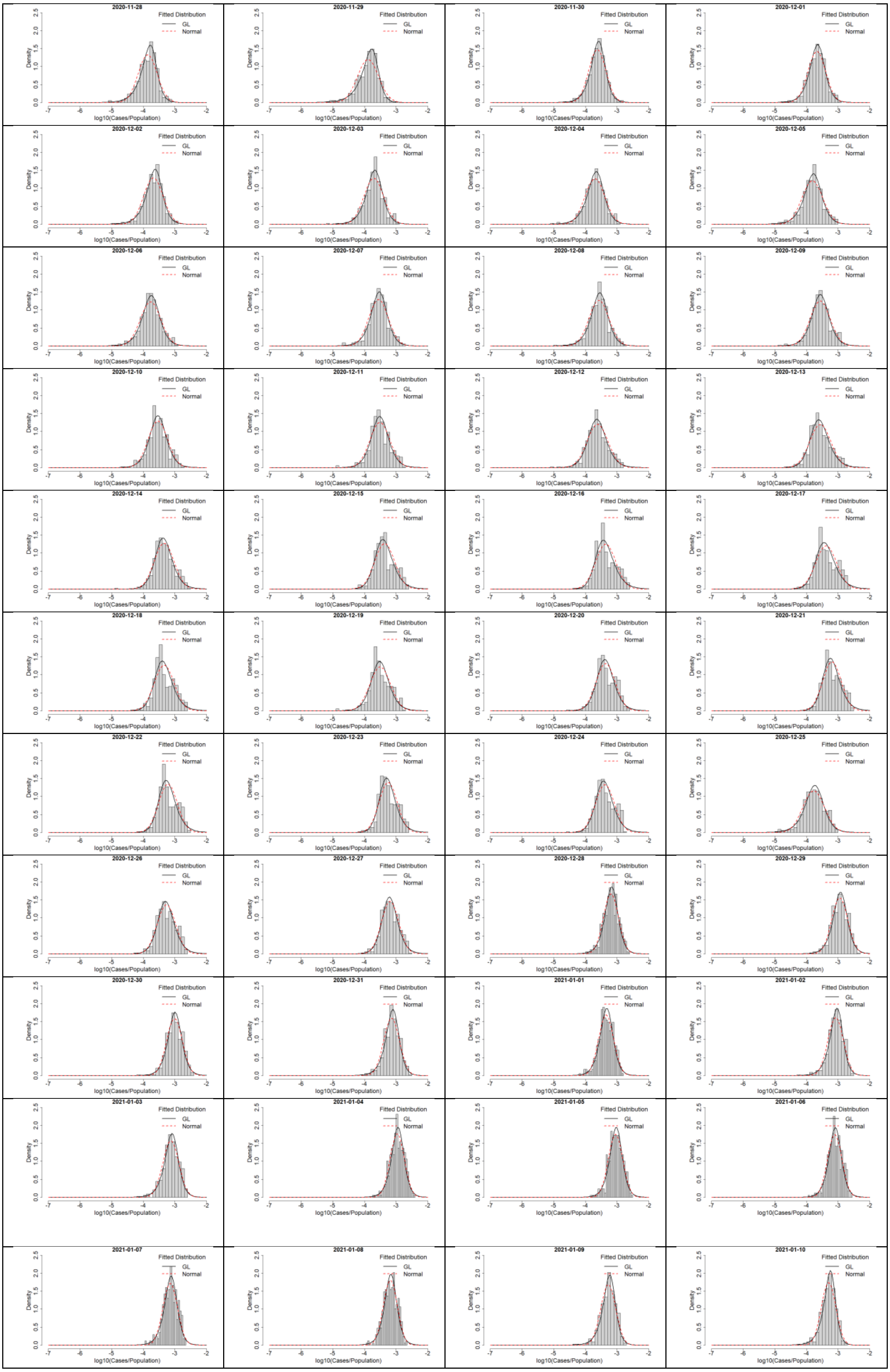

2020-11-28
2020-11-29
2020-11-30
2020-12-01
2020-12-02
2020-12-03
2020-12-04
2020-12-05
2020-12-06
2020-12-07
2020-12-08
2020-12-09
2020-12-10
2020-12-11
2020-12-12
2020-12-13
2020-12-14
2020-12-15
2020-12-16
2020-12-17
2020-12-18
2020-12-19
2020-12-20
2020-12-21
2020-12-22
2020-12-23
2020-12-24
2020-12-25
2020-12-26
2020-12-27
2020-12-28
2020-12-29
2020-12-30
2020-12-31
2021-01-01
2021-01-02
2021-01-03
2021-01-04
2021-01-05
2021-01-06
2021-01-07
2021-01-08
2021-01-09
2021-01-10
Fitted Distribution
GL
Normal
Density
log10(Cases/Population)

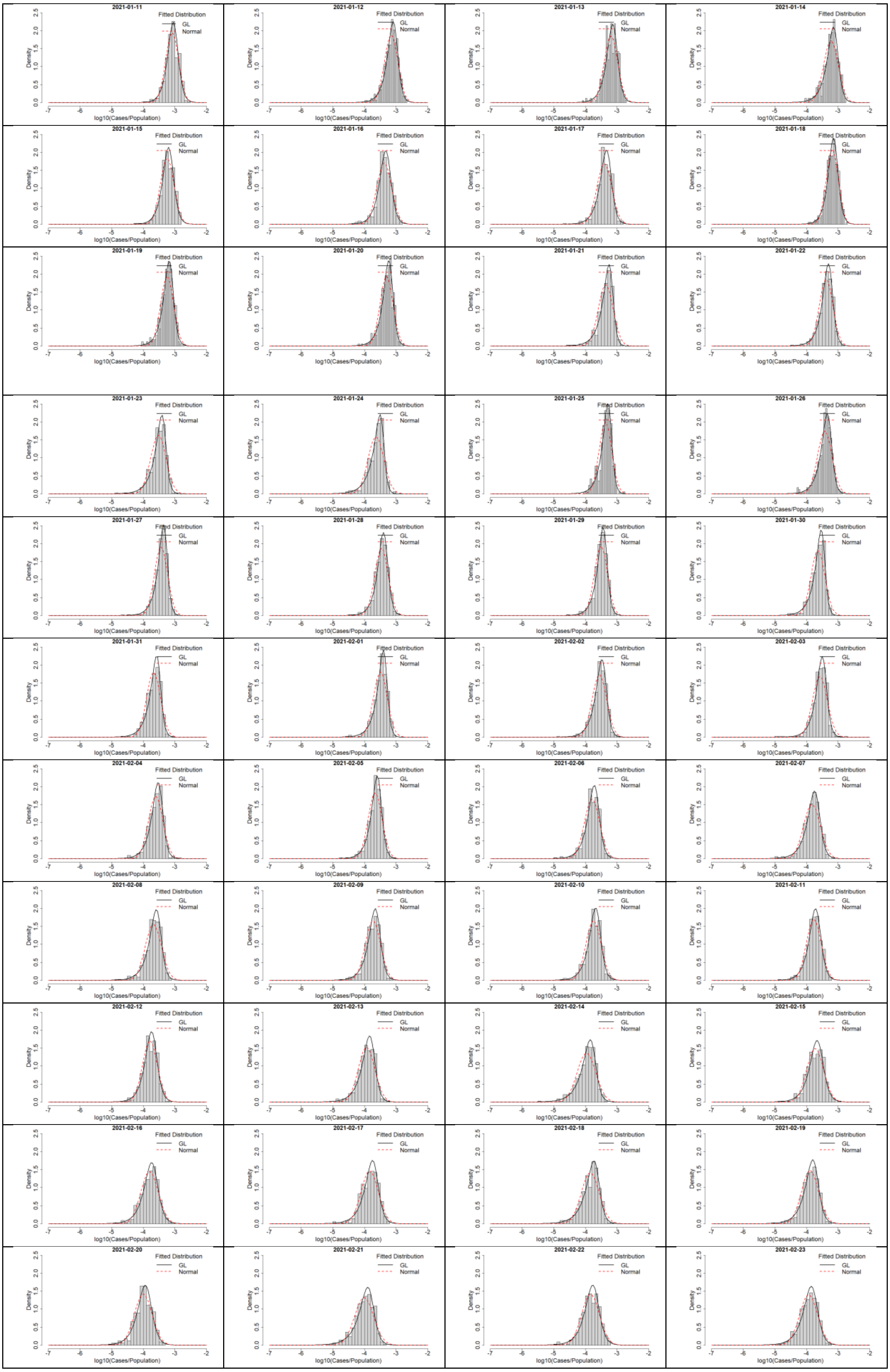
2021-01-11
2021-01-12
2021-01-13
2021-01-14
2021-01-15
2021-01-16
2021-01-17
2021-01-18
2021-01-19
2021-01-20
2021-01-21
2021-01-22
2021-01-23
2021-01-24
2021-01-25
2021-01-26
2021-01-27
2021-01-28
2021-01-29
2021-01-30
2021-01-31
2021-02-01
2021-02-02
2021-02-03
2021-02-04
2021-02-05
2021-02-06
2021-02-07
2021-02-08
2021-02-09
2021-02-10
2021-02-11
2021-02-12
2021-02-13
2021-02-14
2021-02-15
2021-02-16
2021-02-17
2021-02-18
2021-02-19
2021-02-20
2021-02-21
2021-02-22
2021-02-23
Fitted Distribution
GL
Normal
Density
log10(Cases/Population)

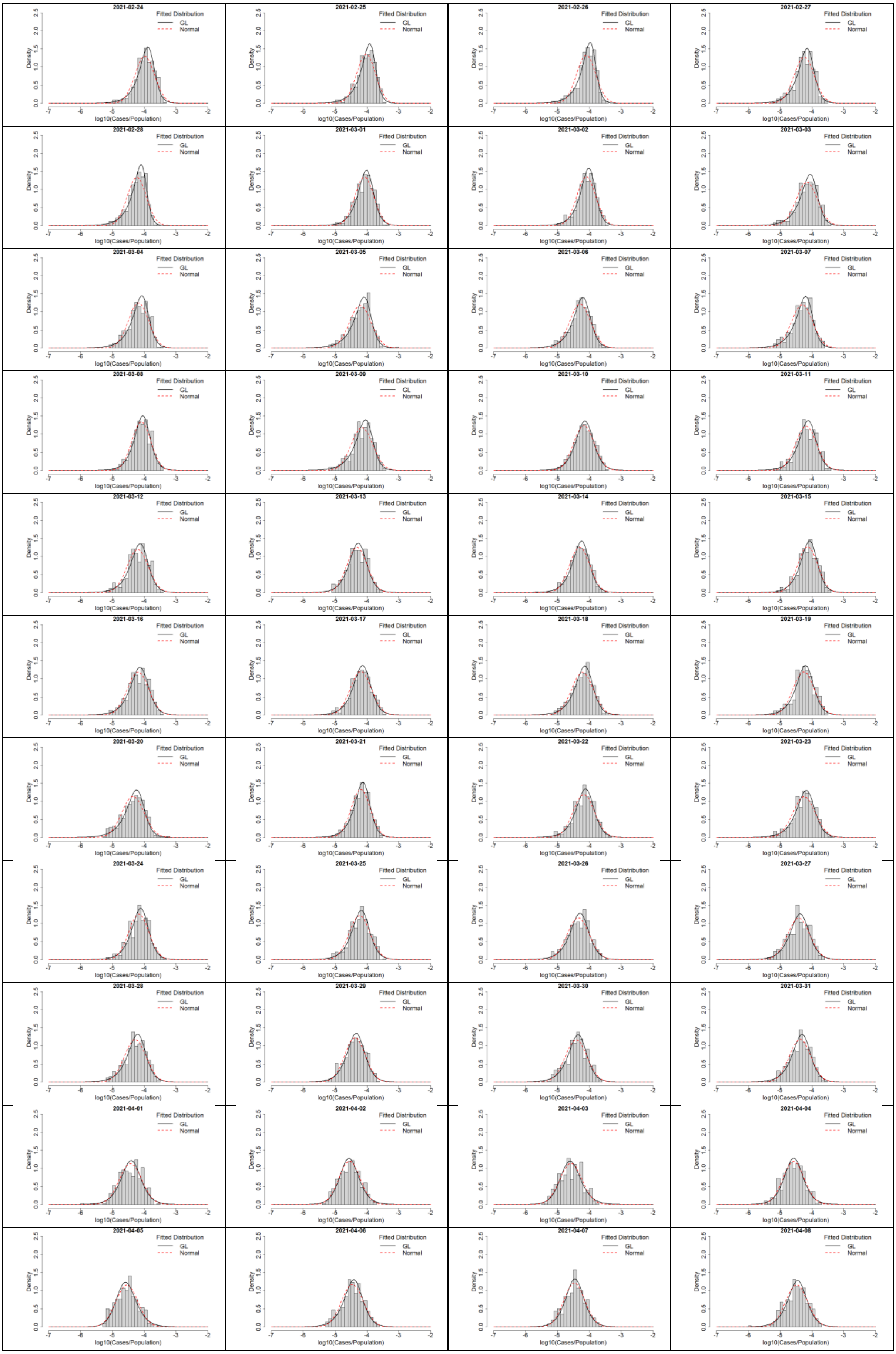
2021-02-24
2021-02-25
2021-02-26
2021-02-27
2021-02-28
2021-03-01
2021-03-02
2021-03-03
2021-03-04
2021-03-05
2021-03-06
2021-03-07
2021-03-08
2021-03-09
2021-03-10
2021-03-11
2021-03-12
2021-03-13
2021-03-14
2021-03-15
2021-03-16
2021-03-17
2021-03-18
2021-03-19
2021-03-20
2021-03-21
2021-03-22
2021-03-23
2021-03-24
2021-03-25
2021-03-26
2021-03-27
2021-03-28
2021-03-29
2021-03-30
2021-03-31
2021-04-01
2021-04-02
2021-04-03
2021-04-04
2021-04-05
2021-04-06
2021-04-07
2021-04-08
Fitted Distribution
GL
Normal
Density
log10(Cases/Population)

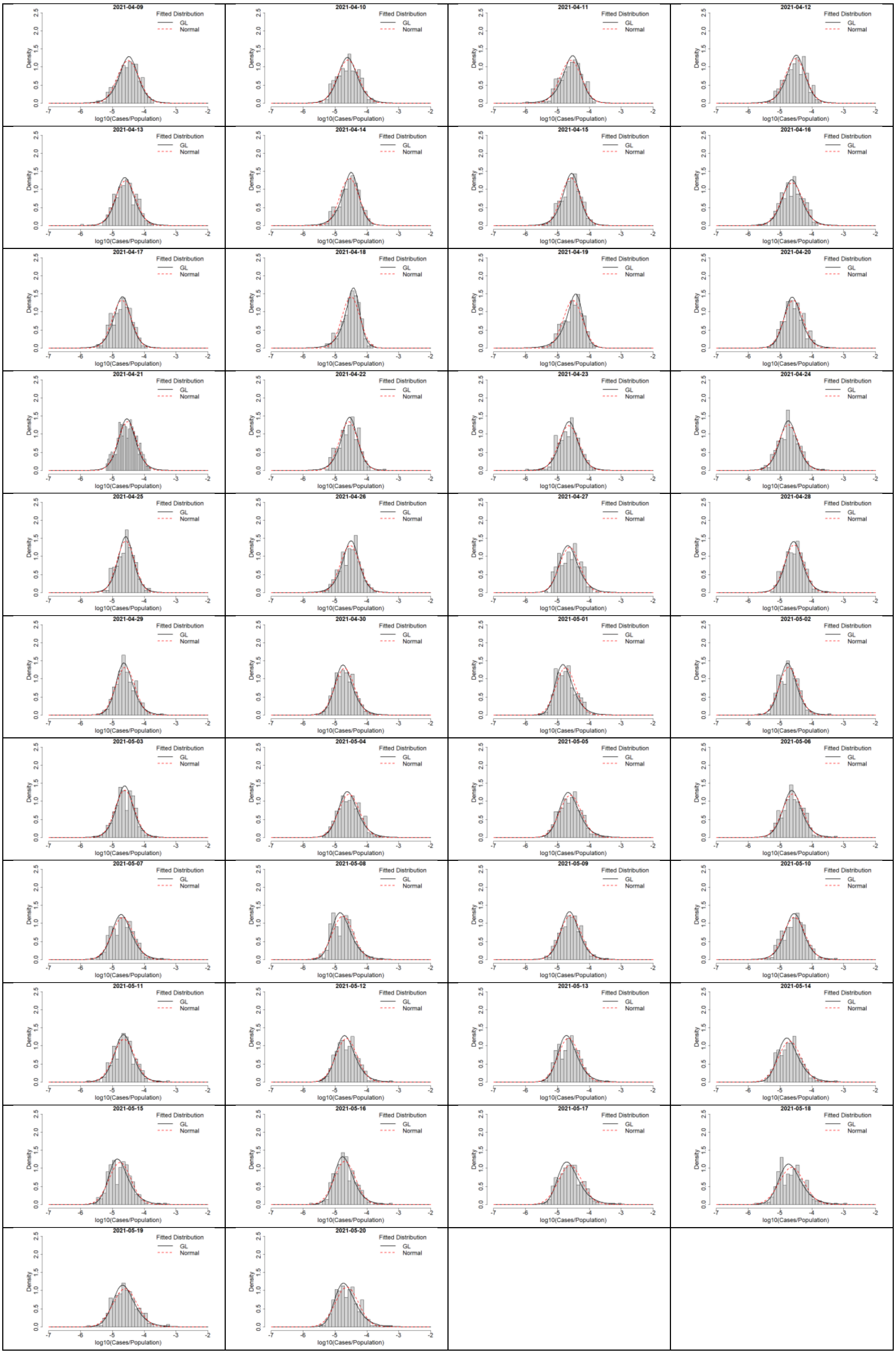
2021-04-09
2021-04-10
2021-04-11
2021-04-12
2021-04-13
2021-04-14
2021-04-15
2021-04-16
2021-04-17
2021-04-18
2021-04-19
2021-04-20
2021-04-21
2021-04-22
2021-04-23
2021-04-24
2021-04-25
2021-04-26
2021-04-27
2021-04-28
2021-04-29
2021-04-30
2021-05-01
2021-05-02
2021-05-03
2021-05-04
2021-05-05
2021-05-06
2021-05-07
2021-05-08
2021-05-09
2021-05-10
2021-05-11
2021-05-12
2021-05-13
2021-05-14
2021-05-15
2021-05-16
2021-05-17
2021-05-18
2021-05-19
2021-05-20
Fitted Distribution
GL
Normal
Density
log10(Cases/Population)

Fig S1. Daily histograms of LTLA COVID-19 cases per capita. Black line represents the generalised logistic distribution and the red dashed line represents the normal distribution.
