# Supplementary material for: Population density and spreading of COVID-19 in England and Wales
Source: PLoS One. 2022 Mar 31;17(3):e0261725. doi: 10.1371/journal.pone.0261725 (PMC8970409; doi:10.1371/journal.pone.0261725)
Supplement: S2 Fig — The blue dots are the empirical values. A red line represents the single exponent power-law fit. (PDF) [file pone.0261725.s002.pdf]

	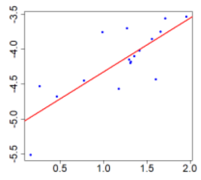	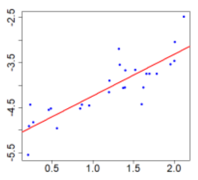	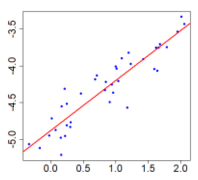	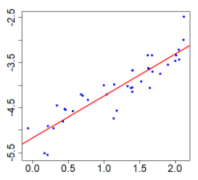	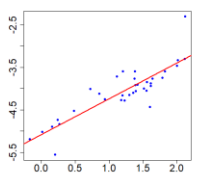
	01/03/2020	02/03/2020	03/03/2020	04/03/2020	05/03/2020
	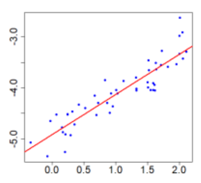	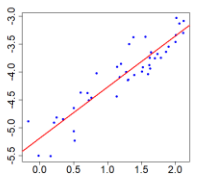	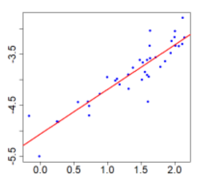	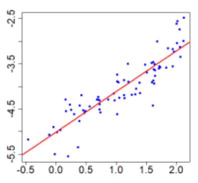	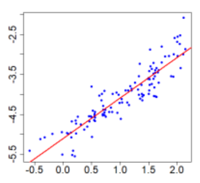
	06/03/2020	07/03/2020	08/03/2020	09/03/2020	10/03/2020

	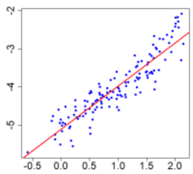	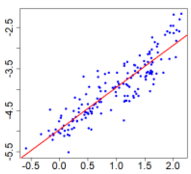	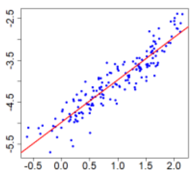	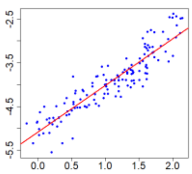	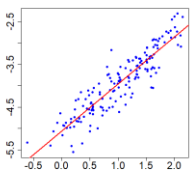
	11/03/2020	12/03/2020	13/03/2020	14/03/2020	15/03/2020
	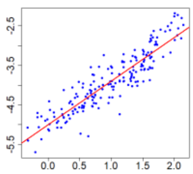	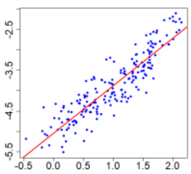	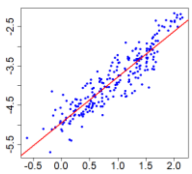	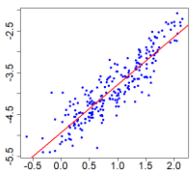	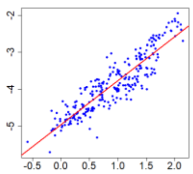
	16/03/2020	17/03/2020	18/03/2020	19/03/2020	20/03/2020
	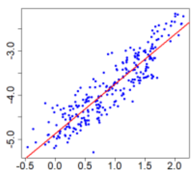	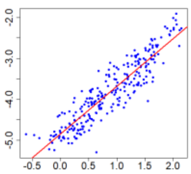	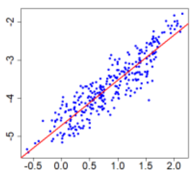	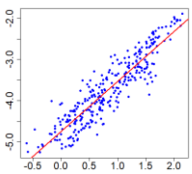	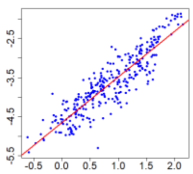
	21/03/2020	22/03/2020	23/03/2020	24/03/2020	25/03/2020
	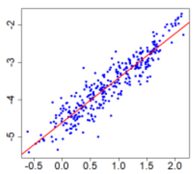	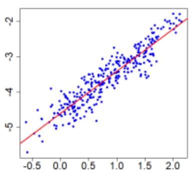	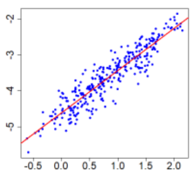	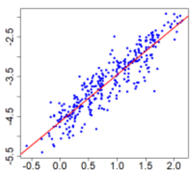	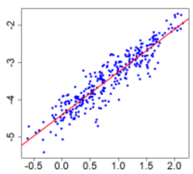
	26/03/2020	27/03/2020	28/03/2020	29/03/2020	30/03/2020
	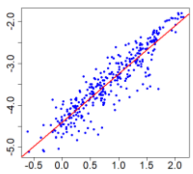	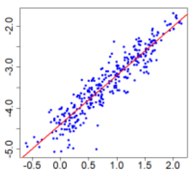	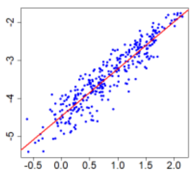	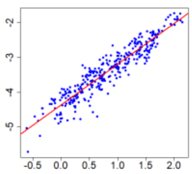	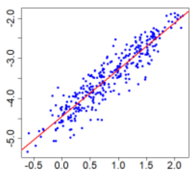
	31/03/2020	01/04/2020	02/04/2020	03/04/2020	04/04/2020
	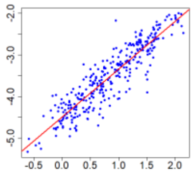	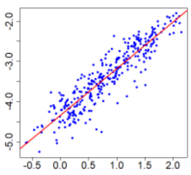	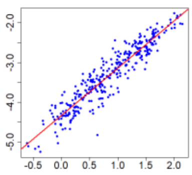	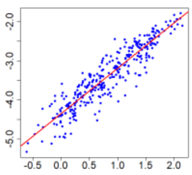	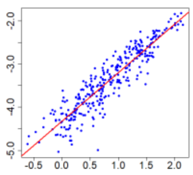
	05/04/2020	06/04/2020	07/04/2020	08/04/2020	09/04/2020

	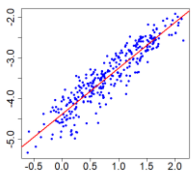	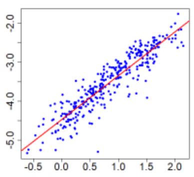	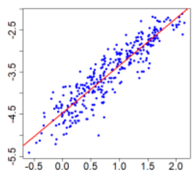	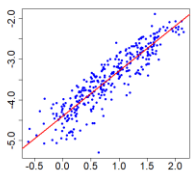	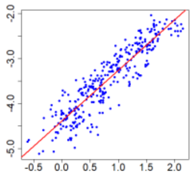
	10/04/2020	11/04/2020	12/04/2020	13/04/2020	14/04/2020
	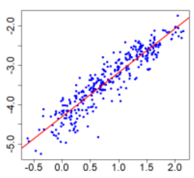	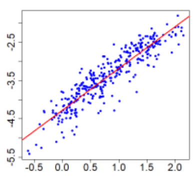	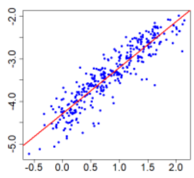	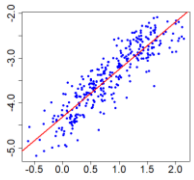	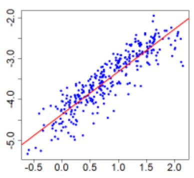
	15/04/2020	16/04/2020	17/04/2020	18/04/2020	19/04/2020
	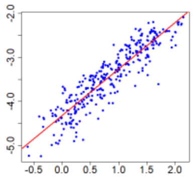	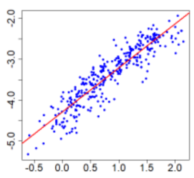	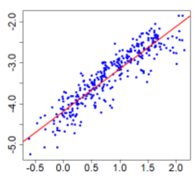	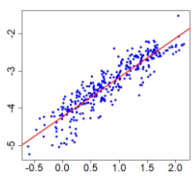	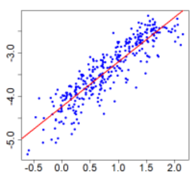
	20/04/2020	21/04/2020	22/04/2020	23/04/2020	24/04/2020
	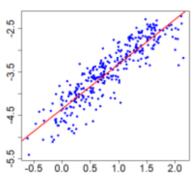	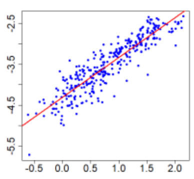	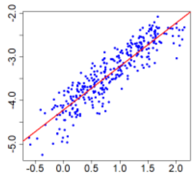	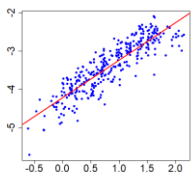	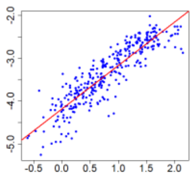
	25/04/2020	26/04/2020	27/04/2020	28/04/2020	29/04/2020
	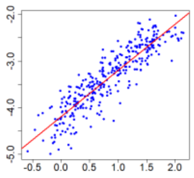	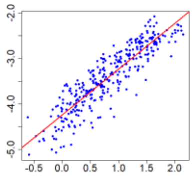	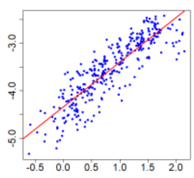	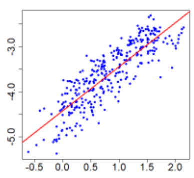	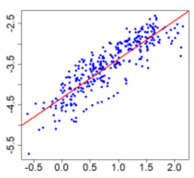
	30/04/2020	01/05/2020	02/05/2020	03/05/2020	04/05/2020
	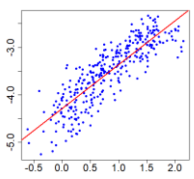	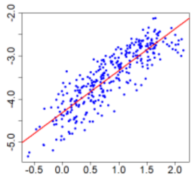	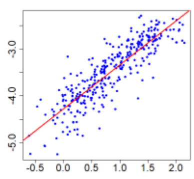	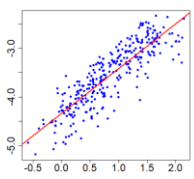	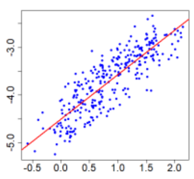
	05/05/2020	06/05/2020	07/05/2020	08/05/2020	09/05/2020

	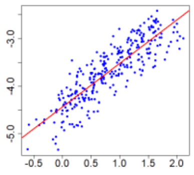	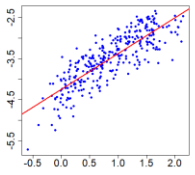	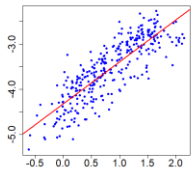	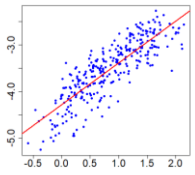	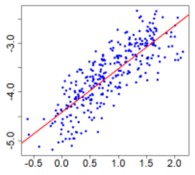
	10/05/2020	11/05/2020	12/05/2020	13/05/2020	14/05/2020
	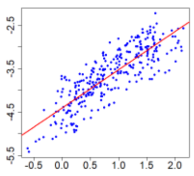	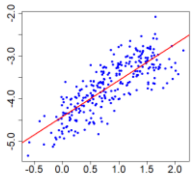	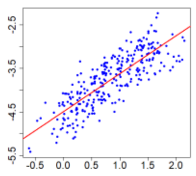	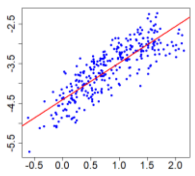	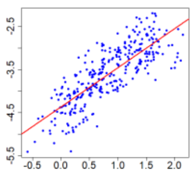
	15/05/2020	16/05/2020	17/05/2020	18/05/2020	19/05/2020
	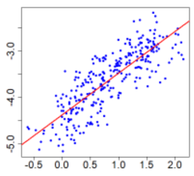	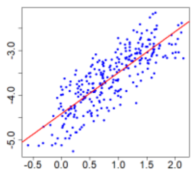	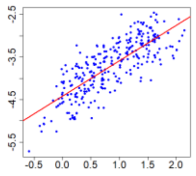	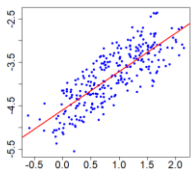	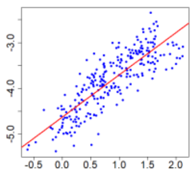
	20/05/2020	21/05/2020	22/05/2020	23/05/2020	24/05/2020
	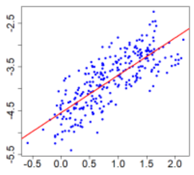	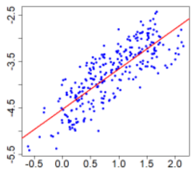	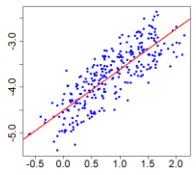	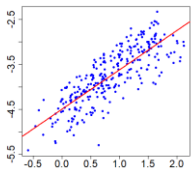	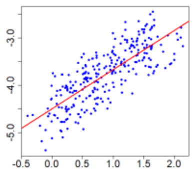
	25/05/2020	26/05/2020	27/05/2020	28/05/2020	29/05/2020
	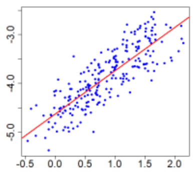	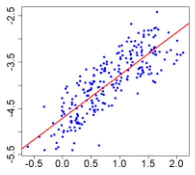	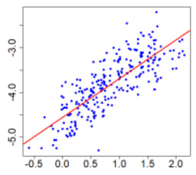	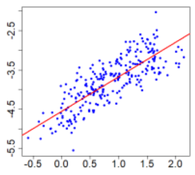	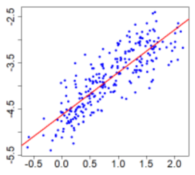
	30/05/2020	31/05/2020	01/06/2020	02/06/2020	03/06/2020
	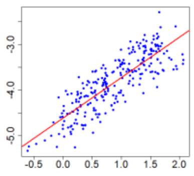	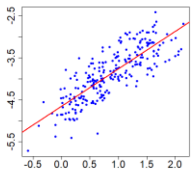	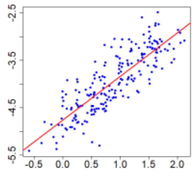	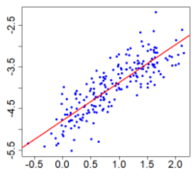	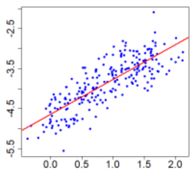
	04/06/2020	05/06/2020	06/06/2020	07/06/2020	08/06/2020

	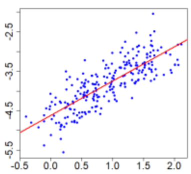	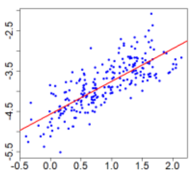	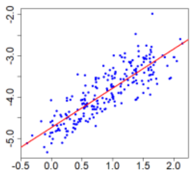	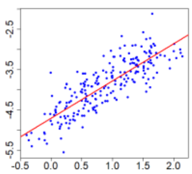	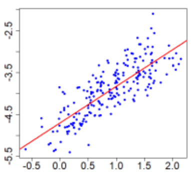
	09/06/2020	10/06/2020	11/06/2020	12/06/2020	13/06/2020
	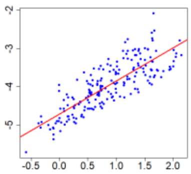	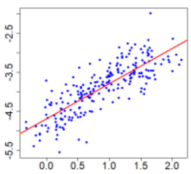	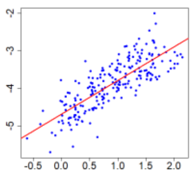	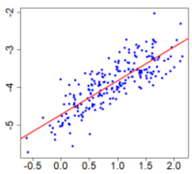	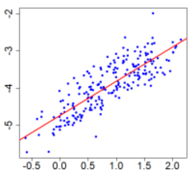
	14/06/2020	15/06/2020	16/06/2020	17/06/2020	18/06/2020
	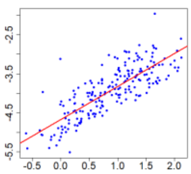	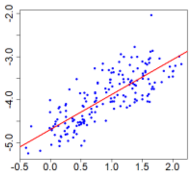	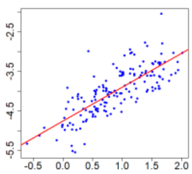	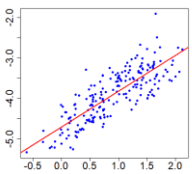	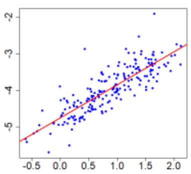
	19/06/2020	20/06/2020	21/06/2020	22/06/2020	23/06/2020
	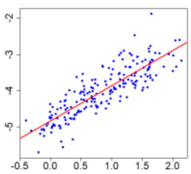	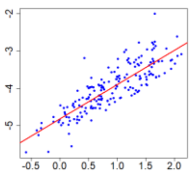	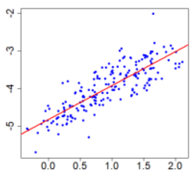	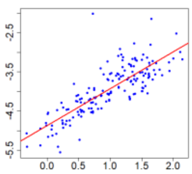	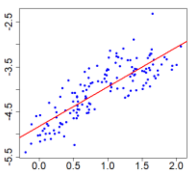
	24/06/2020	25/06/2020	26/06/2020	27/06/2020	28/06/2020
	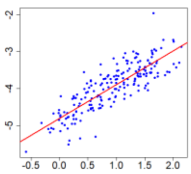	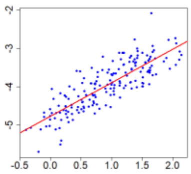	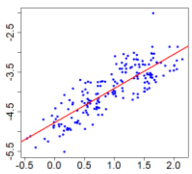	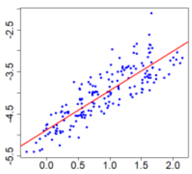	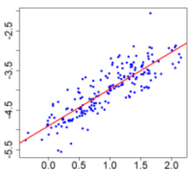
	29/06/2020	30/06/2020	01/07/2020	02/07/2020	03/07/2020
	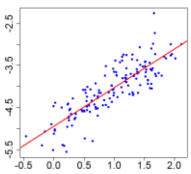	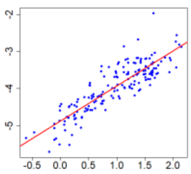	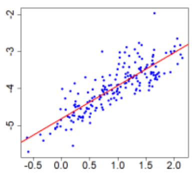	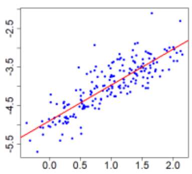	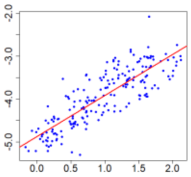
	04/07/2020	05/07/2020	06/07/2020	07/07/2020	08/07/2020

	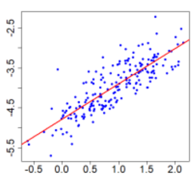	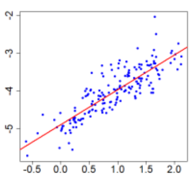	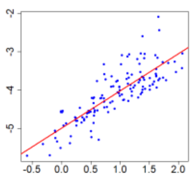	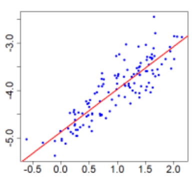	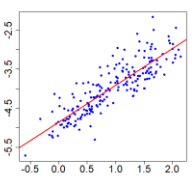
	09/07/2020	10/07/2020	11/07/2020	12/07/2020	13/07/2020
	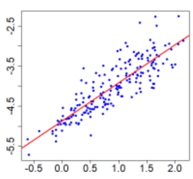	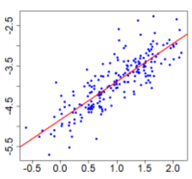	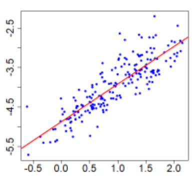	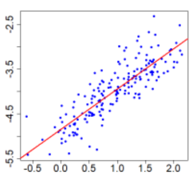	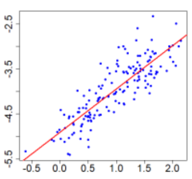
	14/07/2020	15/07/2020	16/07/2020	17/07/2020	18/07/2020
	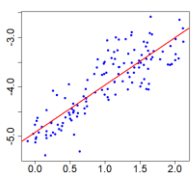	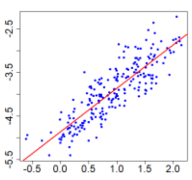	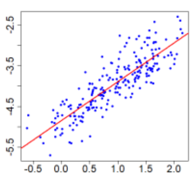	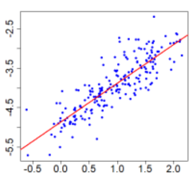	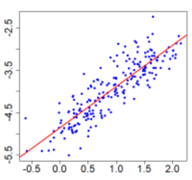
	19/07/2020	20/07/2020	21/07/2020	22/07/2020	23/07/2020
	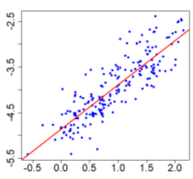	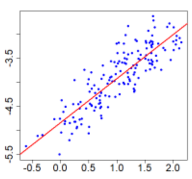	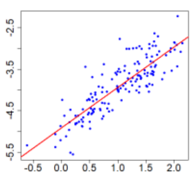	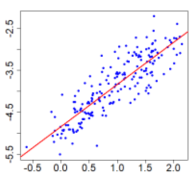	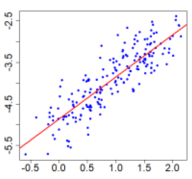
	24/07/2020	25/07/2020	26/07/2020	27/07/2020	28/07/2020
	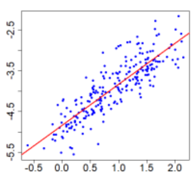	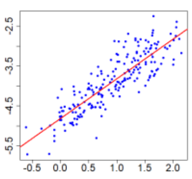	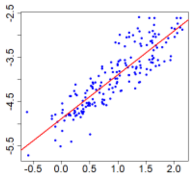	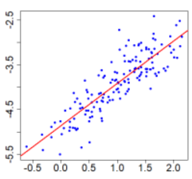	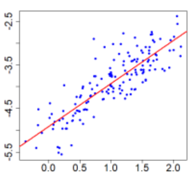
	29/07/2020	30/07/2020	31/07/2020	01/08/2020	02/08/2020
	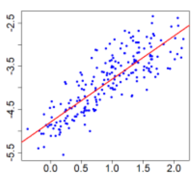	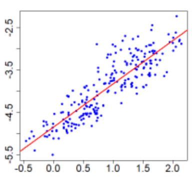	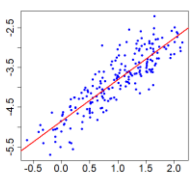	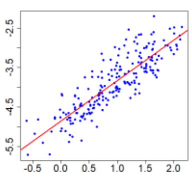	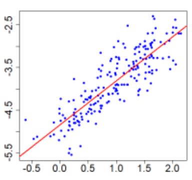
	03/08/2020	04/08/2020	05/08/2020	06/08/2020	07/08/2020

	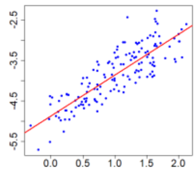	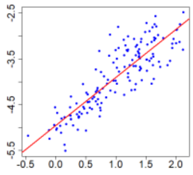	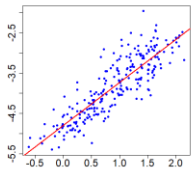	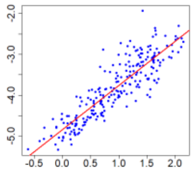	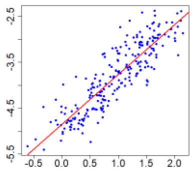
	08/08/2020	09/08/2020	10/08/2020	11/08/2020	12/08/2020
	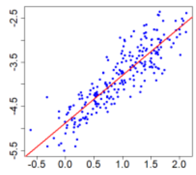	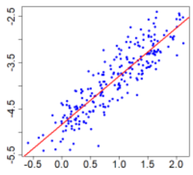	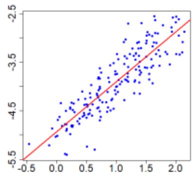	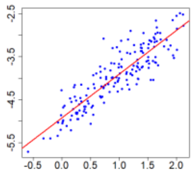	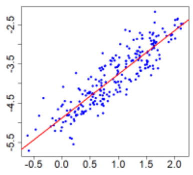
	13/08/2020	14/08/2020	15/08/2020	16/08/2020	17/08/2020
	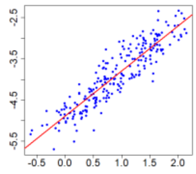	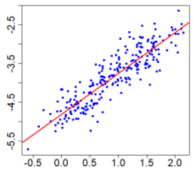	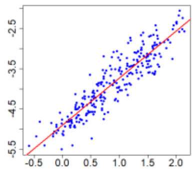	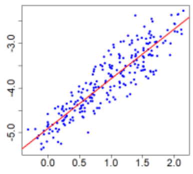	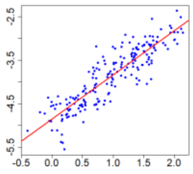
	18/08/2020	19/08/2020	20/08/2020	21/08/2020	22/08/2020
	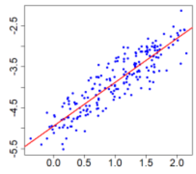	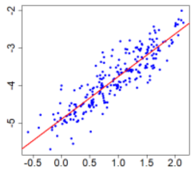	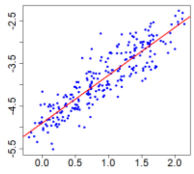	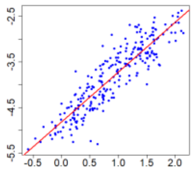	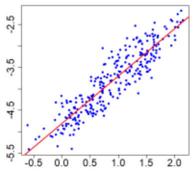
	23/08/2020	24/08/2020	25/08/2020	26/08/2020	27/08/2020
	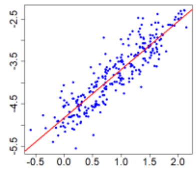	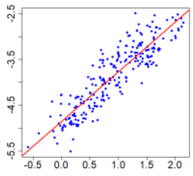	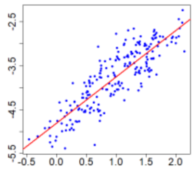	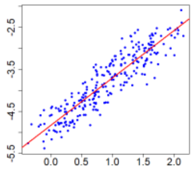	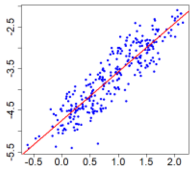
	28/08/2020	29/08/2020	30/08/2020	31/08/2020	01/09/2020
	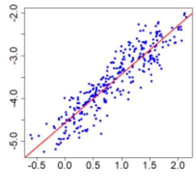	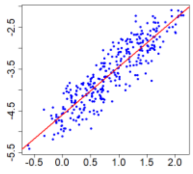	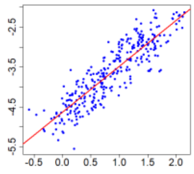	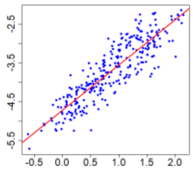	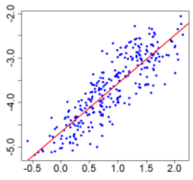
	02/09/2020	03/09/2020	04/09/2020	05/09/2020	06/09/2020

	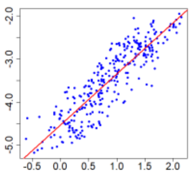	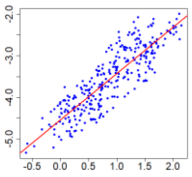	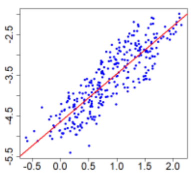	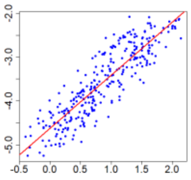	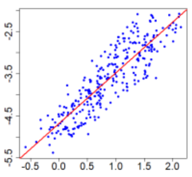
	07/09/2020	08/09/2020	09/09/2020	10/09/2020	11/09/2020
	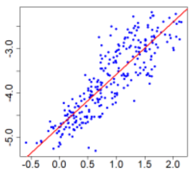	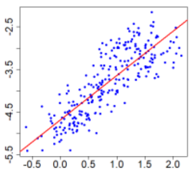	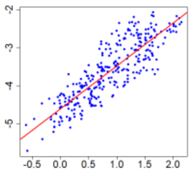	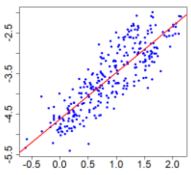	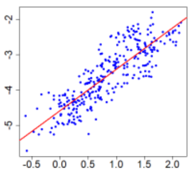
	12/09/2020	13/09/2020	14/09/2020	15/09/2020	16/09/2020
	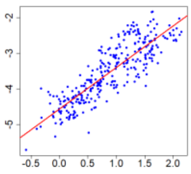	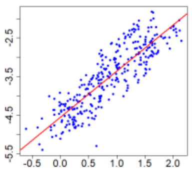	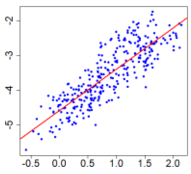	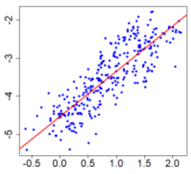	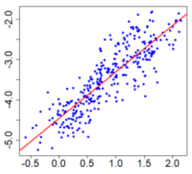
	17/09/2020	18/09/2020	19/09/2020	20/09/2020	21/09/2020
	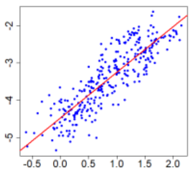	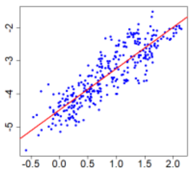	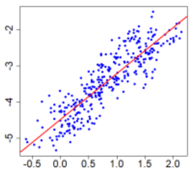	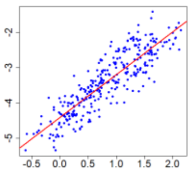	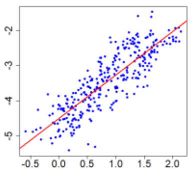
	22/09/2020	23/09/2020	24/09/2020	25/09/2020	26/09/2020
	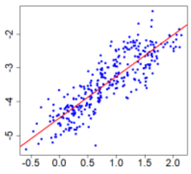	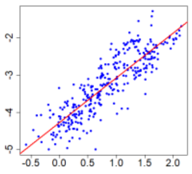	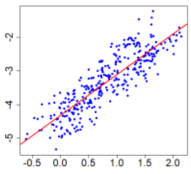	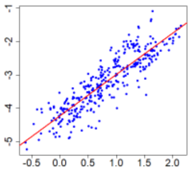	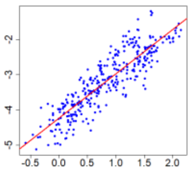
	27/09/2020	28/09/2020	29/09/2020	30/09/2020	01/10/2020
	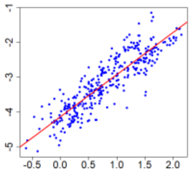	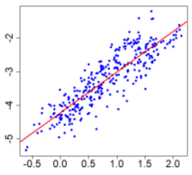	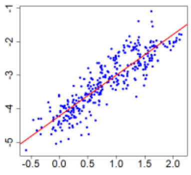	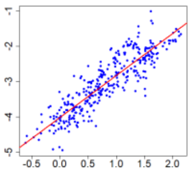	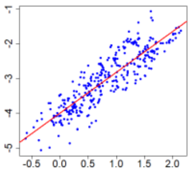
	02/10/2020	03/10/2020	04/10/2020	05/10/2020	06/10/2020

	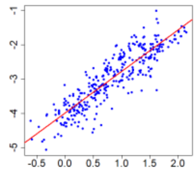	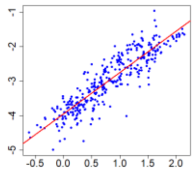	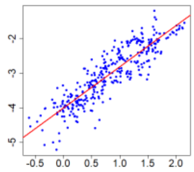	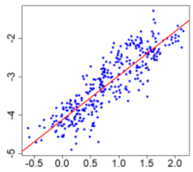	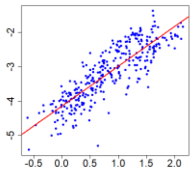
	07/10/2020	08/10/2020	09/10/2020	10/10/2020	11/10/2020
	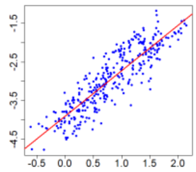	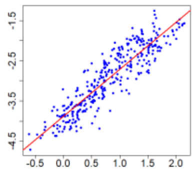	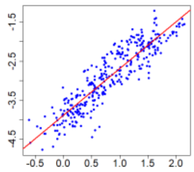	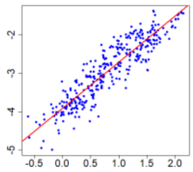	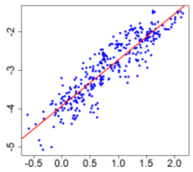
	12/10/2020	13/10/2020	14/10/2020	15/10/2020	16/10/2020
	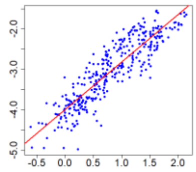	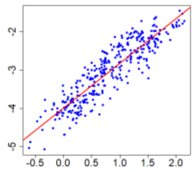	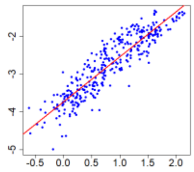	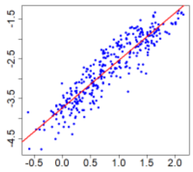	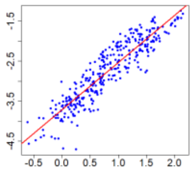
	17/10/2020	18/10/2020	19/10/2020	20/10/2020	21/10/2020
	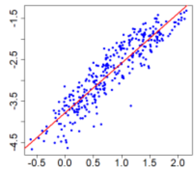	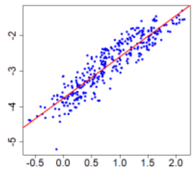	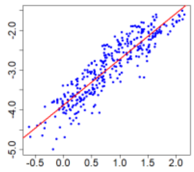	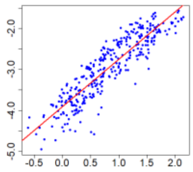	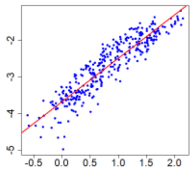
	22/10/2020	23/10/2020	24/10/2020	25/10/2020	26/10/2020
	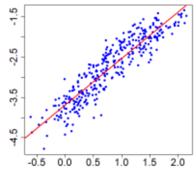	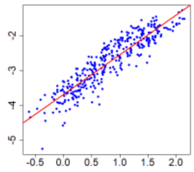	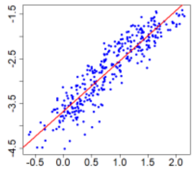	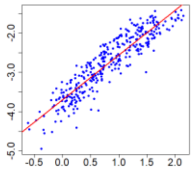	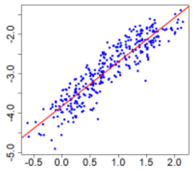
	27/10/2020	28/10/2020	29/10/2020	30/10/2020	31/10/2020
	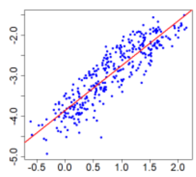	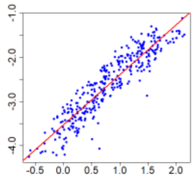	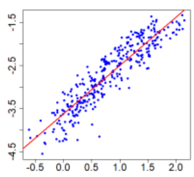	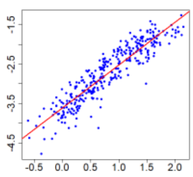	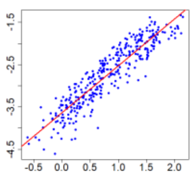
	01/11/2020	02/11/2020	03/11/2020	04/11/2020	05/11/2020

	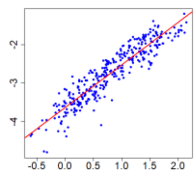	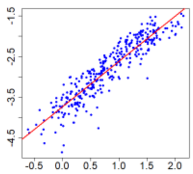	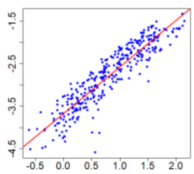	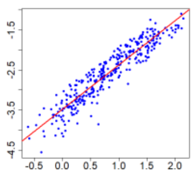	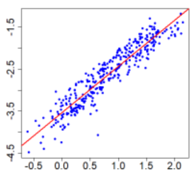
	06/11/2020	07/11/2020	08/11/2020	09/11/2020	10/11/2020
	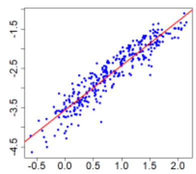	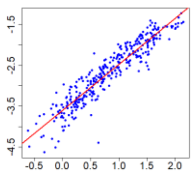	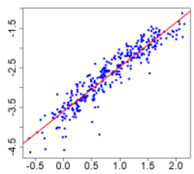	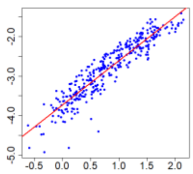	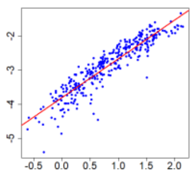
	11/11/2020	12/11/2020	13/11/2020	14/11/2020	15/11/2020
	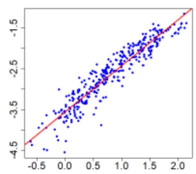	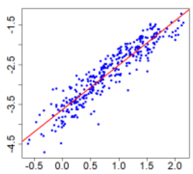	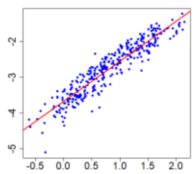	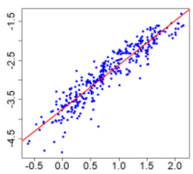	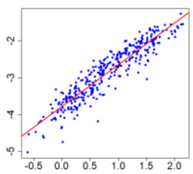
	16/11/2020	17/11/2020	18/11/2020	19/11/2020	20/11/2020
	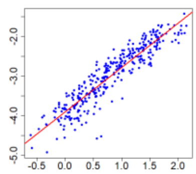	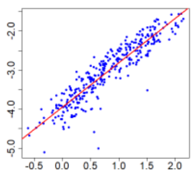	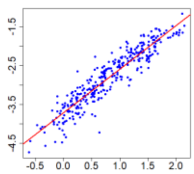	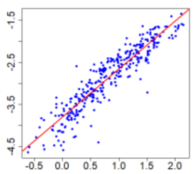	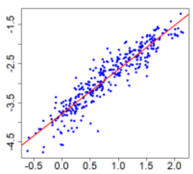
	21/11/2020	22/11/2020	23/11/2020	24/11/2020	25/11/2020
	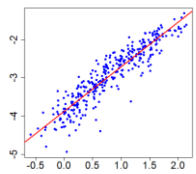	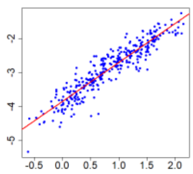	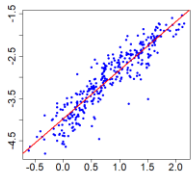	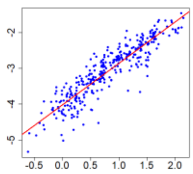	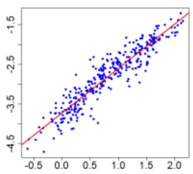
	26/11/2020	27/11/2020	28/11/2020	29/11/2020	30/11/2020
	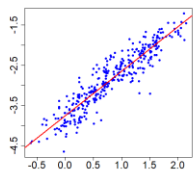	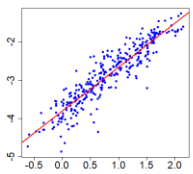	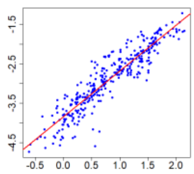	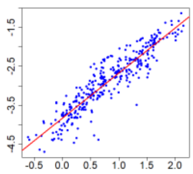	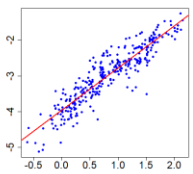
	01/12/2020	02/12/2020	03/12/2020	04/12/2020	05/12/2020

	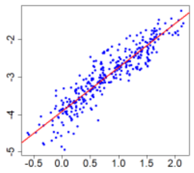	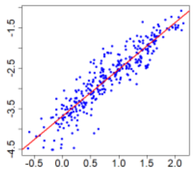	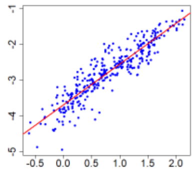	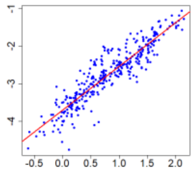	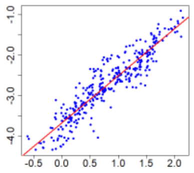
	06/12/2020	07/12/2020	08/12/2020	09/12/2020	10/12/2020
	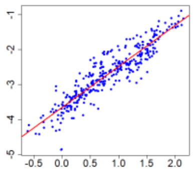	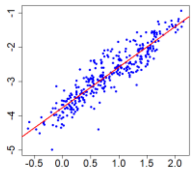	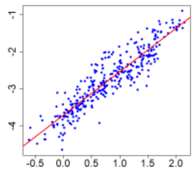	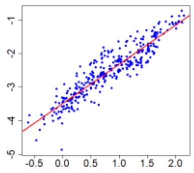	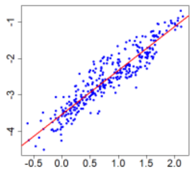
	11/12/2020	12/12/2020	13/12/2020	14/12/2020	15/12/2020
	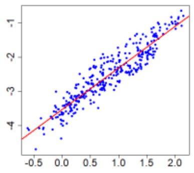	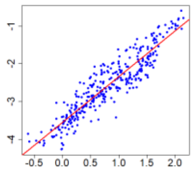	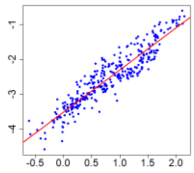	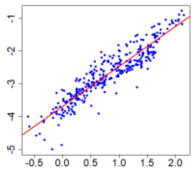	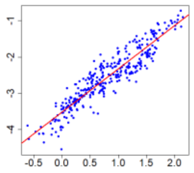
	16/12/2020	17/12/2020	18/12/2020	19/12/2020	20/12/2020
	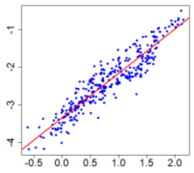	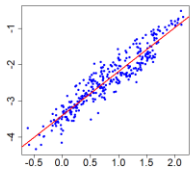	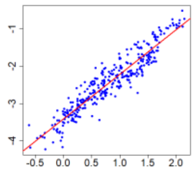	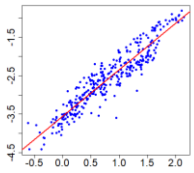	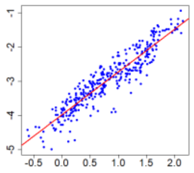
	21/12/2020	22/12/2020	23/12/2020	24/12/2020	25/12/2020
	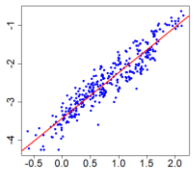	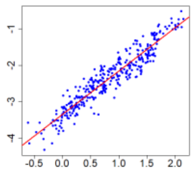	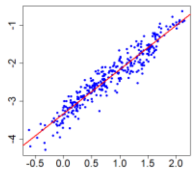	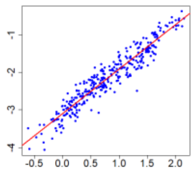	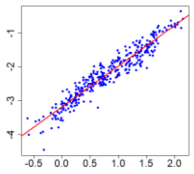
	26/12/2020	27/12/2020	28/12/2020	29/12/2020	30/12/2020
	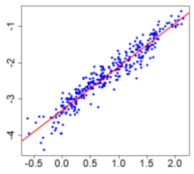	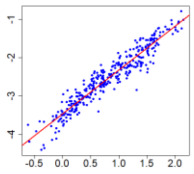	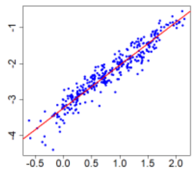	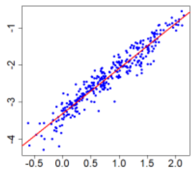	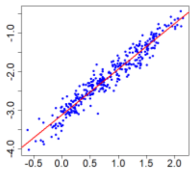
	31/12/2020	01/01/2021	02/01/2021	03/01/2021	04/01/2021

	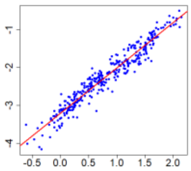	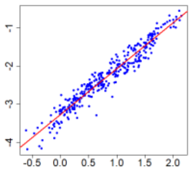	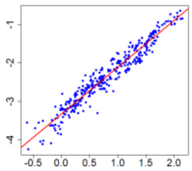	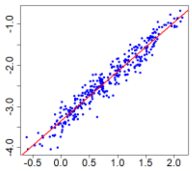	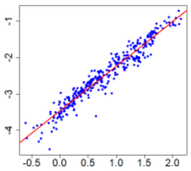
	05/01/2021	06/01/2021	07/01/2021	08/01/2021	09/01/2021
	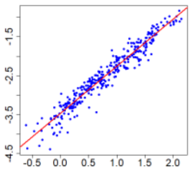	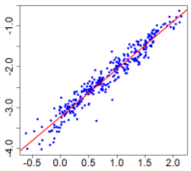	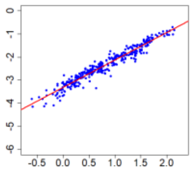	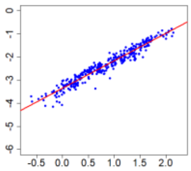	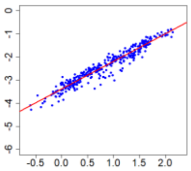
	10/01/2021	11/01/2021	12/01/2021	13/01/2021	14/01/2021
	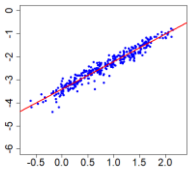	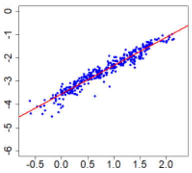	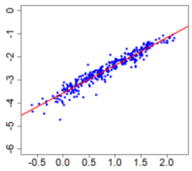	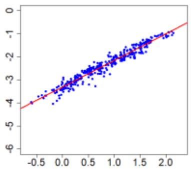	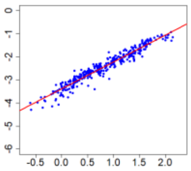
	15/01/2021	16/01/2021	17/01/2021	18/01/2021	19/01/2021
	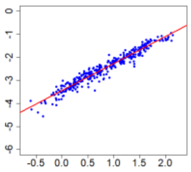	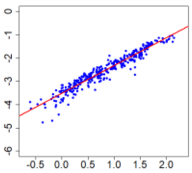	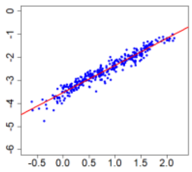	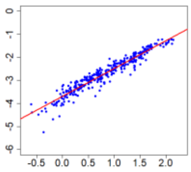	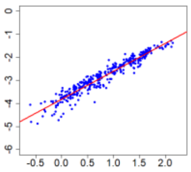
	20/01/2021	21/01/2021	22/01/2021	23/01/2021	24/01/2021
	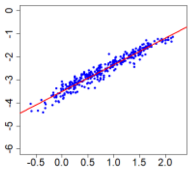	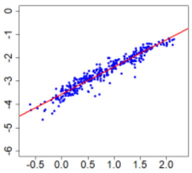	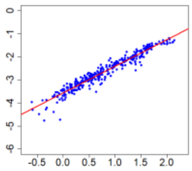	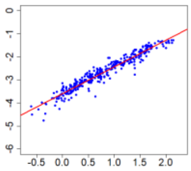	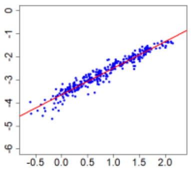
	25/01/2021	26/01/2021	27/01/2021	28/01/2021	29/01/2021
	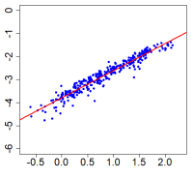	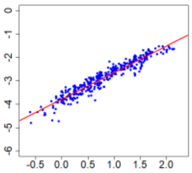	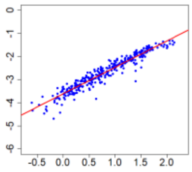	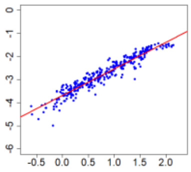	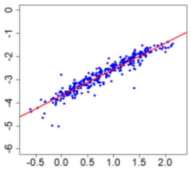
	30/01/2021	31/01/2021	01/02/2021	02/02/2021	03/02/2021

	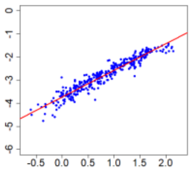	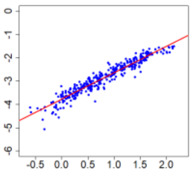	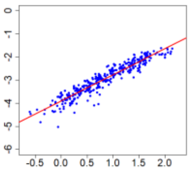	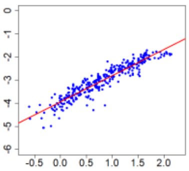	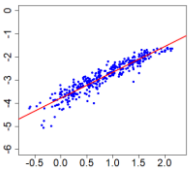
	04/02/2021	05/02/2021	06/02/2021	07/02/2021	08/02/2021
	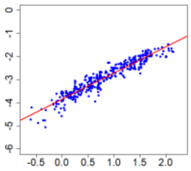	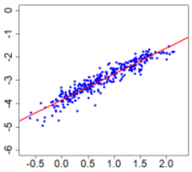	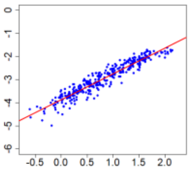	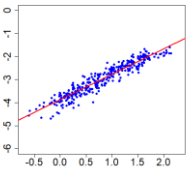	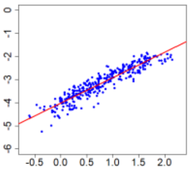
	09/02/2021	10/02/2021	11/02/2021	12/02/2021	13/02/2021
	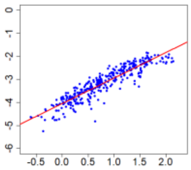	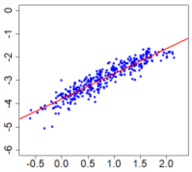	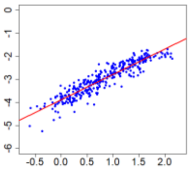	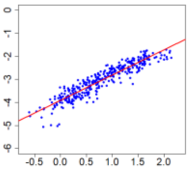	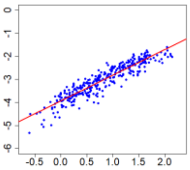
	14/02/2021	15/02/2021	16/02/2021	17/02/2021	18/02/2021
	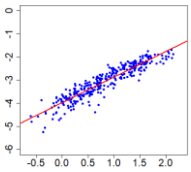	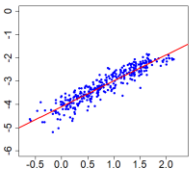	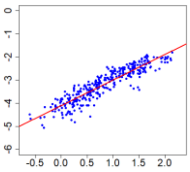	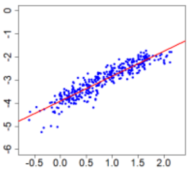	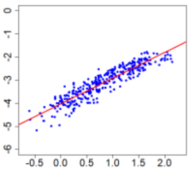
	19/02/2021	20/02/2021	21/02/2021	22/02/2021	23/02/2021
	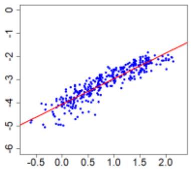	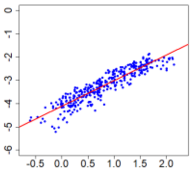	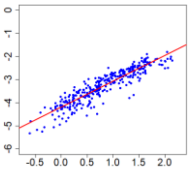	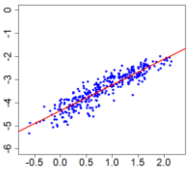	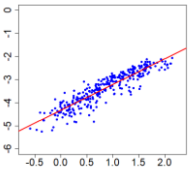
	24/02/2021	25/02/2021	26/02/2021	27/02/2021	28/02/2021
	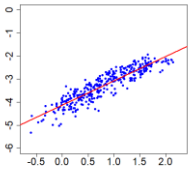	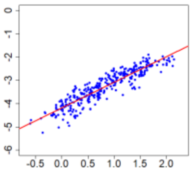	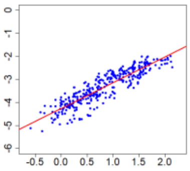	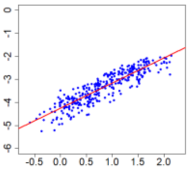	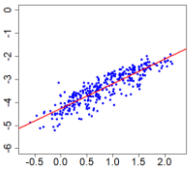
	01/03/2021	02/03/2021	03/03/2021	04/03/2021	05/03/2021

	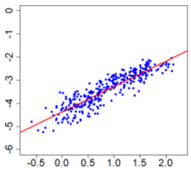	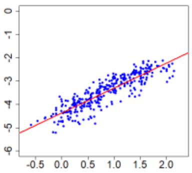	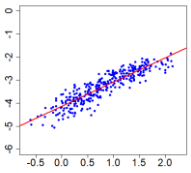	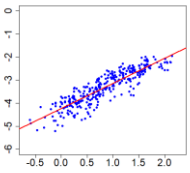	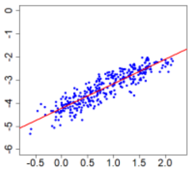
	06/03/2021	07/03/2021	08/03/2021	09/03/2021	10/03/2021
	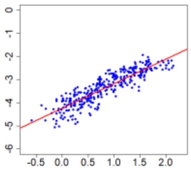	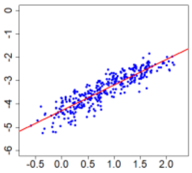	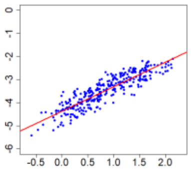	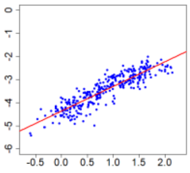	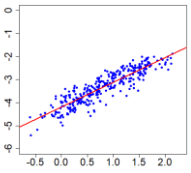
	11/03/2021	12/03/2021	13/03/2021	14/03/2021	15/03/2021
	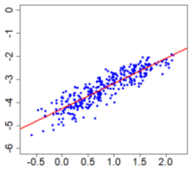	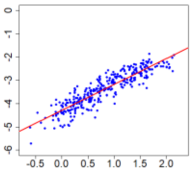	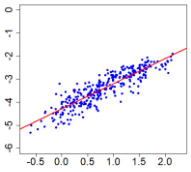	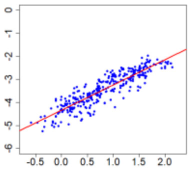	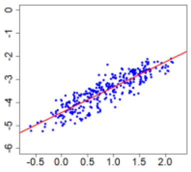
	16/03/2021	17/03/2021	18/03/2021	19/03/2021	20/03/2021
	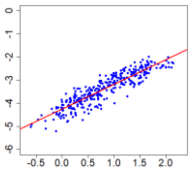	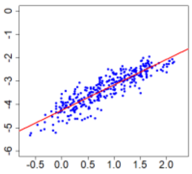	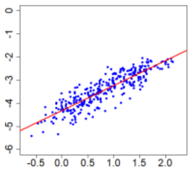	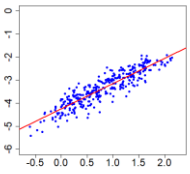	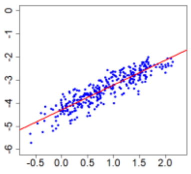
	21/03/2021	22/03/2021	23/03/2021	24/03/2021	25/03/2021
	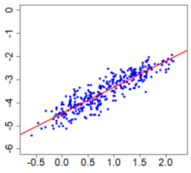	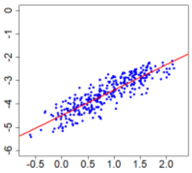	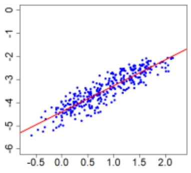	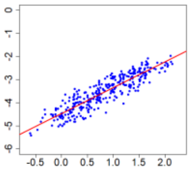	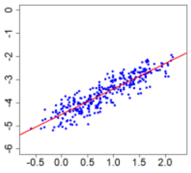
	26/03/2021	27/03/2021	28/03/2021	29/03/2021	30/03/2021
	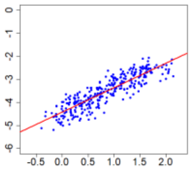	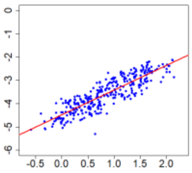	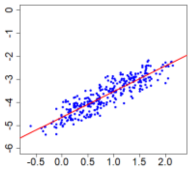	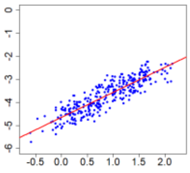	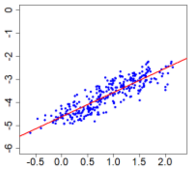
	31/03/2021	01/04/2021	02/04/2021	03/04/2021	04/04/2021

	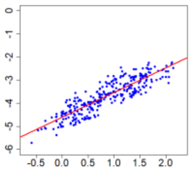	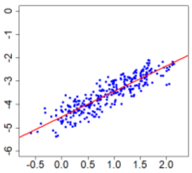	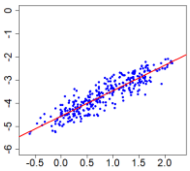	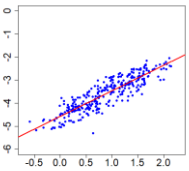	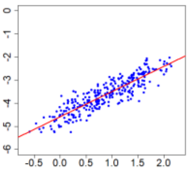
	05/04/2021	06/04/2021	07/04/2021	08/04/2021	09/04/2021
	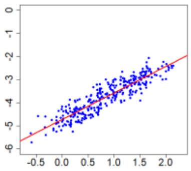	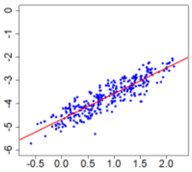	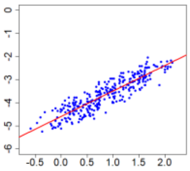	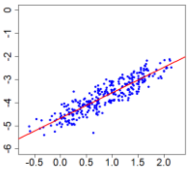	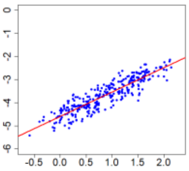
	10/04/2021	11/04/2021	12/04/2021	13/04/2021	14/04/2021
	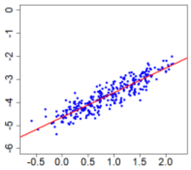	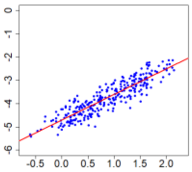	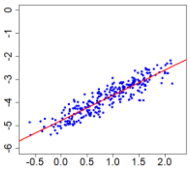	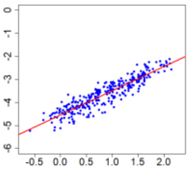	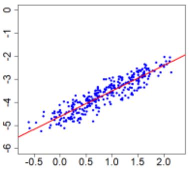
	15/04/2021	16/04/2021	17/04/2021	18/04/2021	19/04/2021
	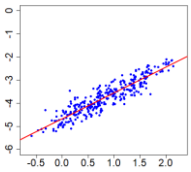	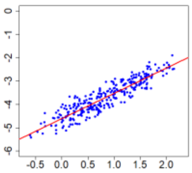	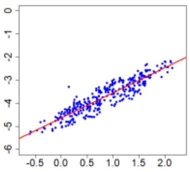	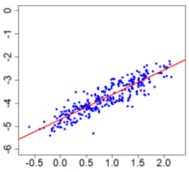	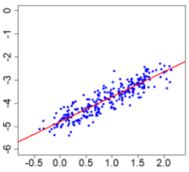
	20/04/2021	21/04/2021	22/04/2021	23/04/2021	24/04/2021
	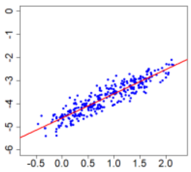	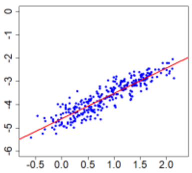	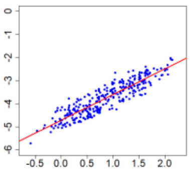	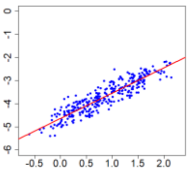	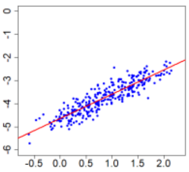
	25/04/2021	26/04/2021	27/04/2021	28/04/2021	29/04/2021
	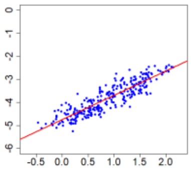	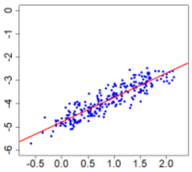	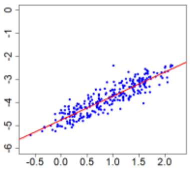	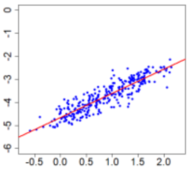	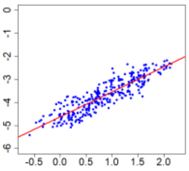
	30/04/2021	01/05/2021	02/05/2021	03/05/2021	04/05/2021

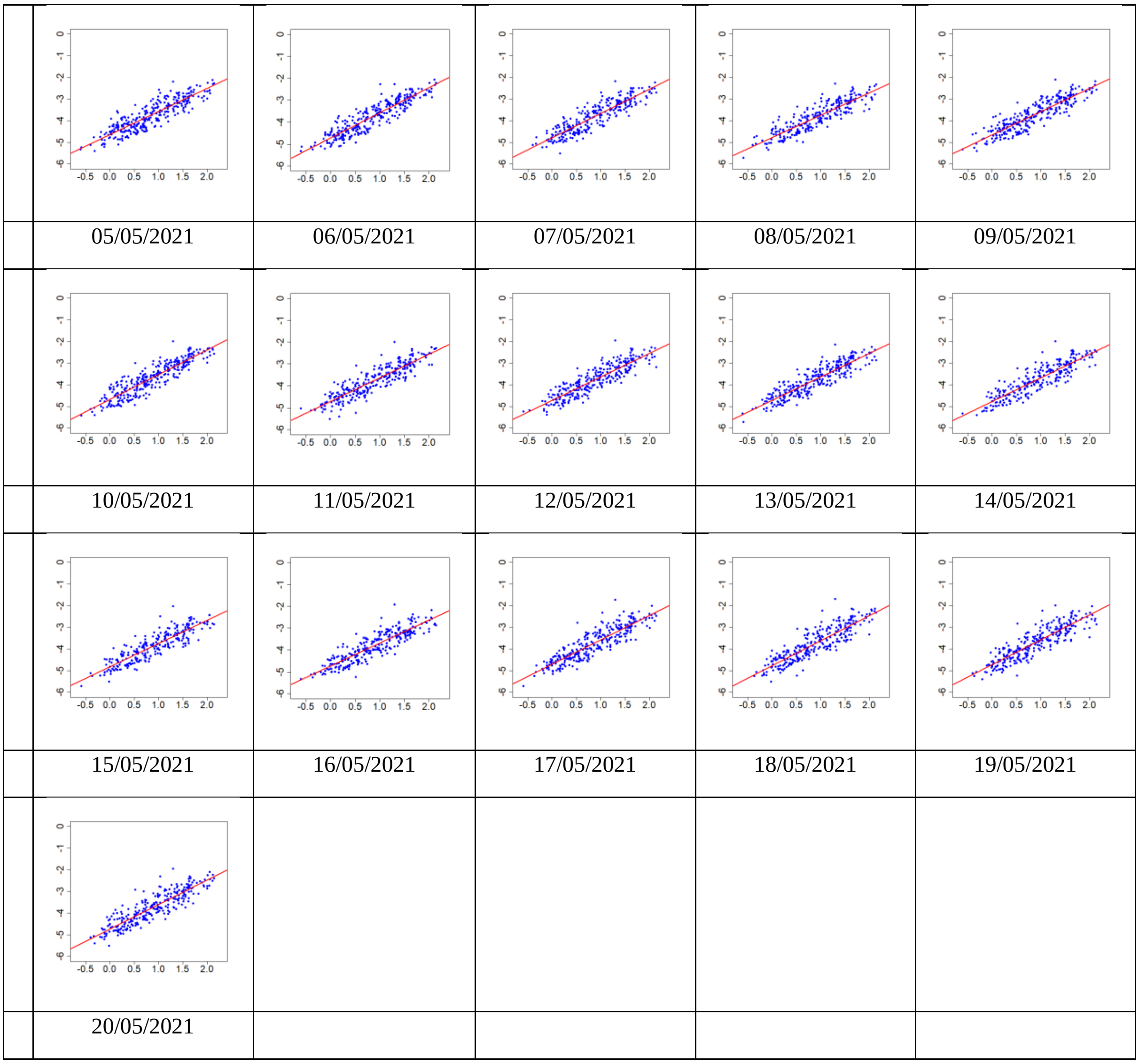

Fig S2. Daily LTLA Density scaling behaviour of COVID-19 cases (i.e. log(Case Density vs. log(Population Density)). The blue dots are the empirical values. A red line represents the single exponent power-law fit.
