# Supplementary material for: Population density and spreading of COVID-19 in England and Wales
Source: PLoS One. 2022 Mar 31;17(3):e0261725. doi: 10.1371/journal.pone.0261725 (PMC8970409; doi:10.1371/journal.pone.0261725)
Supplement: S3 Fig — The blue dots are the empirical values (England). A red line represents the single exponent power-law fit. (PDF) [file pone.0261725.s003.pdf]

<table>
<tr><td></td><td></td><td></td><td></td><td></td><td></td></tr>
<tr><td></td><td>01/03/2020</td><td>02/03/2020</td><td>03/03/2020</td><td>04/03/2020</td><td>05/03/2020</td></tr>
<tr><td></td><td></td><td></td><td></td><td>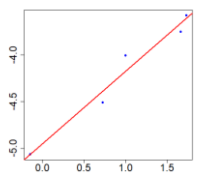
</td><td>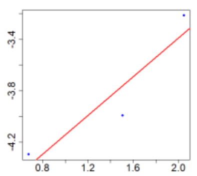
</td></tr>
<tr><td></td><td>06/03/2020</td><td>07/03/2020</td><td>08/03/2020</td><td>09/03/2020</td><td>10/03/2020</td></tr>
</table>

| | | | | | |
|---|---|---|---|---|---|
| | 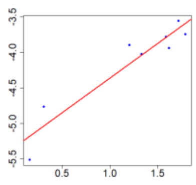 | 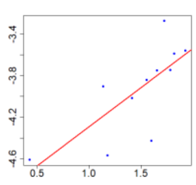 | 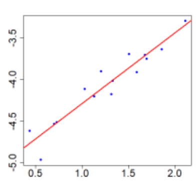 | 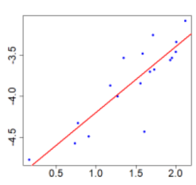 | 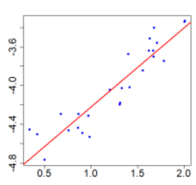 |
| | 11/03/2020 | 12/03/2020 | 13/03/2020 | 14/03/2020 | 15/03/2020 |
| | 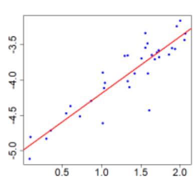 | 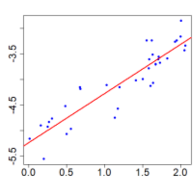 | 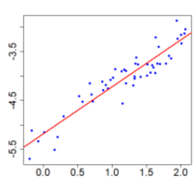 | 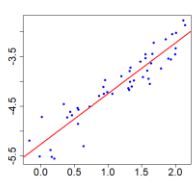 | 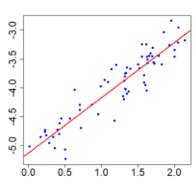 |
| | 16/03/2020 | 17/03/2020 | 18/03/2020 | 19/03/2020 | 20/03/2020 |
| | 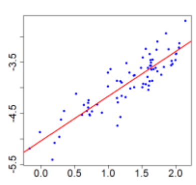 | 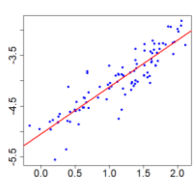 | 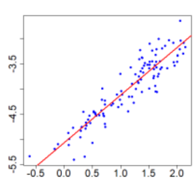 | 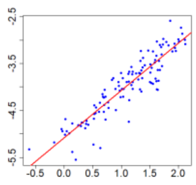 | 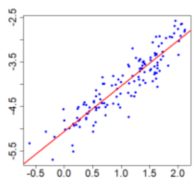 |
| | 21/03/2020 | 22/03/2020 | 23/03/2020 | 24/03/2020 | 25/03/2020 |
| | 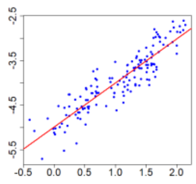 | 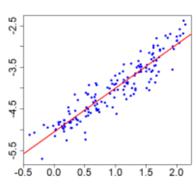 | 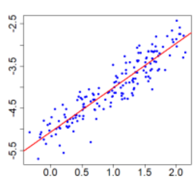 | 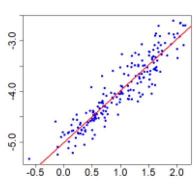 | 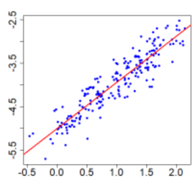 |
| | 26/03/2020 | 27/03/2020 | 28/03/2020 | 29/03/2020 | 30/03/2020 |
| | 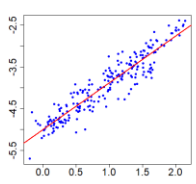 | 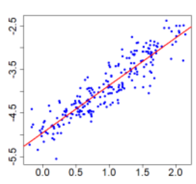 | 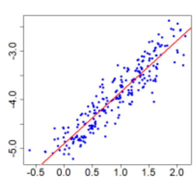 | 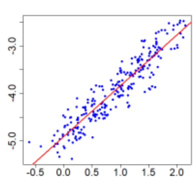 | 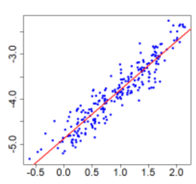 |
| | 31/03/2020 | 01/04/2020 | 02/04/2020 | 03/04/2020 | 04/04/2020 |
| | 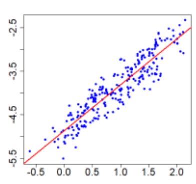 | 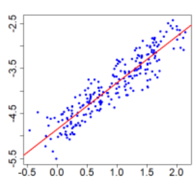 | 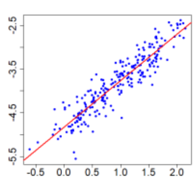 | 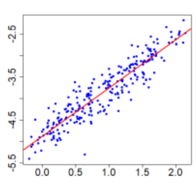 | 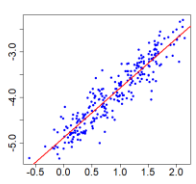 |
| | 05/04/2020 | 06/04/2020 | 07/04/2020 | 08/04/2020 | 09/04/2020 |

| | | | | | |
|---|---|---|---|---|---|
| | 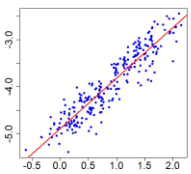 | 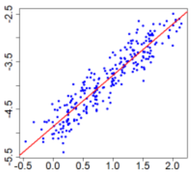 | 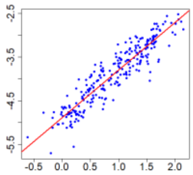 | 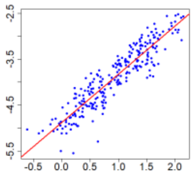 | 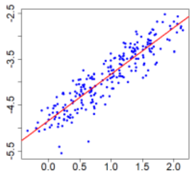 |
| | 10/04/2020 | 11/04/2020 | 12/04/2020 | 13/04/2020 | 14/04/2020 |
| | 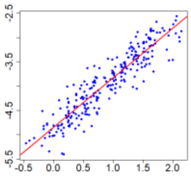 | 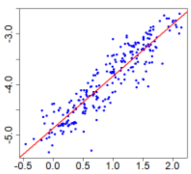 | 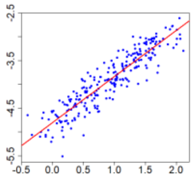 | 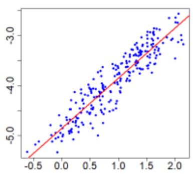 | 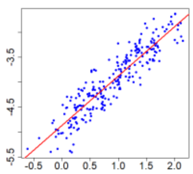 |
| | 15/04/2020 | 16/04/2020 | 17/04/2020 | 18/04/2020 | 19/04/2020 |
| | 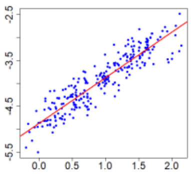 | 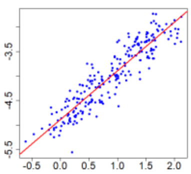 | 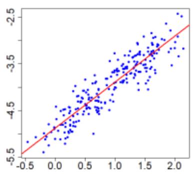 | 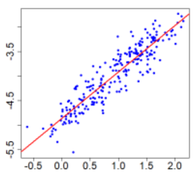 | 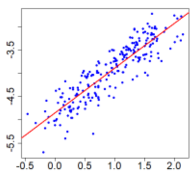 |
| | 20/04/2020 | 21/04/2020 | 22/04/2020 | 23/04/2020 | 24/04/2020 |
| | 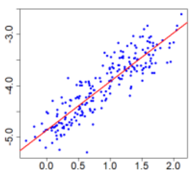 | 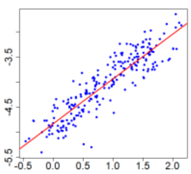 | 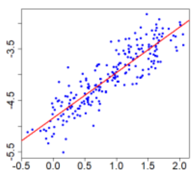 | 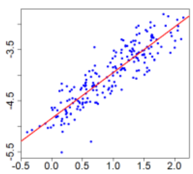 | 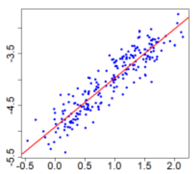 |
| | 25/04/2020 | 26/04/2020 | 27/04/2020 | 28/04/2020 | 29/04/2020 |
| | 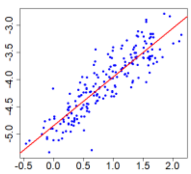 | 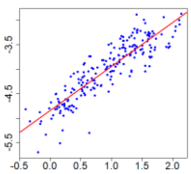 | 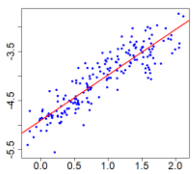 | 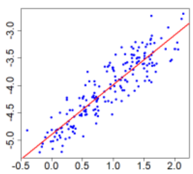 | 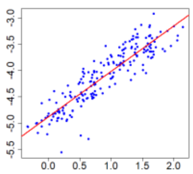 |
| | 30/04/2020 | 01/05/2020 | 02/05/2020 | 03/05/2020 | 04/05/2020 |
| | 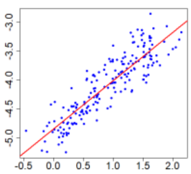 | 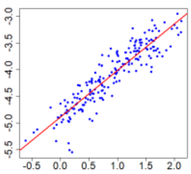 | 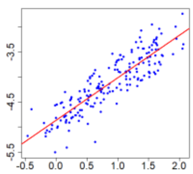 | 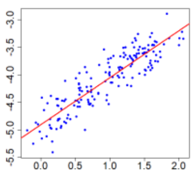 | 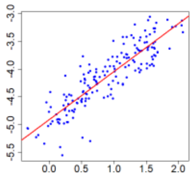 |
| | 05/05/2020 | 06/05/2020 | 07/05/2020 | 08/05/2020 | 09/05/2020 |

| | | | | | |
|---|---|---|---|---|---|
| | 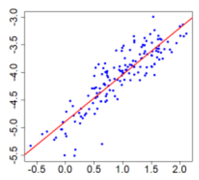 | 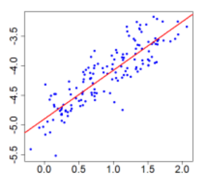 | 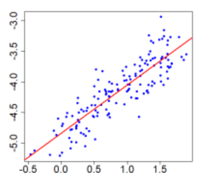 | 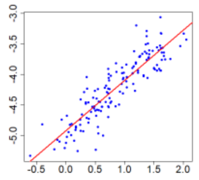 | 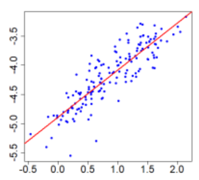 |
| | 10/05/2020 | 11/05/2020 | 12/05/2020 | 13/05/2020 | 14/05/2020 |
| | 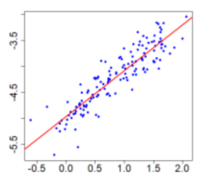 | 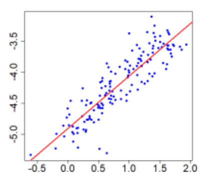 | 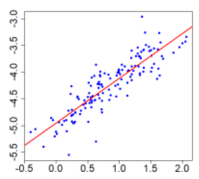 | 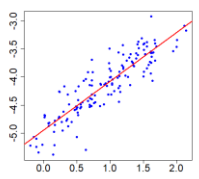 | 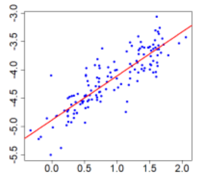 |
| | 15/05/2020 | 16/05/2020 | 17/05/2020 | 18/05/2020 | 19/05/2020 |
| | 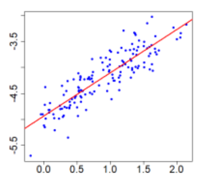 | 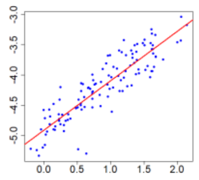 | 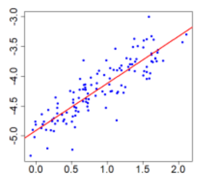 | 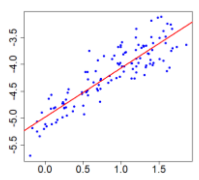 | 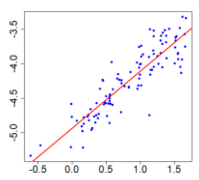 |
| | 20/05/2020 | 21/05/2020 | 22/05/2020 | 23/05/2020 | 24/05/2020 |
| | 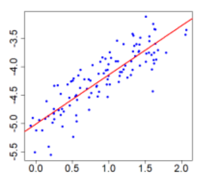 | 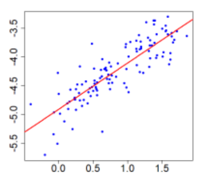 | 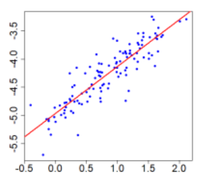 | 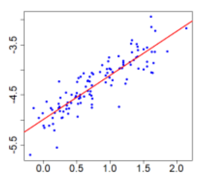 | 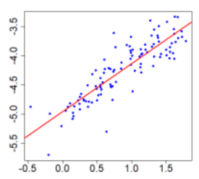 |
| | 25/05/2020 | 26/05/2020 | 27/05/2020 | 28/05/2020 | 29/05/2020 |
| | 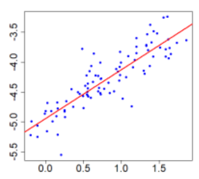 | 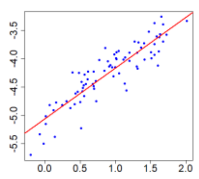 | 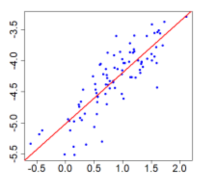 | 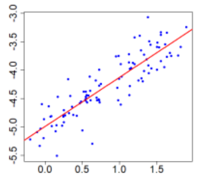 | 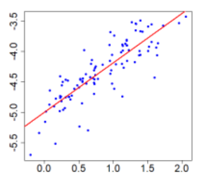 |
| | 30/05/2020 | 31/05/2020 | 01/06/2020 | 02/06/2020 | 03/06/2020 |
| | 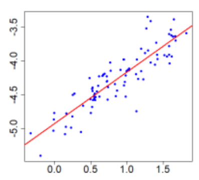 | 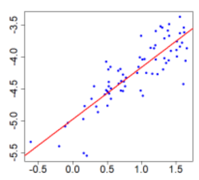 | 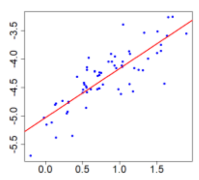 | 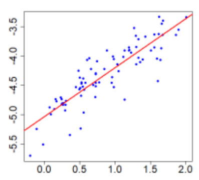 | 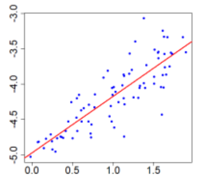 |
| | 04/06/2020 | 05/06/2020 | 06/06/2020 | 07/06/2020 | 08/06/2020 |

| | | | | | |
|---|---|---|---|---|---|
| | 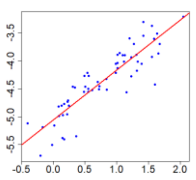 | 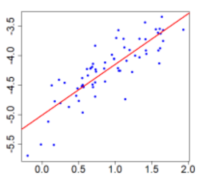 | 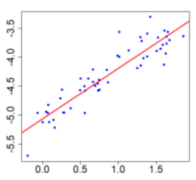 | 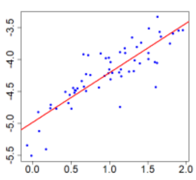 | 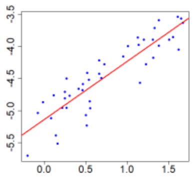 |
| | 09/06/2020 | 10/06/2020 | 11/06/2020 | 12/06/2020 | 13/06/2020 |
| | 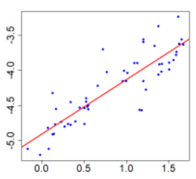 | 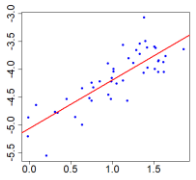 | 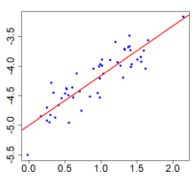 | 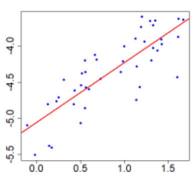 | 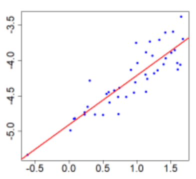 |
| | 14/06/2020 | 15/06/2020 | 16/06/2020 | 17/06/2020 | 18/06/2020 |
| | 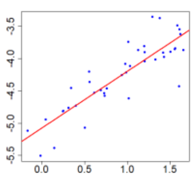 | 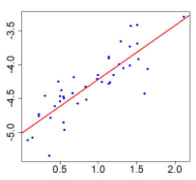 | 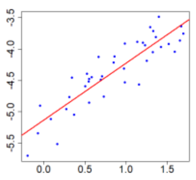 | 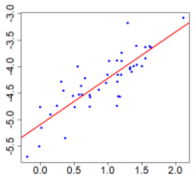 | 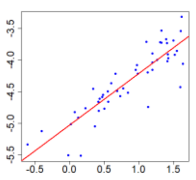 |
| | 19/06/2020 | 20/06/2020 | 21/06/2020 | 22/06/2020 | 23/06/2020 |
| | 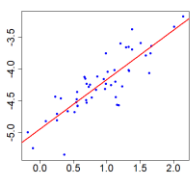 | 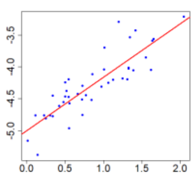 | 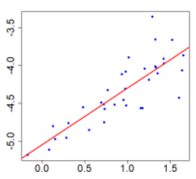 | 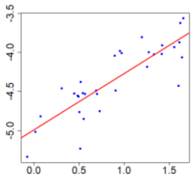 | 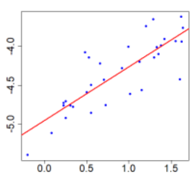 |
| | 24/06/2020 | 25/06/2020 | 26/06/2020 | 27/06/2020 | 28/06/2020 |
| | 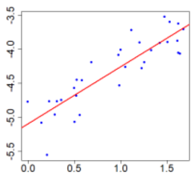 | 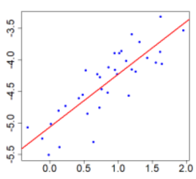 | 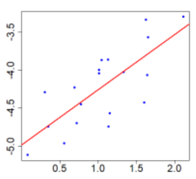 | 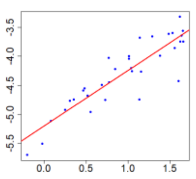 | 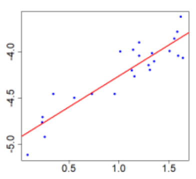 |
| | 29/06/2020 | 30/06/2020 | 01/07/2020 | 02/07/2020 | 03/07/2020 |
| | 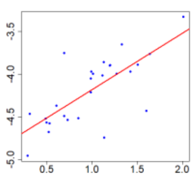 | 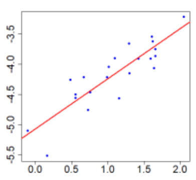 | 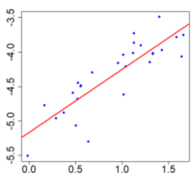 | 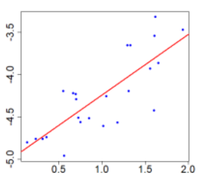 | 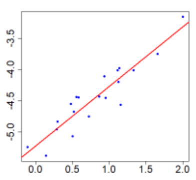 |
| | 04/07/2020 | 05/07/2020 | 06/07/2020 | 07/07/2020 | 08/07/2020 |

| | | | | | |
|---|---|---|---|---|---|
| | 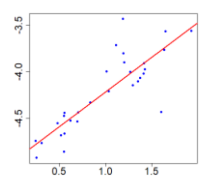 | 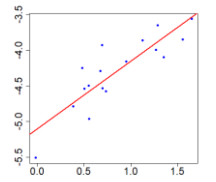 | 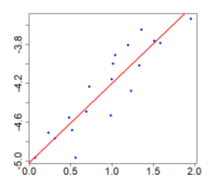 | 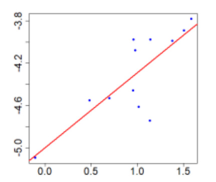 | 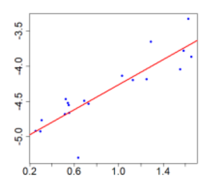 |
| | 09/07/2020 | 10/07/2020 | 11/07/2020 | 12/07/2020 | 13/07/2020 |
| | 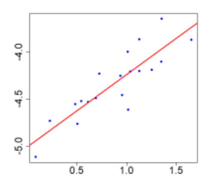 | 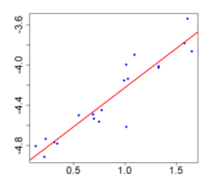 | 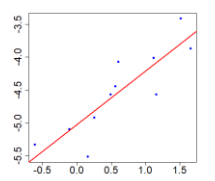 | 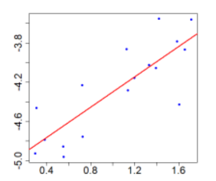 | 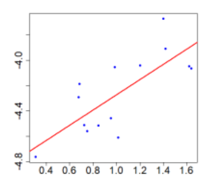 |
| | 14/07/2020 | 15/07/2020 | 16/07/2020 | 17/07/2020 | 18/07/2020 |
| | 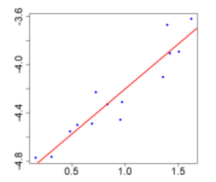 | 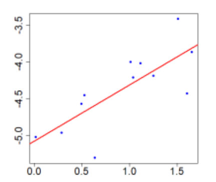 | 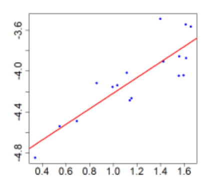 | 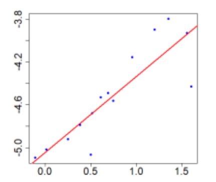 | 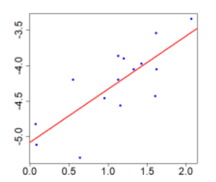 |
| | 19/07/2020 | 20/07/2020 | 21/07/2020 | 22/07/2020 | 23/07/2020 |
| | 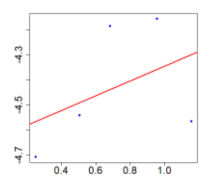 | 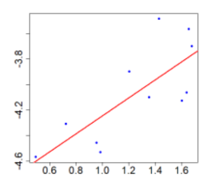 | 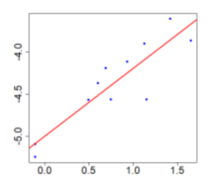 | 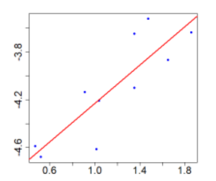 | 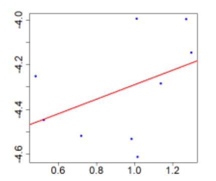 |
| | 24/07/2020 | 25/07/2020 | 26/07/2020 | 27/07/2020 | 28/07/2020 |
| | 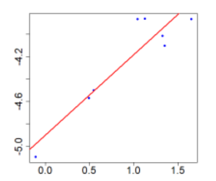 | 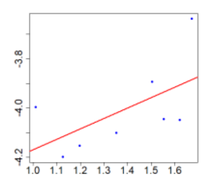 | 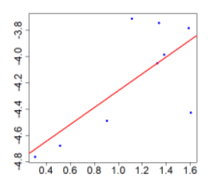 | 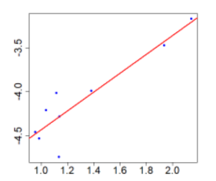 | 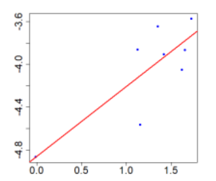 |
| | 29/07/2020 | 30/07/2020 | 31/07/2020 | 01/08/2020 | 02/08/2020 |
| | 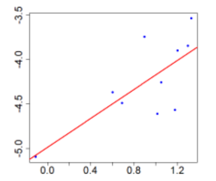 | 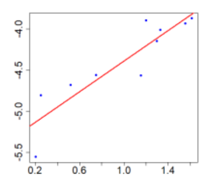 | 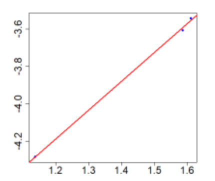 | 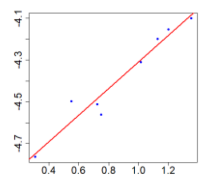 | 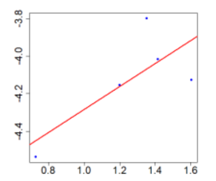 |
| | 03/08/2020 | 04/08/2020 | 05/08/2020 | 06/08/2020 | 07/08/2020 |

| | | | | | |
|---|---|---|---|---|---|
| | 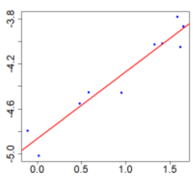 | 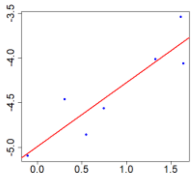 | 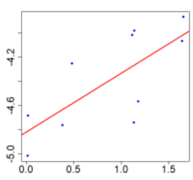 | 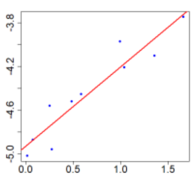 | 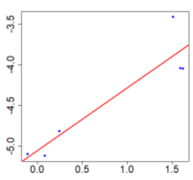 |
| | 08/08/2020 | 09/08/2020 | 10/08/2020 | 11/08/2020 | 12/08/2020 |
| | 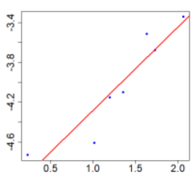 | 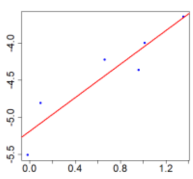 | 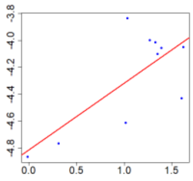 | 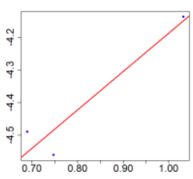 | 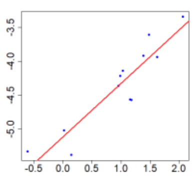 |
| | 13/08/2020 | 14/08/2020 | 15/08/2020 | 16/08/2020 | 17/08/2020 |
| | 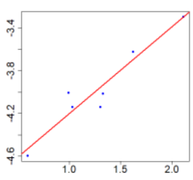 | | 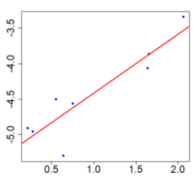 | 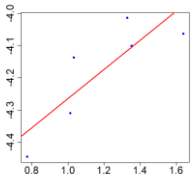 | 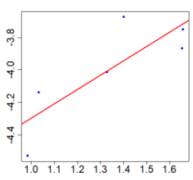 |
| | 18/08/2020 | 19/08/2020 | 20/08/2020 | 21/08/2020 | 22/08/2020 |
| | 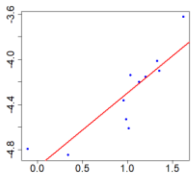 | 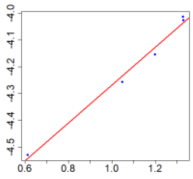 | 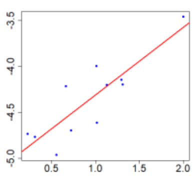 | 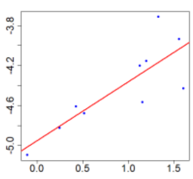 | 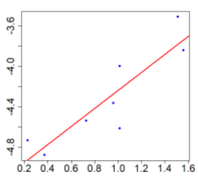 |
| | 23/08/2020 | 24/08/2020 | 25/08/2020 | 26/08/2020 | 27/08/2020 |
| | 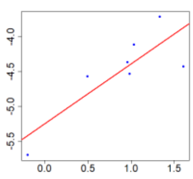 | 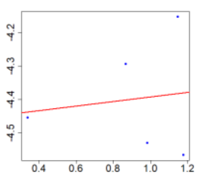 | 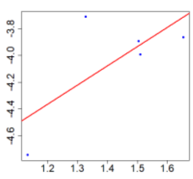 | 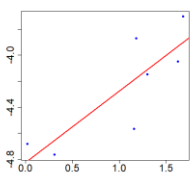 | 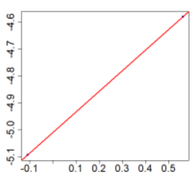 |
| | 28/08/2020 | 29/08/2020 | 30/08/2020 | 31/08/2020 | 01/09/2020 |
| | 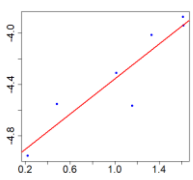 | 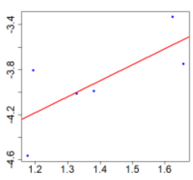 | 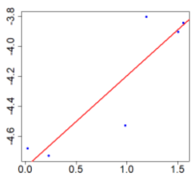 | 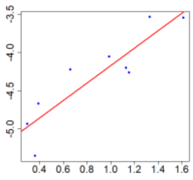 | 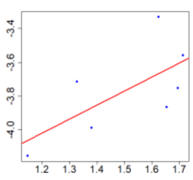 |
| | 02/09/2020 | 03/09/2020 | 04/09/2020 | 05/09/2020 | 06/09/2020 |

| | | | | | |
|---|---|---|---|---|---|
| | 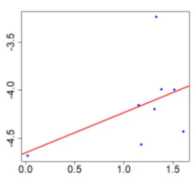 | 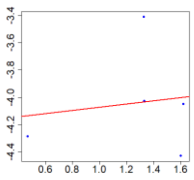 | 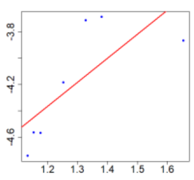 | 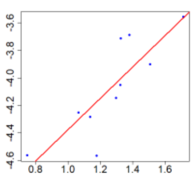 | 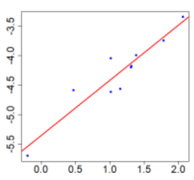 |
| | 07/09/2020 | 08/09/2020 | 09/09/2020 | 10/09/2020 | 11/09/2020 |
| | 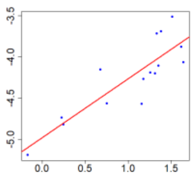 | 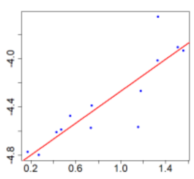 | 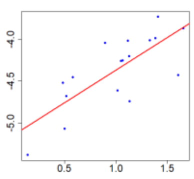 | 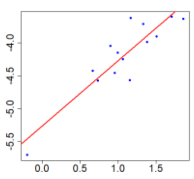 | 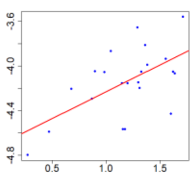 |
| | 12/09/2020 | 13/09/2020 | 14/09/2020 | 15/09/2020 | 16/09/2020 |
| | 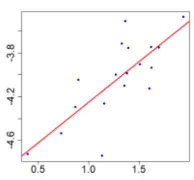 | 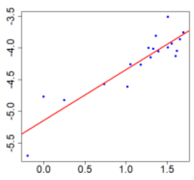 | 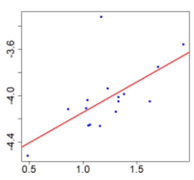 | 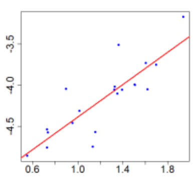 | 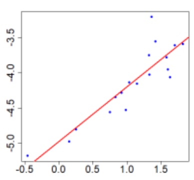 |
| | 17/09/2020 | 18/09/2020 | 19/09/2020 | 20/09/2020 | 21/09/2020 |
| | 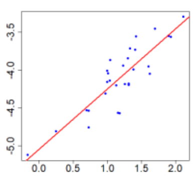 | 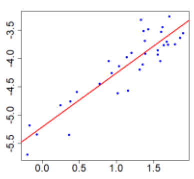 | 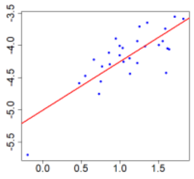 | 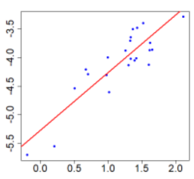 | 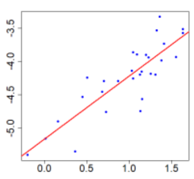 |
| | 22/09/2020 | 23/09/2020 | 24/09/2020 | 25/09/2020 | 26/09/2020 |
| | 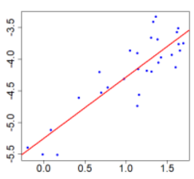 | 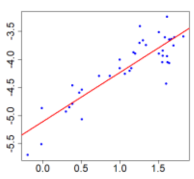 | 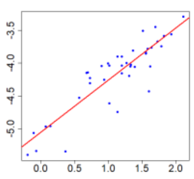 | 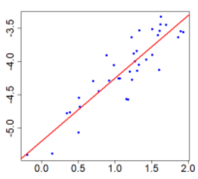 | 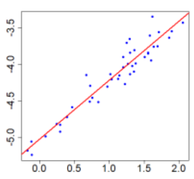 |
| | 27/09/2020 | 28/09/2020 | 29/09/2020 | 30/09/2020 | 01/10/2020 |
| | 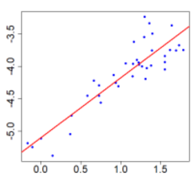 | 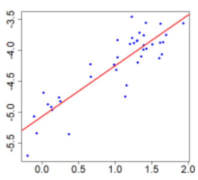 | 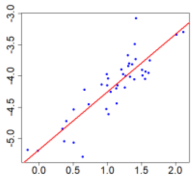 | 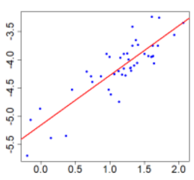 | 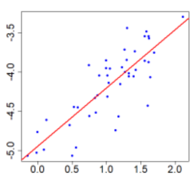 |
| | 02/10/2020 | 03/10/2020 | 04/10/2020 | 05/10/2020 | 06/10/2020 |

| | | | | | |
|---|---|---|---|---|---|
| | 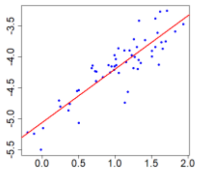 | 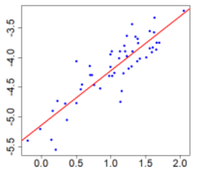 | 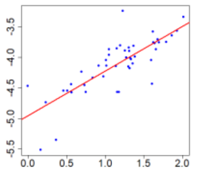 | 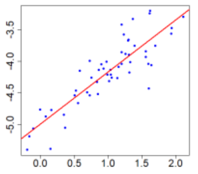 | 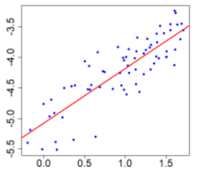 |
| | 07/10/2020 | 08/10/2020 | 09/10/2020 | 10/10/2020 | 11/10/2020 |
| | 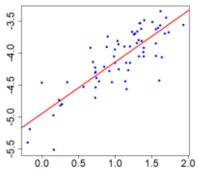 | 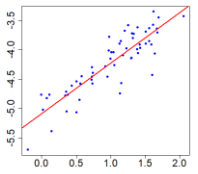 | 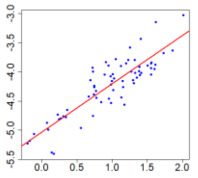 | 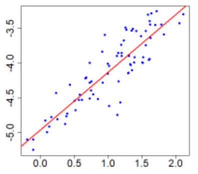 | 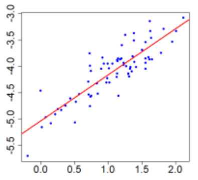 |
| | 12/10/2020 | 13/10/2020 | 14/10/2020 | 15/10/2020 | 16/10/2020 |
| | 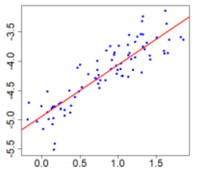 | 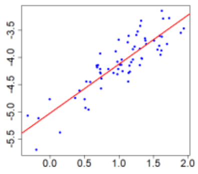 | 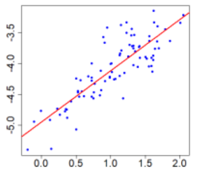 | 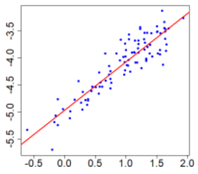 | 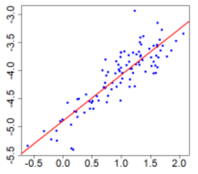 |
| | 17/10/2020 | 18/10/2020 | 19/10/2020 | 20/10/2020 | 21/10/2020 |
| | 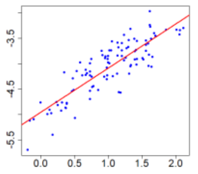 | 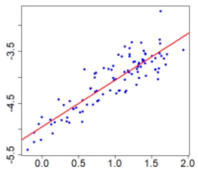 | 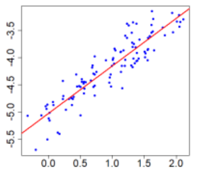 | 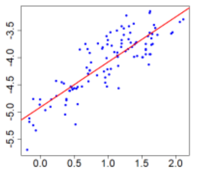 | 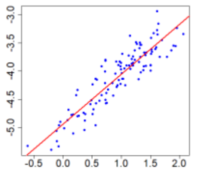 |
| | 22/10/2020 | 23/10/2020 | 24/10/2020 | 25/10/2020 | 26/10/2020 |
| | 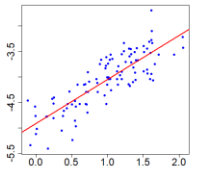 | 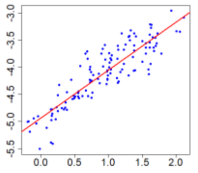 | 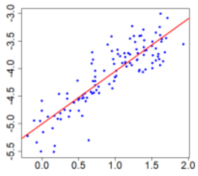 | 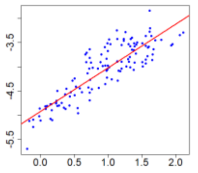 | 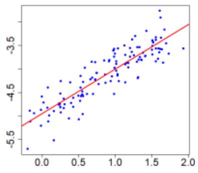 |
| | 27/10/2020 | 28/10/2020 | 29/10/2020 | 30/10/2020 | 31/10/2020 |
| | 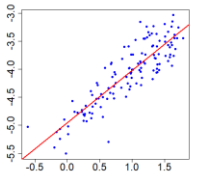 | 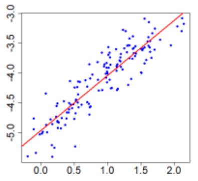 | 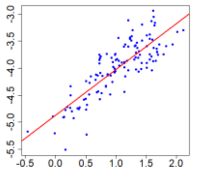 | 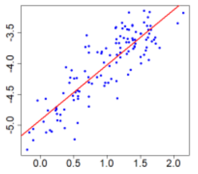 | 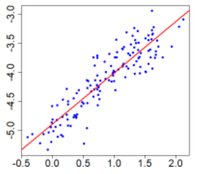 |
| | 01/11/2020 | 02/11/2020 | 03/11/2020 | 04/11/2020 | 05/11/2020 |

| | | | | | |
|---|---|---|---|---|---|
| | 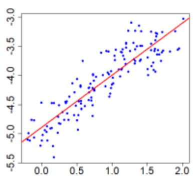 | 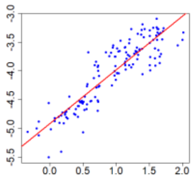 | 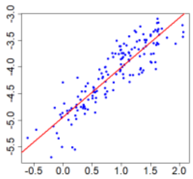 | 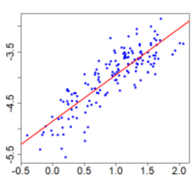 | 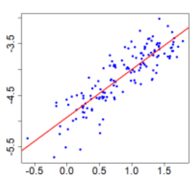 |
| | 06/11/2020 | 07/11/2020 | 08/11/2020 | 09/11/2020 | 10/11/2020 |
| | 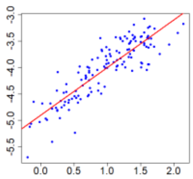 | 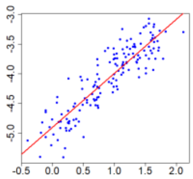 | 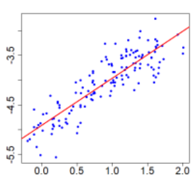 | 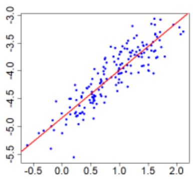 | 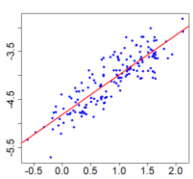 |
| | 11/11/2020 | 12/11/2020 | 13/11/2020 | 14/11/2020 | 15/11/2020 |
| | 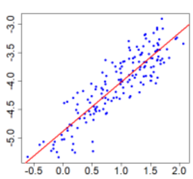 | 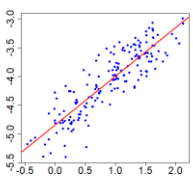 | 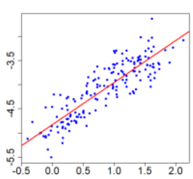 | 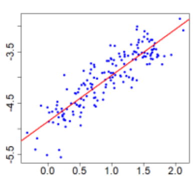 | 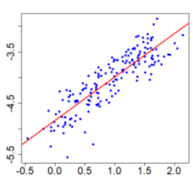 |
| | 16/11/2020 | 17/11/2020 | 18/11/2020 | 19/11/2020 | 20/11/2020 |
| | 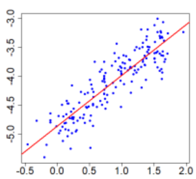 | 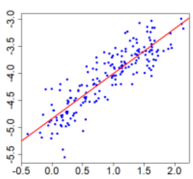 | 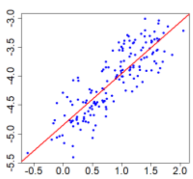 | 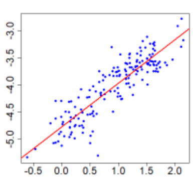 | 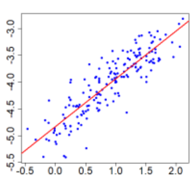 |
| | 21/11/2020 | 22/11/2020 | 23/11/2020 | 24/11/2020 | 25/11/2020 |
| | 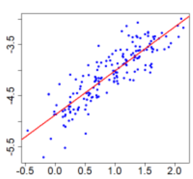 | 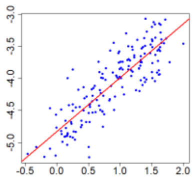 | 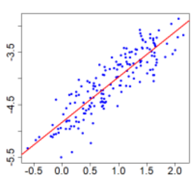 | 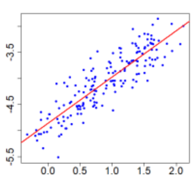 | 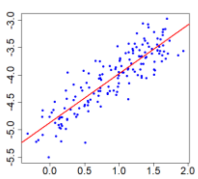 |
| | 26/11/2020 | 27/11/2020 | 28/11/2020 | 29/11/2020 | 30/11/2020 |
| | 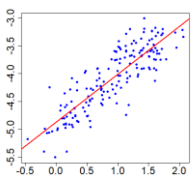 | 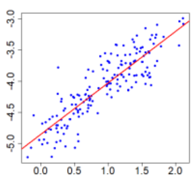 | 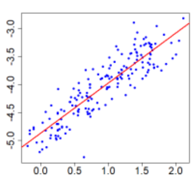 | 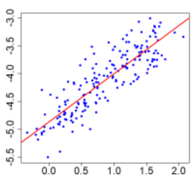 | 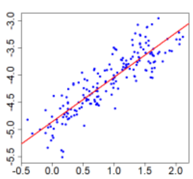 |
| | 01/12/2020 | 02/12/2020 | 03/12/2020 | 04/12/2020 | 05/12/2020 |

| | | | | | |
|---|---|---|---|---|---|
| | 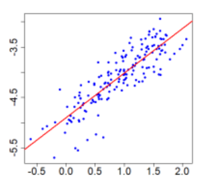 | 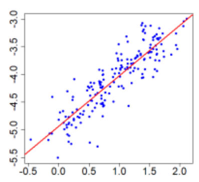 | 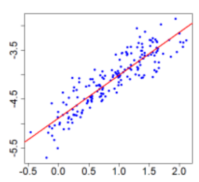 | 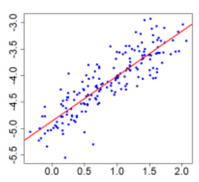 | 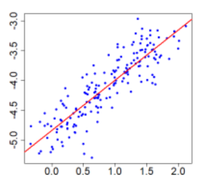 |
| | 06/12/2020 | 07/12/2020 | 08/12/2020 | 09/12/2020 | 10/12/2020 |
| | 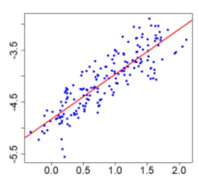 | 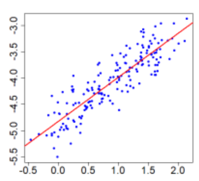 | 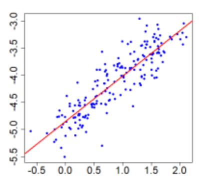 | 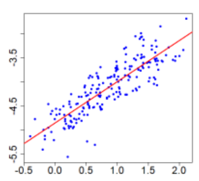 | 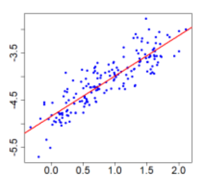 |
| | 11/12/2020 | 12/12/2020 | 13/12/2020 | 14/12/2020 | 15/12/2020 |
| | 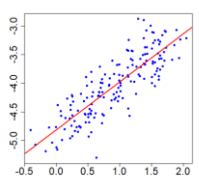 | 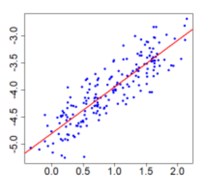 | 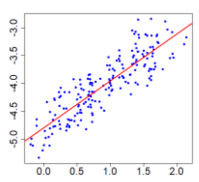 | 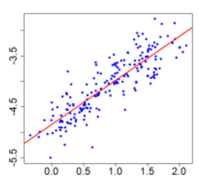 | 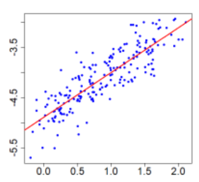 |
| | 16/12/2020 | 17/12/2020 | 18/12/2020 | 19/12/2020 | 20/12/2020 |
| | 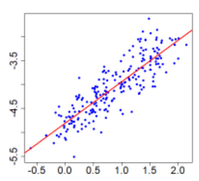 | 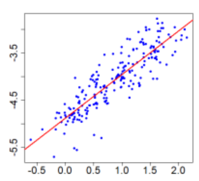 | 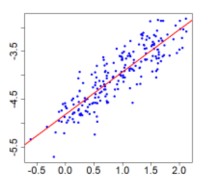 | 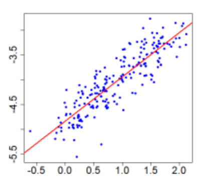 | 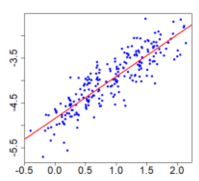 |
| | 21/12/2020 | 22/12/2020 | 23/12/2020 | 24/12/2020 | 25/12/2020 |
| | 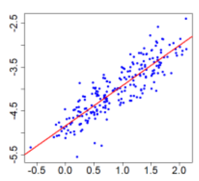 | 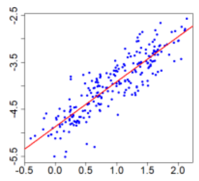 | 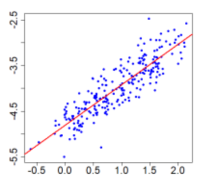 | 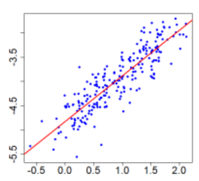 | 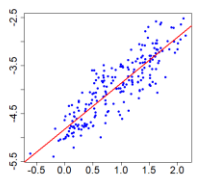 |
| | 26/12/2020 | 27/12/2020 | 28/12/2020 | 29/12/2020 | 30/12/2020 |
| | 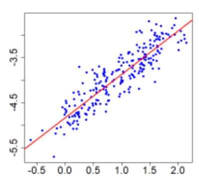 | 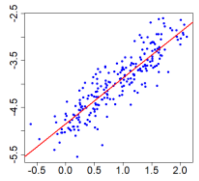 | 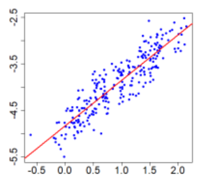 | 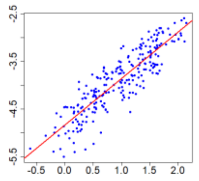 | 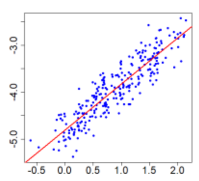 |
| | 31/12/2020 | 01/01/2021 | 02/01/2021 | 03/01/2021 | 04/01/2021 |

| | | | | | |
|---|---|---|---|---|---|
| | 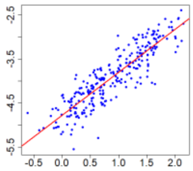 | 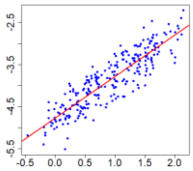 | 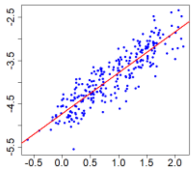 | 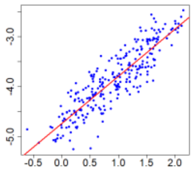 | 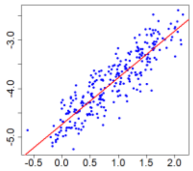 |
| | 05/01/2021 | 06/01/2021 | 07/01/2021 | 08/01/2021 | 09/01/2021 |
| | 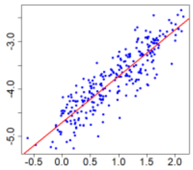 | 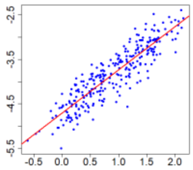 | 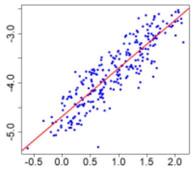 | 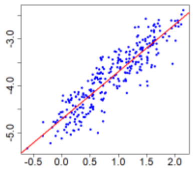 | 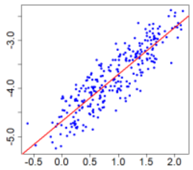 |
| | 10/01/2021 | 11/01/2021 | 12/01/2021 | 13/01/2021 | 14/01/2021 |
| | 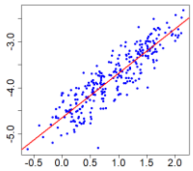 | 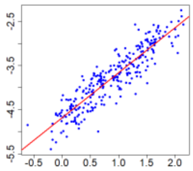 | 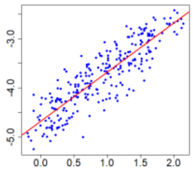 | 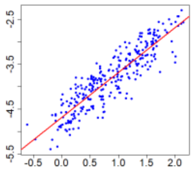 | 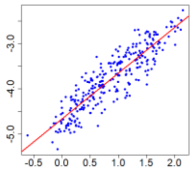 |
| | 15/01/2021 | 16/01/2021 | 17/01/2021 | 18/01/2021 | 19/01/2021 |
| | 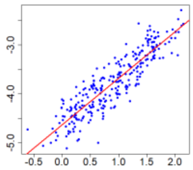 | 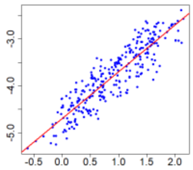 | 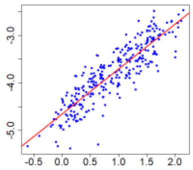 | 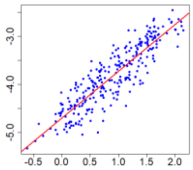 | 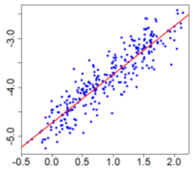 |
| | 20/01/2021 | 21/01/2021 | 22/01/2021 | 23/01/2021 | 24/01/2021 |
| | 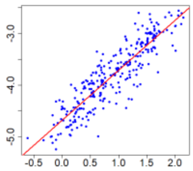 | 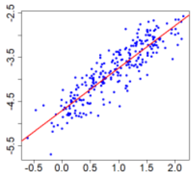 | 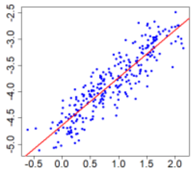 | 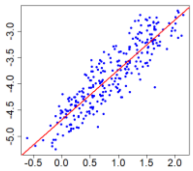 | 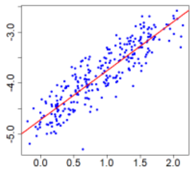 |
| | 25/01/2021 | 26/01/2021 | 27/01/2021 | 28/01/2021 | 29/01/2021 |
| | 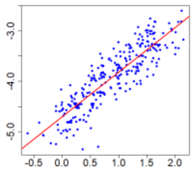 | 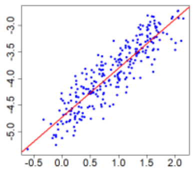 | 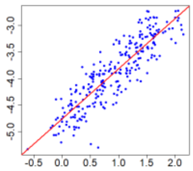 | 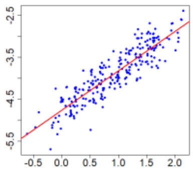 | 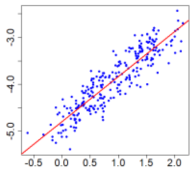 |
| | 30/01/2021 | 31/01/2021 | 01/02/2021 | 02/02/2021 | 03/02/2021 |

| | | | | | |
|---|---|---|---|---|---|
| | 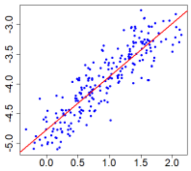 | 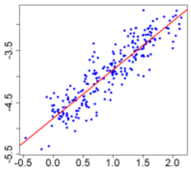 | 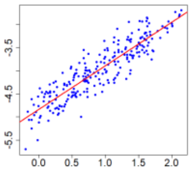 | 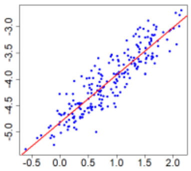 | 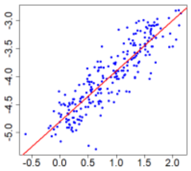 |
| | 04/02/2021 | 05/02/2021 | 06/02/2021 | 07/02/2021 | 08/02/2021 |
| | 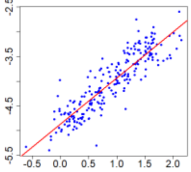 | 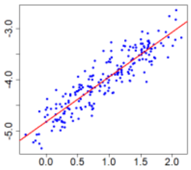 | 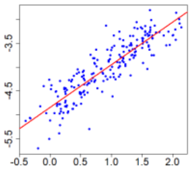 | 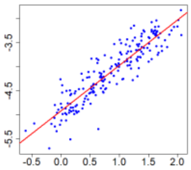 | 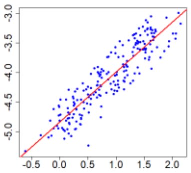 |
| | 09/02/2021 | 10/02/2021 | 11/02/2021 | 12/02/2021 | 13/02/2021 |
| | 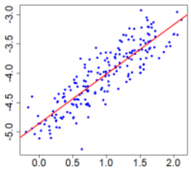 | 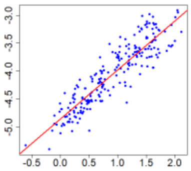 | 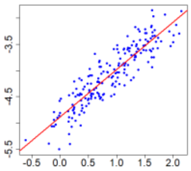 | 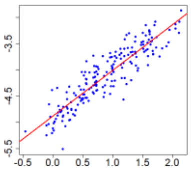 | 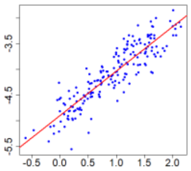 |
| | 14/02/2021 | 15/02/2021 | 16/02/2021 | 17/02/2021 | 18/02/2021 |
| | 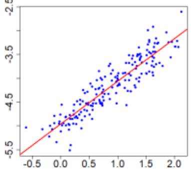 | 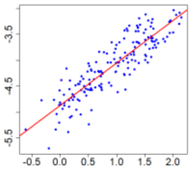 | 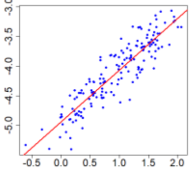 | 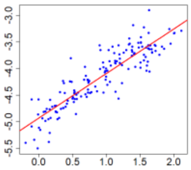 | 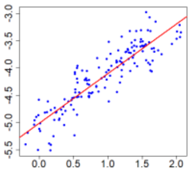 |
| | 19/02/2021 | 20/02/2021 | 21/02/2021 | 22/02/2021 | 23/02/2021 |
| | 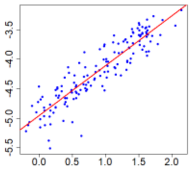 | 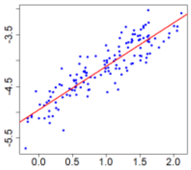 | 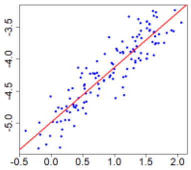 | 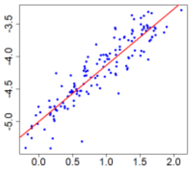 | 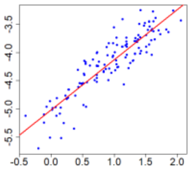 |
| | 24/02/2021 | 25/02/2021 | 26/02/2021 | 27/02/2021 | 28/02/2021 |
| | 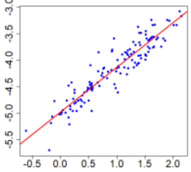 | 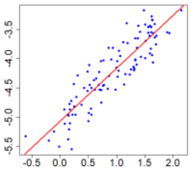 | 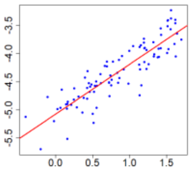 | 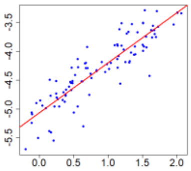 | 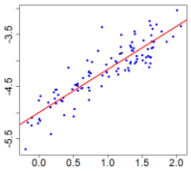 |
| | 01/03/2021 | 02/03/2021 | 03/03/2021 | 04/03/2021 | 05/03/2021 |

| | | | | | |
|---|---|---|---|---|---|
| | 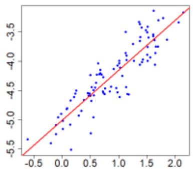 | 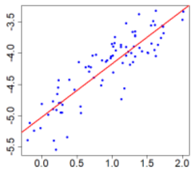 | 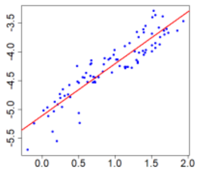 | 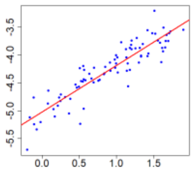 | 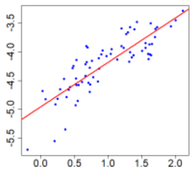 |
| | 06/03/2021 | 07/03/2021 | 08/03/2021 | 09/03/2021 | 10/03/2021 |
| | 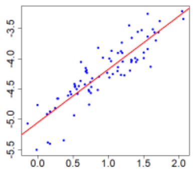 | 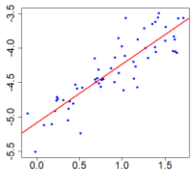 | 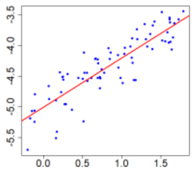 | 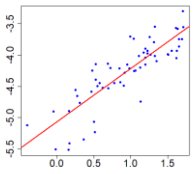 | 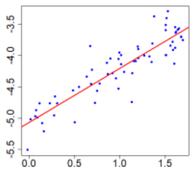 |
| | 11/03/2021 | 12/03/2021 | 13/03/2021 | 14/03/2021 | 15/03/2021 |
| | 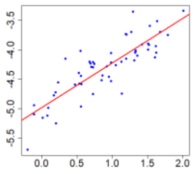 | 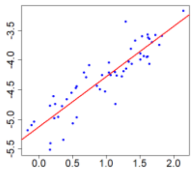 | 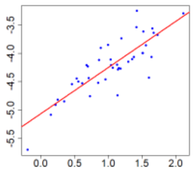 | 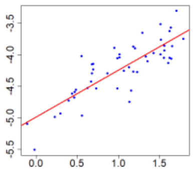 | 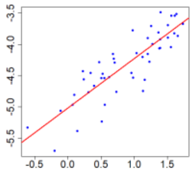 |
| | 16/03/2021 | 17/03/2021 | 18/03/2021 | 19/03/2021 | 20/03/2021 |
| | 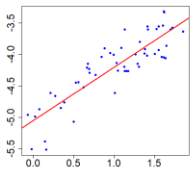 | 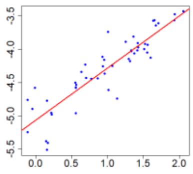 | 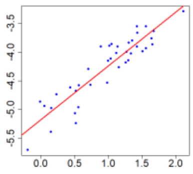 | 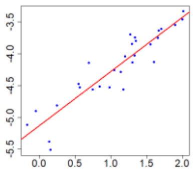 | 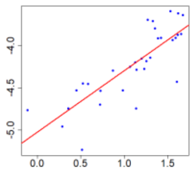 |
| | 21/03/2021 | 22/03/2021 | 23/03/2021 | 24/03/2021 | 25/03/2021 |
| | 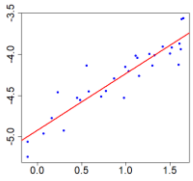 | 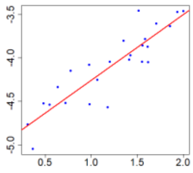 | 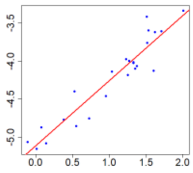 | 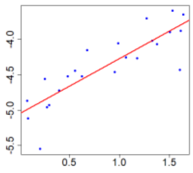 | 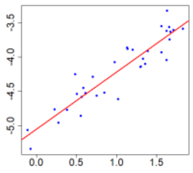 |
| | 26/03/2021 | 27/03/2021 | 28/03/2021 | 29/03/2021 | 30/03/2021 |
| | 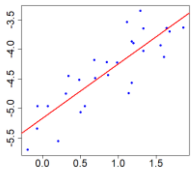 | 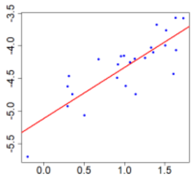 | 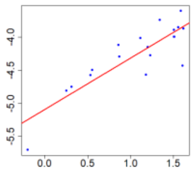 | 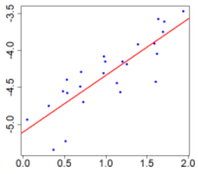 | 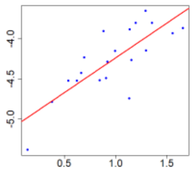 |
| | 31/03/2021 | 01/04/2021 | 02/04/2021 | 03/04/2021 | 04/04/2021 |

| | | | | | |
|---|---|---|---|---|---|
| | 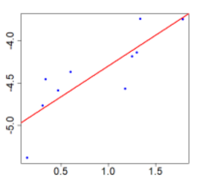 | 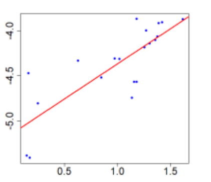 | 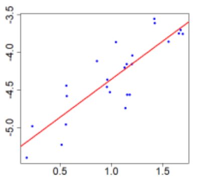 | 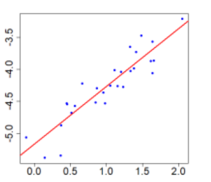 | 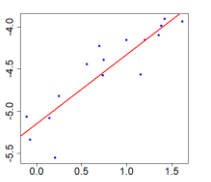 |
| | 05/04/2021 | 06/04/2021 | 07/04/2021 | 08/04/2021 | 09/04/2021 |
| | 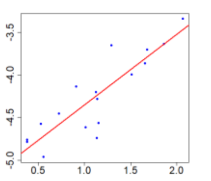 | 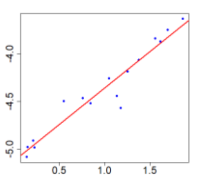 | 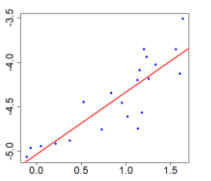 | 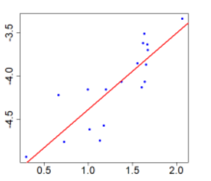 | 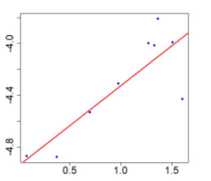 |
| | 10/04/2021 | 11/04/2021 | 12/04/2021 | 13/04/2021 | 14/04/2021 |
| | 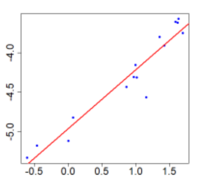 | 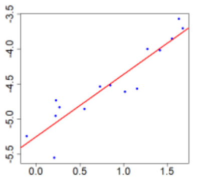 | 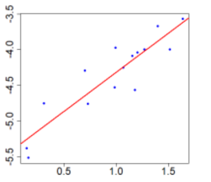 | 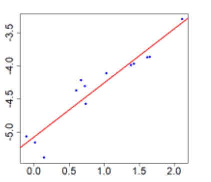 | 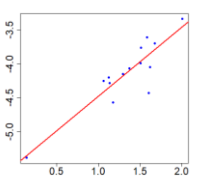 |
| | 15/04/2021 | 16/04/2021 | 17/04/2021 | 18/04/2021 | 19/04/2021 |
| | 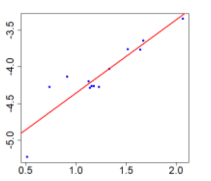 | 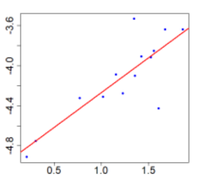 | 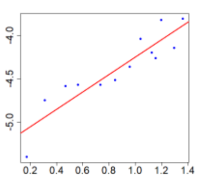 | 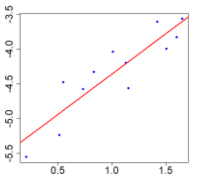 | 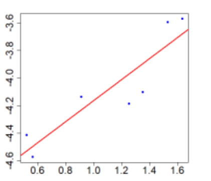 |
| | 20/04/2021 | 21/04/2021 | 22/04/2021 | 23/04/2021 | 24/04/2021 |
| | 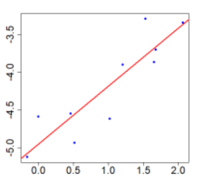 | 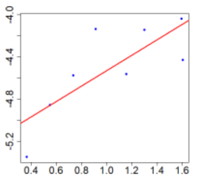 | 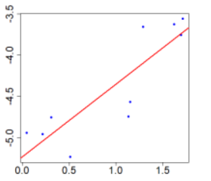 | 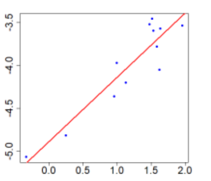 | 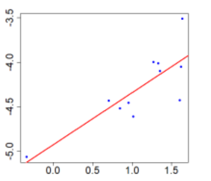 |
| | 25/04/2021 | 26/04/2021 | 27/04/2021 | 28/04/2021 | 29/04/2021 |
| | 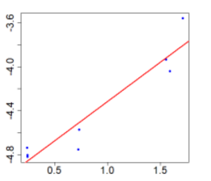 | 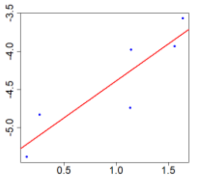 | 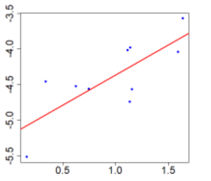 | 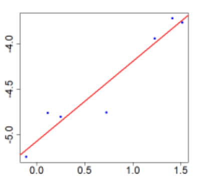 | 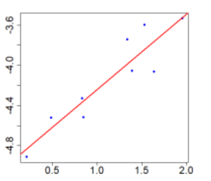 |
| | 30/04/2021 | 01/05/2021 | 02/05/2021 | 03/05/2021 | 04/05/2021 |

| | | | | | |
|---|---|---|---|---|---|
| | 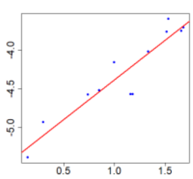 | 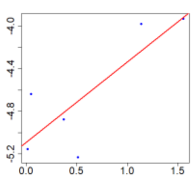 | 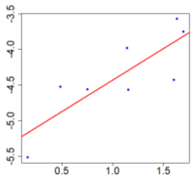 | 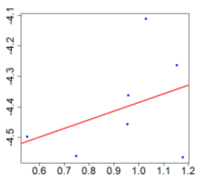 | 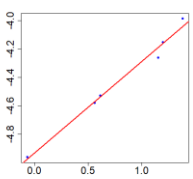 |
| | 05/05/2021 | 06/05/2021 | 07/05/2021 | 08/05/2021 | 09/05/2021 |
| | 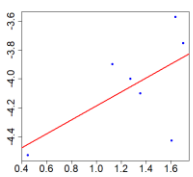 | 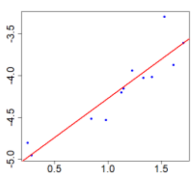 | 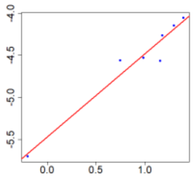 | 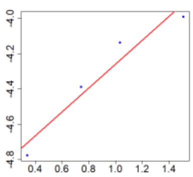 | |
| | 10/05/2021 | 11/05/2021 | 12/05/2021 | 13/05/2021 | 14/05/2021 |
| | 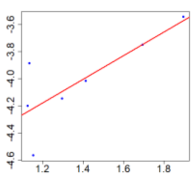 | 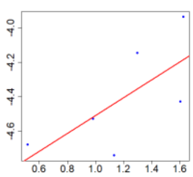 | 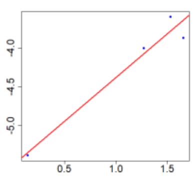 | 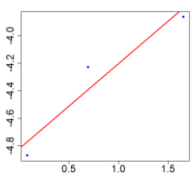 | 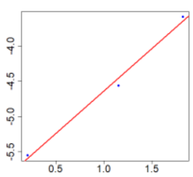 |
| | 15/05/2021 | 16/05/2021 | 17/05/2021 | 18/05/2021 | 19/05/2021 |
| | 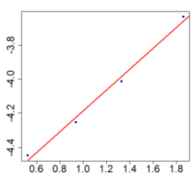 | | | | |
| | 20/05/2021 | | | | |

**Fig S3. Daily LTLA Density scaling plots of COVID-19 death (i.e. log(Death Density vs. log(Population Density)).** The blue dots are the empirical values (England). A red line represents the single exponent power-law fit.
