# Supplementary material for: Population density and spreading of COVID-19 in England and Wales
Source: PLoS One. 2022 Mar 31;17(3):e0261725. doi: 10.1371/journal.pone.0261725 (PMC8970409; doi:10.1371/journal.pone.0261725)
Supplement: S5 Fig — Regions that are red are above expectation and blue is below. The darker the shade the further from the scaling law. The geoplots contain public sector information licensed under the Open Government Licence v3.0. (PDF) [file pone.0261725.s005.pdf]

	01/03/2020	02/03/2020	03/03/2020	04/03/2020	05/03/2020
				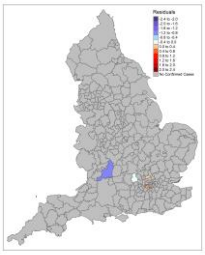	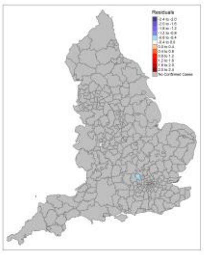
	06/03/2020	07/03/2020	08/03/2020	09/03/2020	10/03/2020
	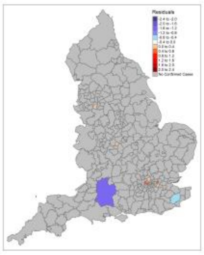	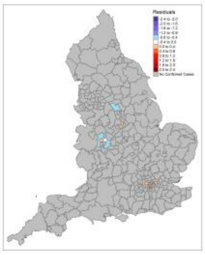	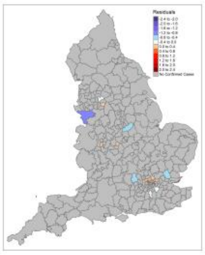	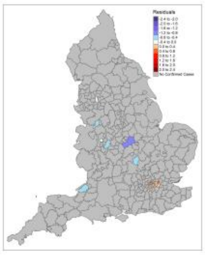	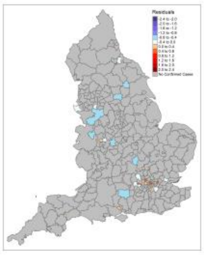
	11/03/2020	12/03/2020	13/03/2020	14/03/2020	15/03/2020
	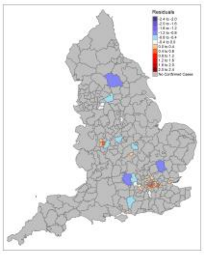	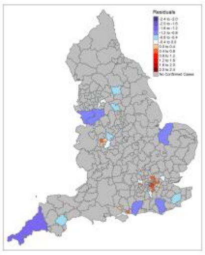	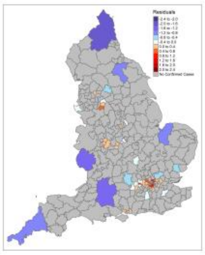	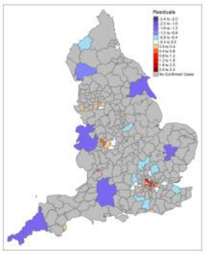	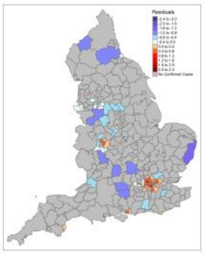
	16/03/2020	17/03/2020	18/03/2020	19/03/2020	20/03/2020
	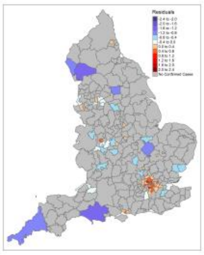	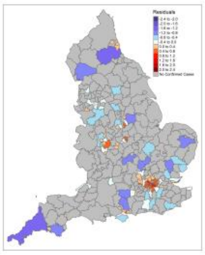	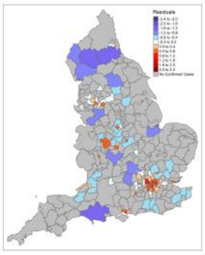	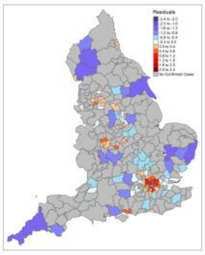	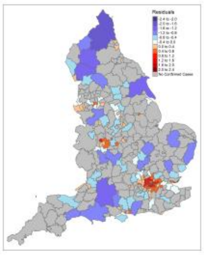
	21/03/2020	22/03/2020	23/03/2020	24/03/2020	25/03/2020
	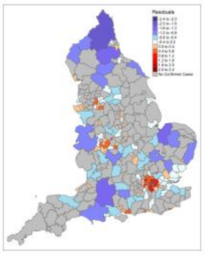	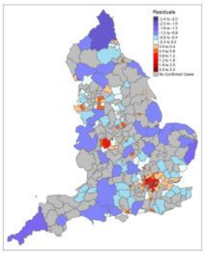	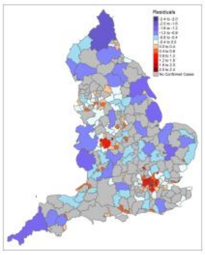	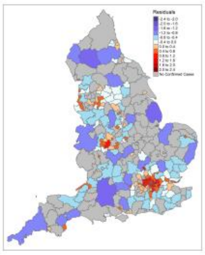	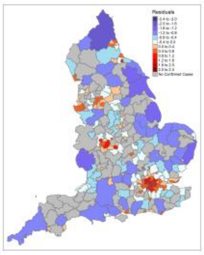
	26/03/2020	27/03/2020	28/03/2020	29/03/2020	30/03/2020

	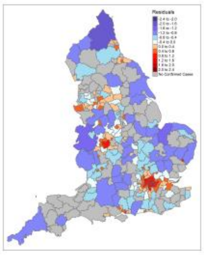	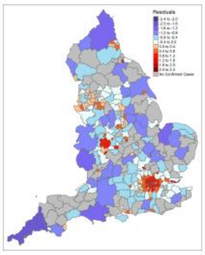	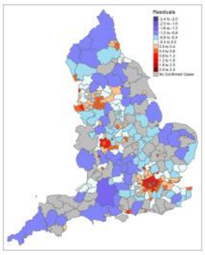	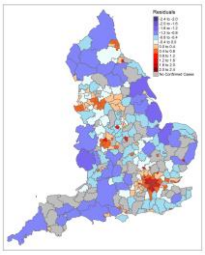	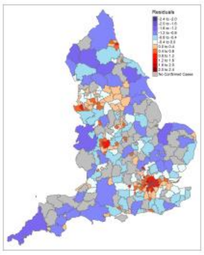
	31/03/2020	01/04/2020	02/04/2020	03/04/2020	04/04/2020
	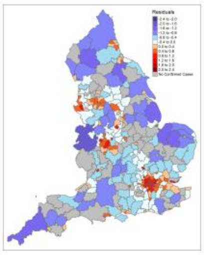	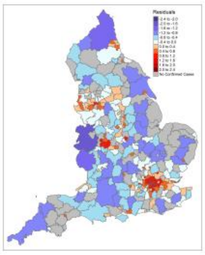	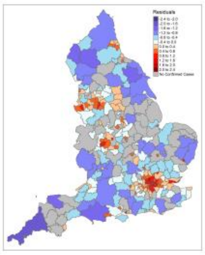	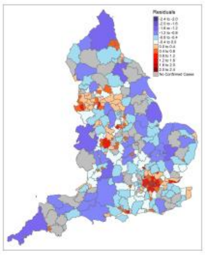	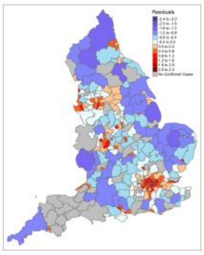
	05/04/2020	06/04/2020	07/04/2020	08/04/2020	09/04/2020
	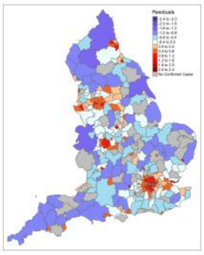	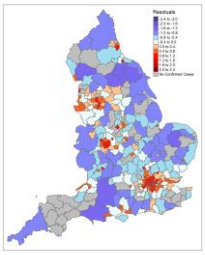	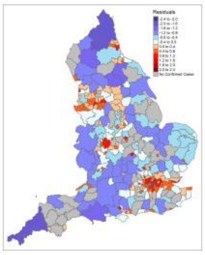	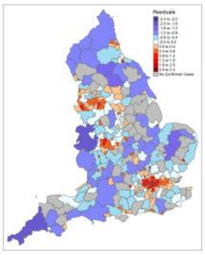	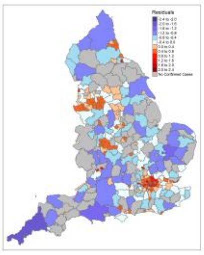
	10/04/2020	11/04/2020	12/04/2020	13/04/2020	14/04/2020
	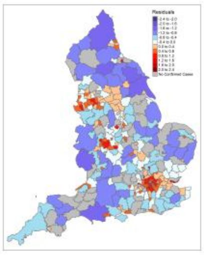	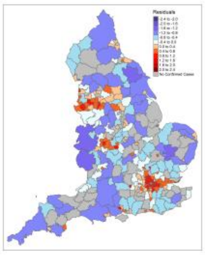	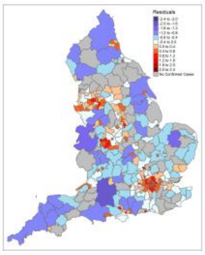	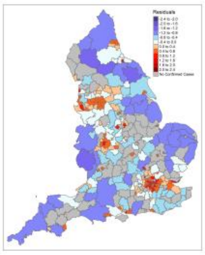	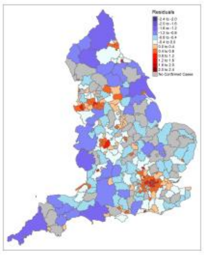
	15/04/2020	16/04/2020	17/04/2020	18/04/2020	19/04/2020
	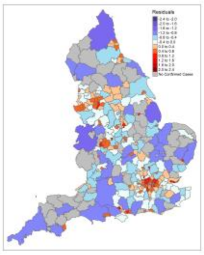	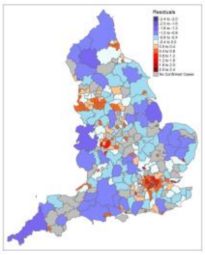	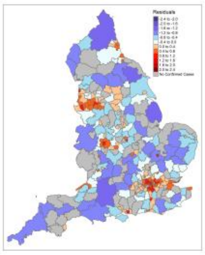	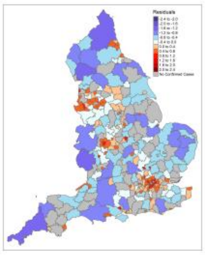	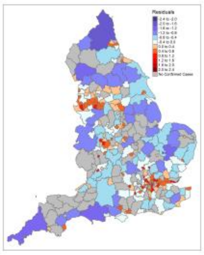
	20/04/2020	21/04/2020	22/04/2020	23/04/2020	24/04/2020

	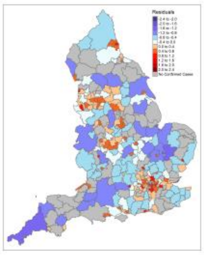	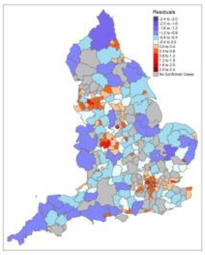	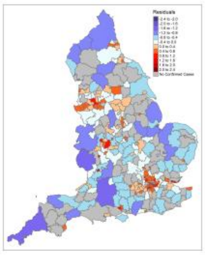	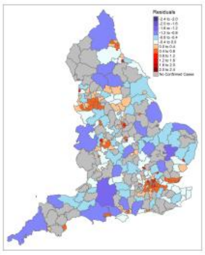	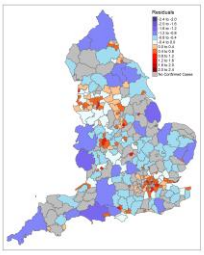
	25/04/2020	26/04/2020	27/04/2020	28/04/2020	29/04/2020
	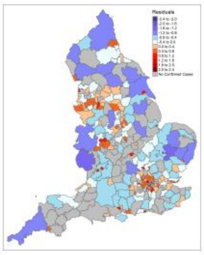	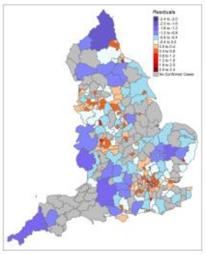	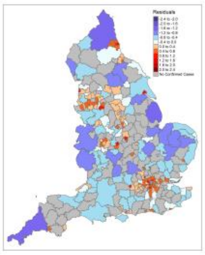	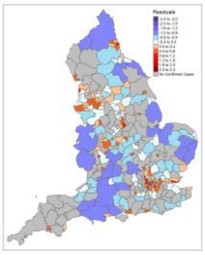	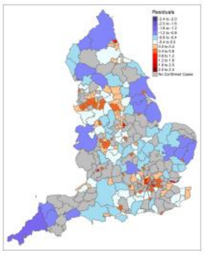
	30/04/2020	01/05/2020	02/05/2020	03/05/2020	04/05/2020
	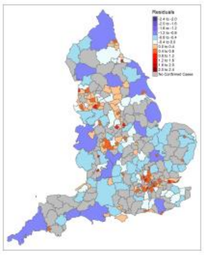	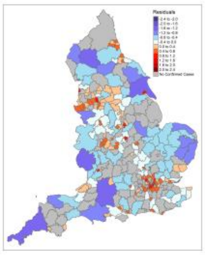	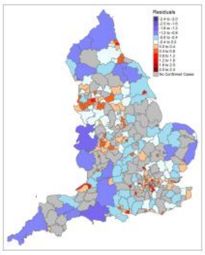	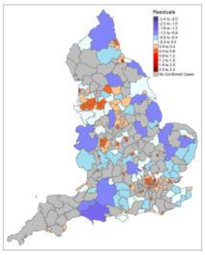	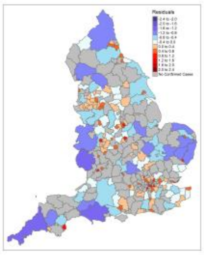
	05/05/2020	06/05/2020	07/05/2020	08/05/2020	09/05/2020
	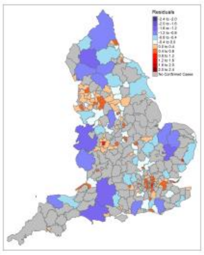	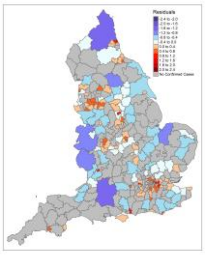	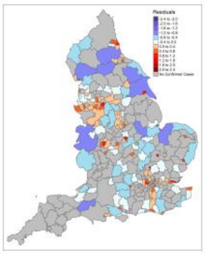	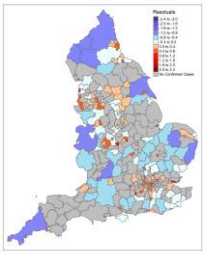	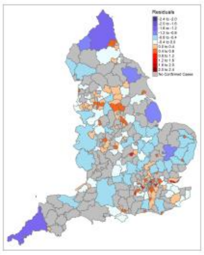
	10/05/2020	11/05/2020	12/05/2020	13/05/2020	14/05/2020
	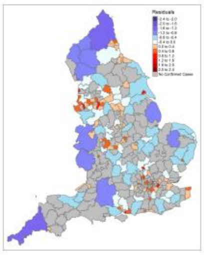	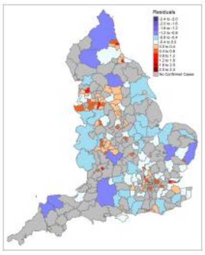	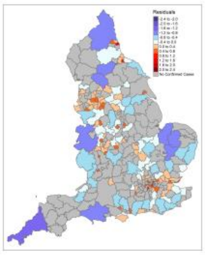	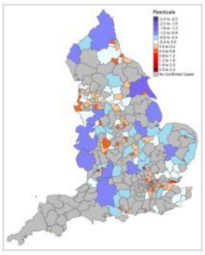	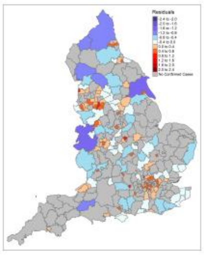
	15/05/2020	16/05/2020	17/05/2020	18/05/2020	19/05/2020

	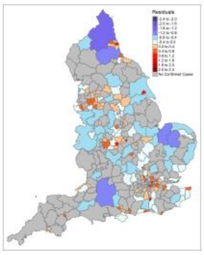	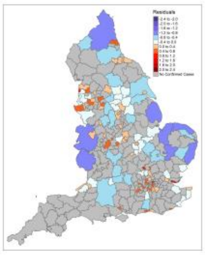	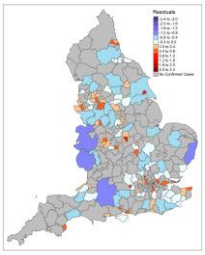	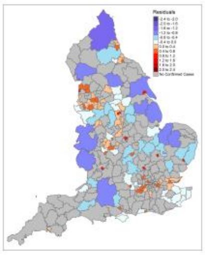	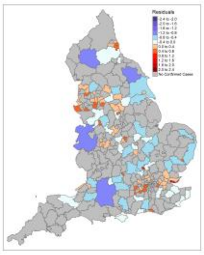
	20/05/2020	21/05/2020	22/05/2020	23/05/2020	24/05/2020
	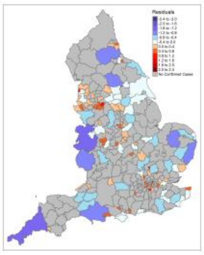	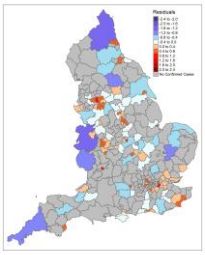	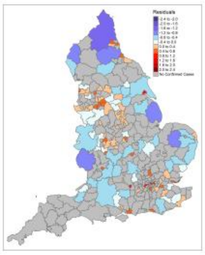	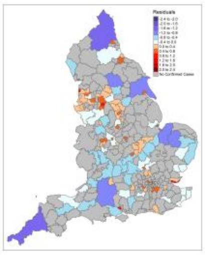	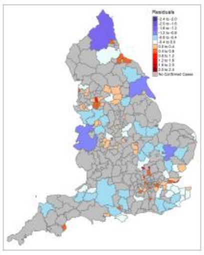
	25/05/2020	26/05/2020	27/05/2020	28/05/2020	29/05/2020
	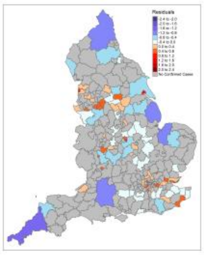	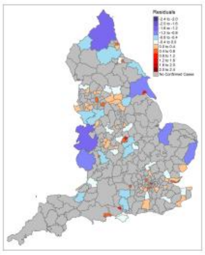	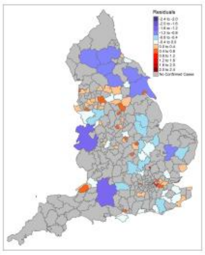	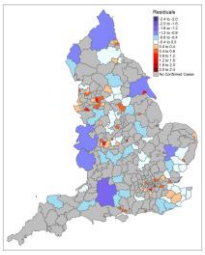	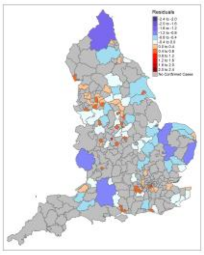
	30/05/2020	31/05/2020	01/06/2020	02/06/2020	03/06/2020
	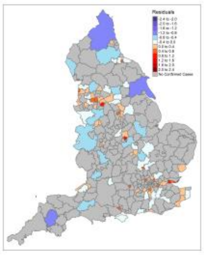	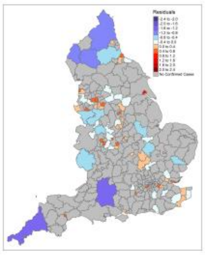	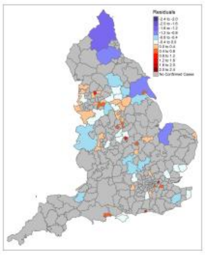	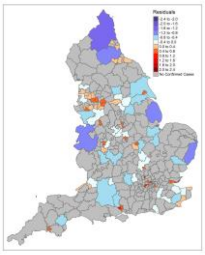	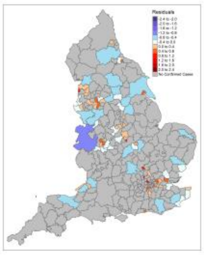
	04/06/2020	05/06/2020	06/06/2020	07/06/2020	08/06/2020
	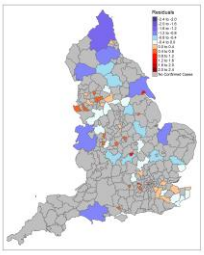	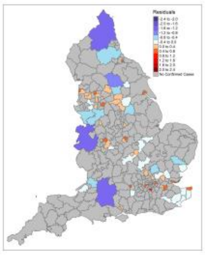	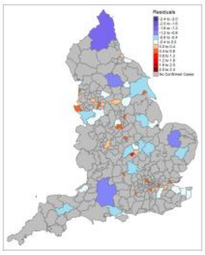	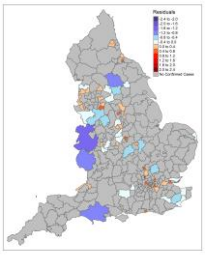	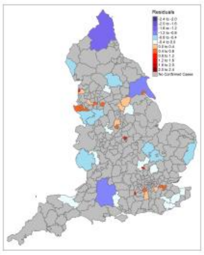
	09/06/2020	10/06/2020	11/06/2020	12/06/2020	13/06/2020

	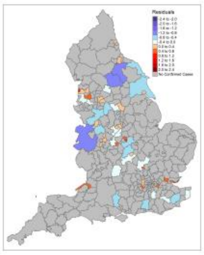	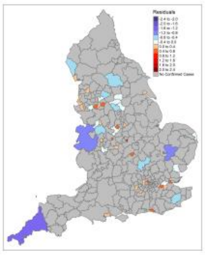	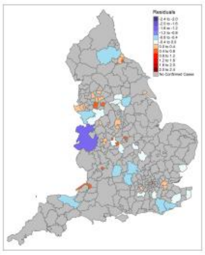	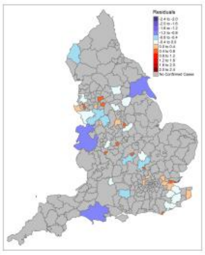	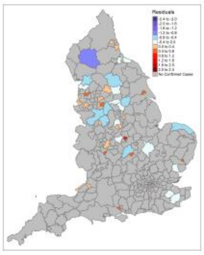
	14/06/2020	15/06/2020	16/06/2020	17/06/2020	18/06/2020
	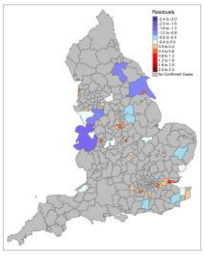	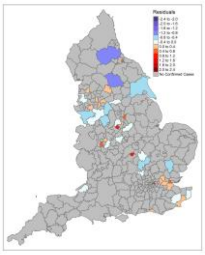	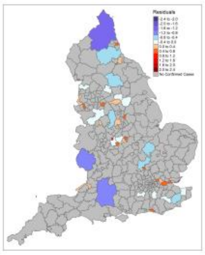	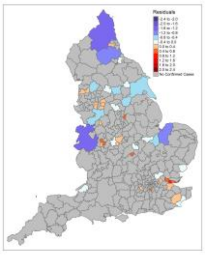	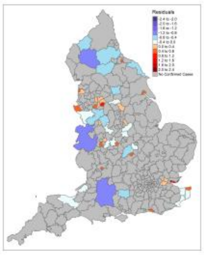
	19/06/2020	20/06/2020	21/06/2020	22/06/2020	23/06/2020
	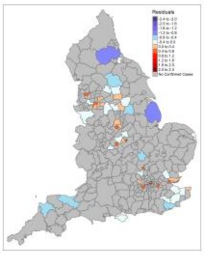	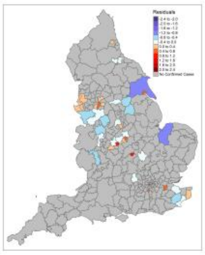	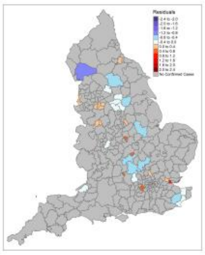	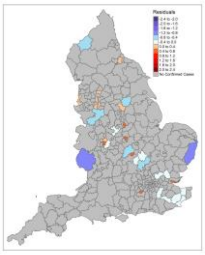	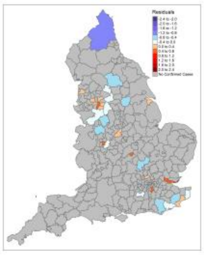
	24/06/2020	25/06/2020	26/06/2020	27/06/2020	28/06/2020
	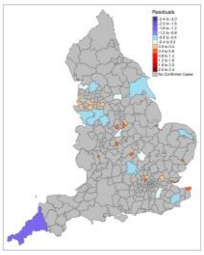	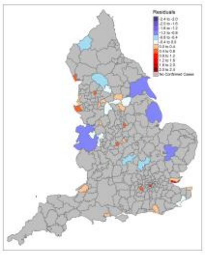	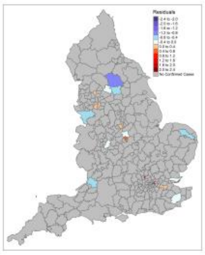	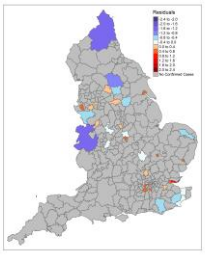	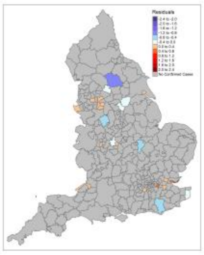
	29/06/2020	30/06/2020	01/07/2020	02/07/2020	03/07/2020
	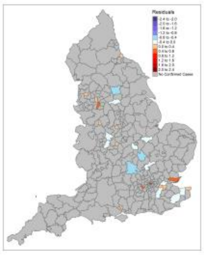	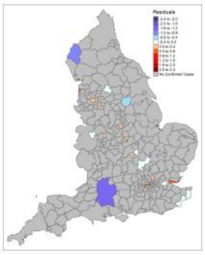	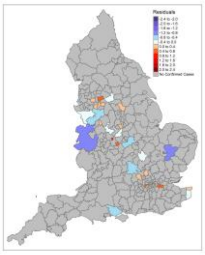	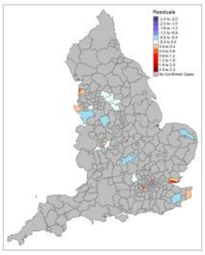	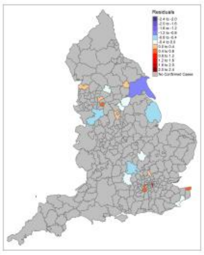
	04/07/2020	05/07/2020	06/07/2020	07/07/2020	08/07/2020

	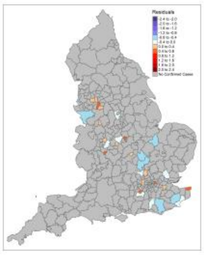	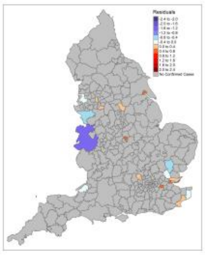	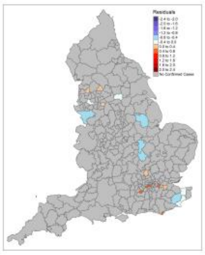	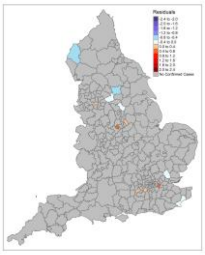	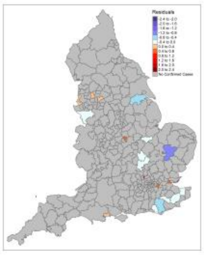
	09/07/2020	10/07/2020	11/07/2020	12/07/2020	13/07/2020
	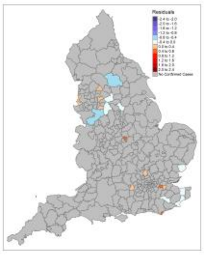	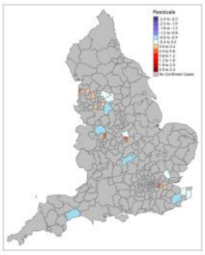	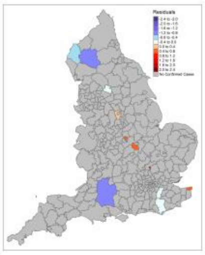	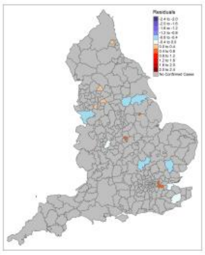	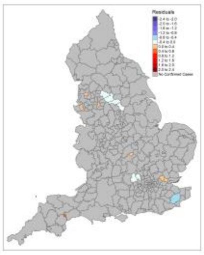
	14/07/2020	15/07/2020	16/07/2020	17/07/2020	18/07/2020
	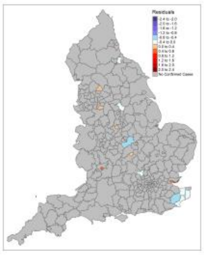	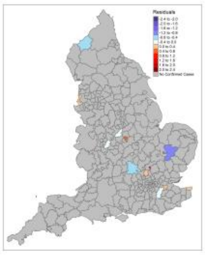	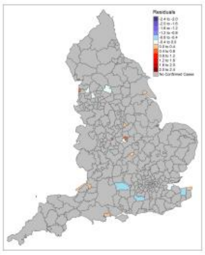	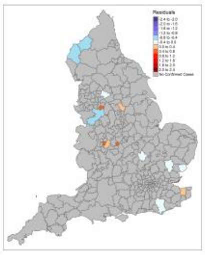	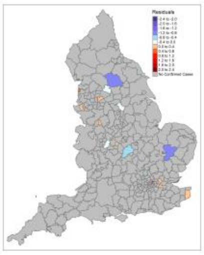
	19/07/2020	20/07/2020	21/07/2020	22/07/2020	23/07/2020
	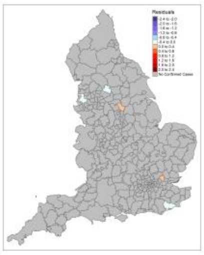	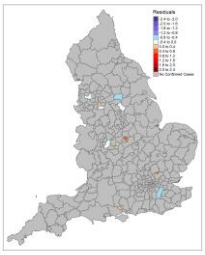	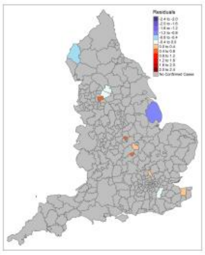	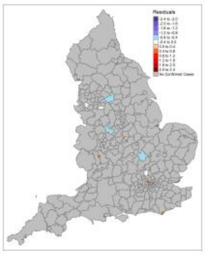	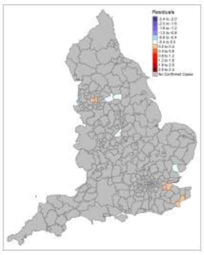
	24/07/2020	25/07/2020	26/07/2020	27/07/2020	28/07/2020
	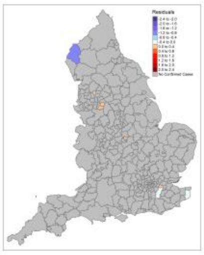	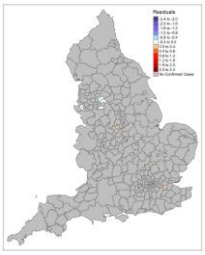	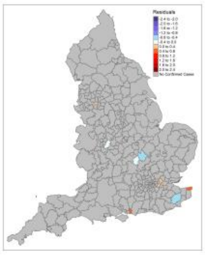	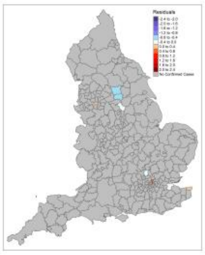	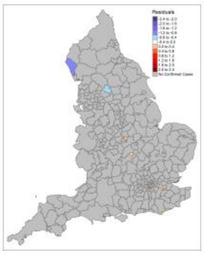
	29/07/2020	30/07/2020	31/07/2020	01/08/2020	02/08/2020

	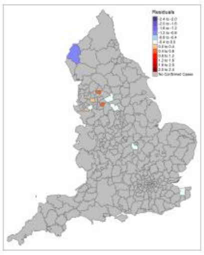	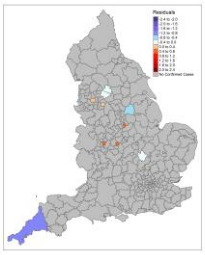	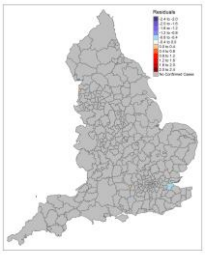	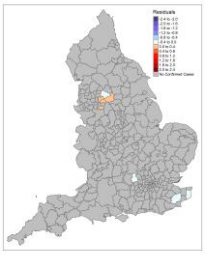	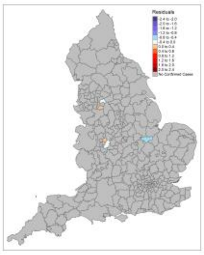
	03/08/2020	04/08/2020	05/08/2020	06/08/2020	07/08/2020
	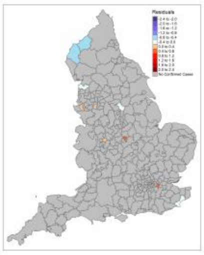	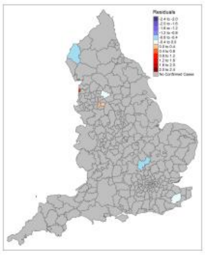	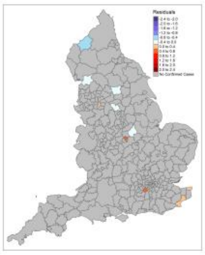	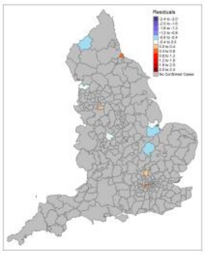	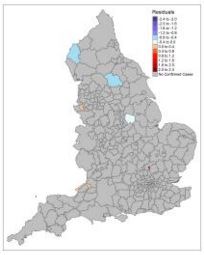
	08/08/2020	09/08/2020	10/08/2020	11/08/2020	12/08/2020
	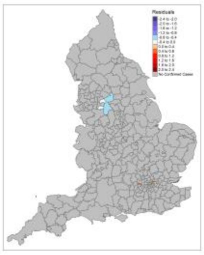	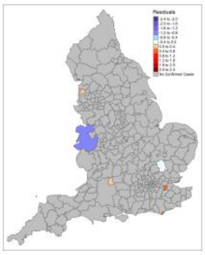	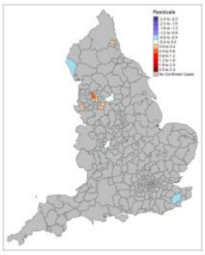	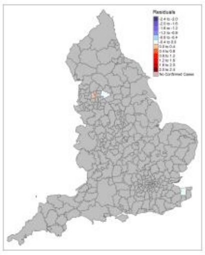	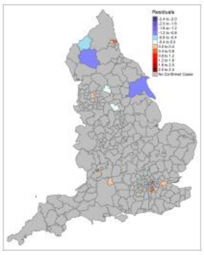
	13/08/2020	14/08/2020	15/08/2020	16/08/2020	17/08/2020
	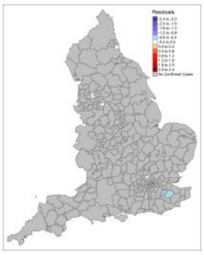		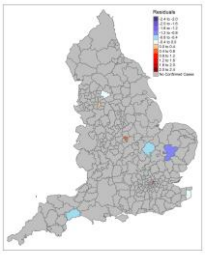	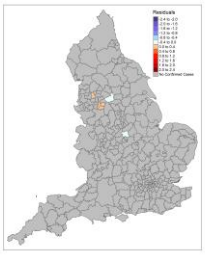	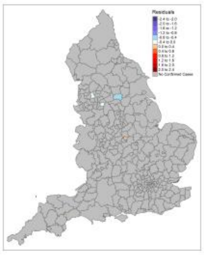
	18/08/2020	19/08/2020	20/08/2020	21/08/2020	22/08/2020
	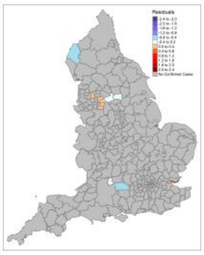	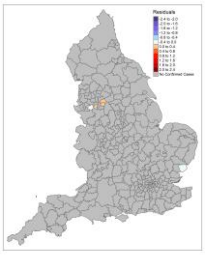	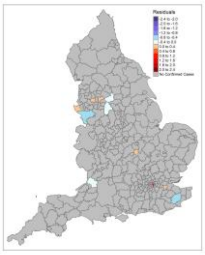	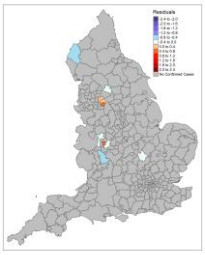	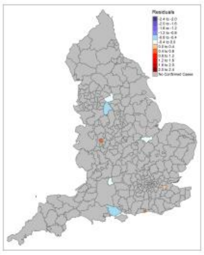
	23/08/2020	24/08/2020	25/08/2020	26/08/2020	27/08/2020

	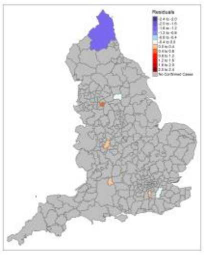	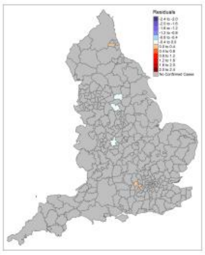	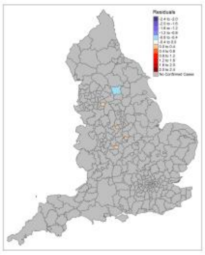	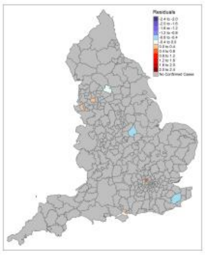	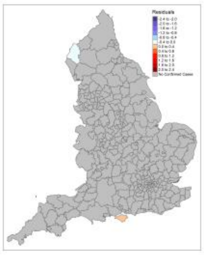
	28/08/2020	29/08/2020	30/08/2020	31/08/2020	01/09/2020
	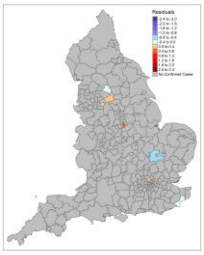	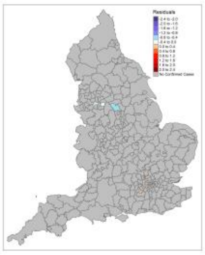	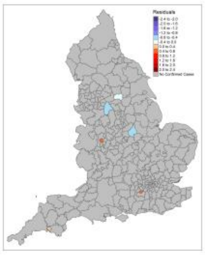	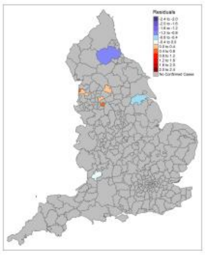	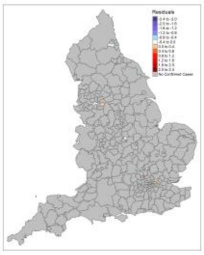
	02/09/2020	03/09/2020	04/09/2020	05/09/2020	06/09/2020
	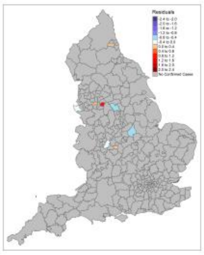	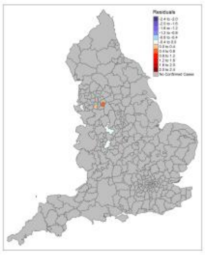	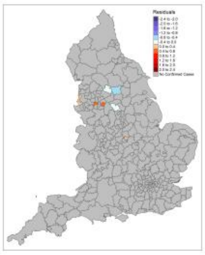	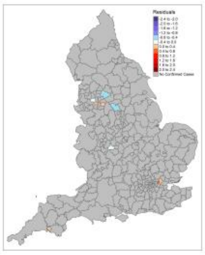	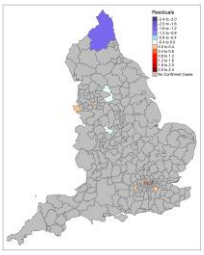
	07/09/2020	08/09/2020	09/09/2020	10/09/2020	11/09/2020
	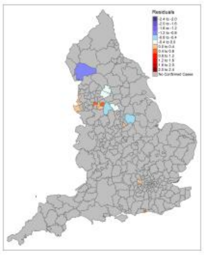	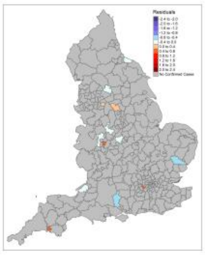	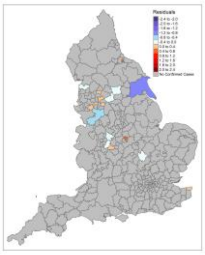	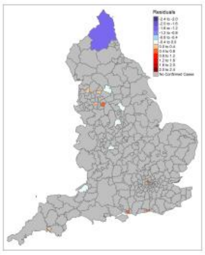	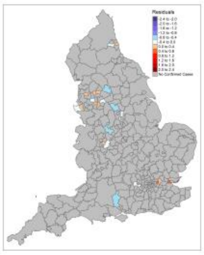
	12/09/2020	13/09/2020	14/09/2020	15/09/2020	16/09/2020
	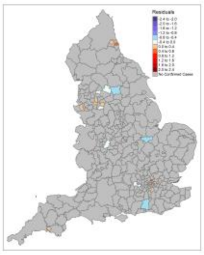	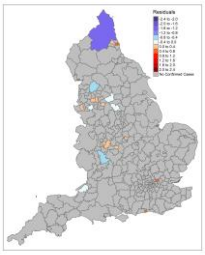	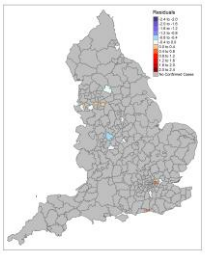	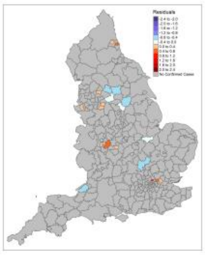	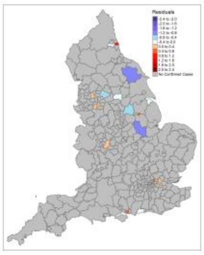
	17/09/2020	18/09/2020	19/09/2020	20/09/2020	21/09/2020

	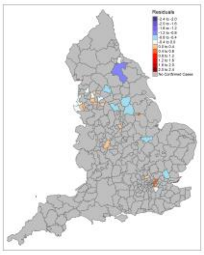	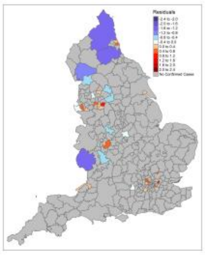	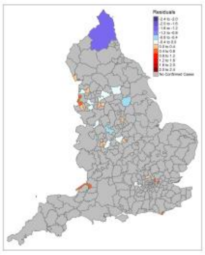	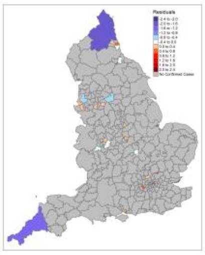	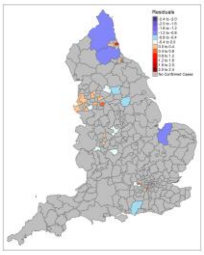
	22/09/2020	23/09/2020	24/09/2020	25/09/2020	26/09/2020
	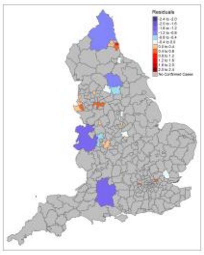	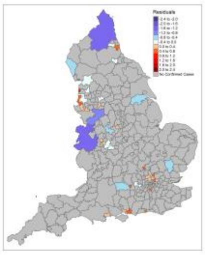	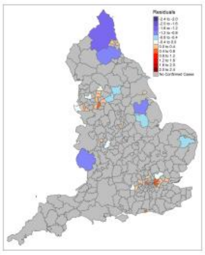	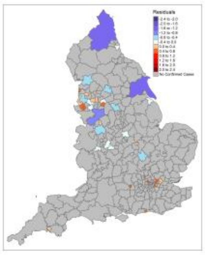	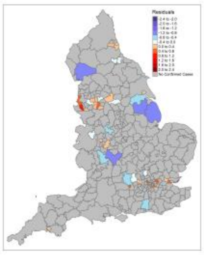
	27/09/2020	28/09/2020	29/09/2020	30/09/2020	01/10/2020
	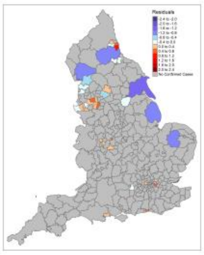	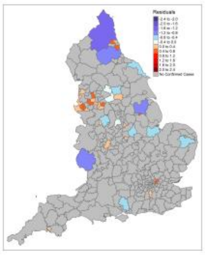	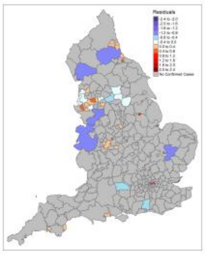	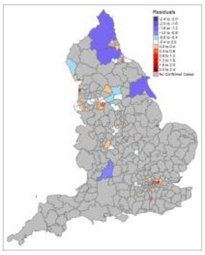	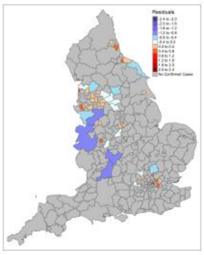
	02/10/2020	03/10/2020	04/10/2020	05/10/2020	06/10/2020
	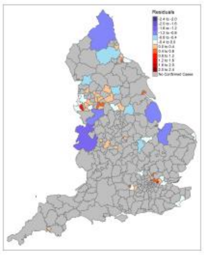	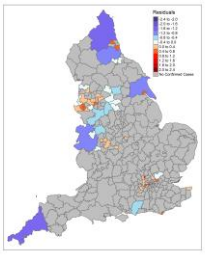	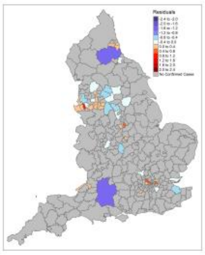	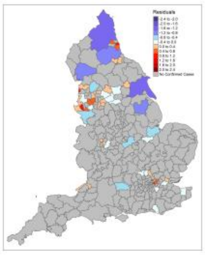	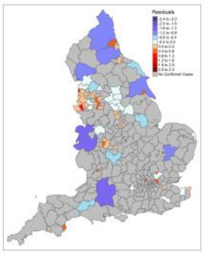
	07/10/2020	08/10/2020	09/10/2020	10/10/2020	11/10/2020
	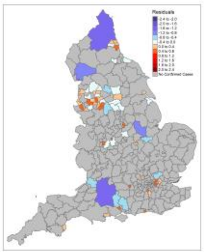	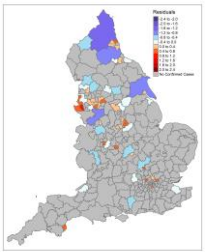	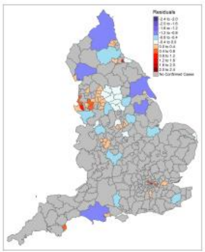	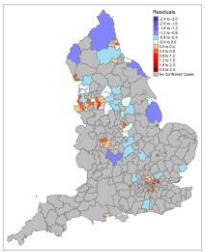	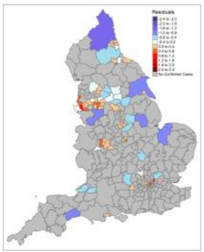
	12/10/2020	13/10/2020	14/10/2020	15/10/2020	16/10/2020

	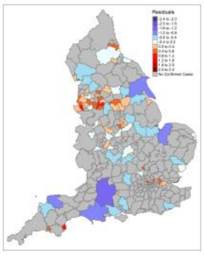	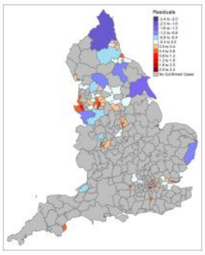	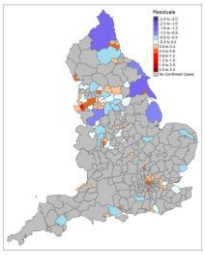	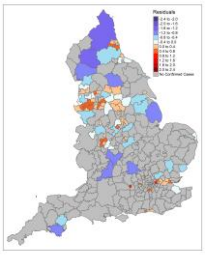	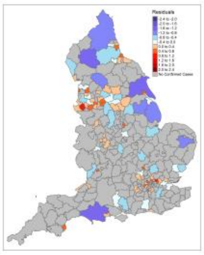
	17/10/2020	18/10/2020	19/10/2020	20/10/2020	21/10/2020
	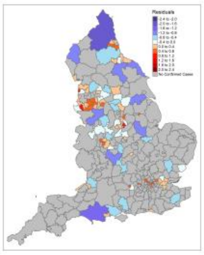	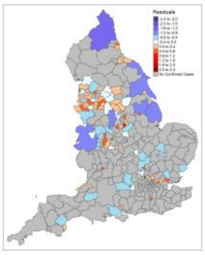	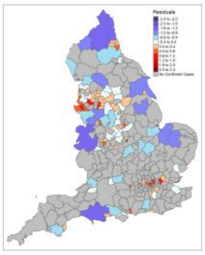	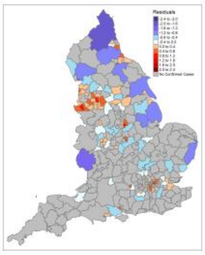	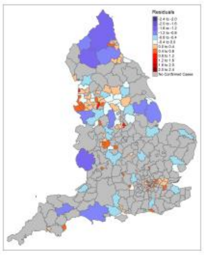
	22/10/2020	23/10/2020	24/10/2020	25/10/2020	26/10/2020
	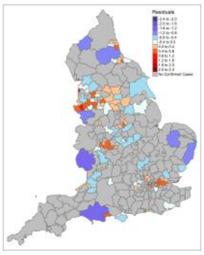	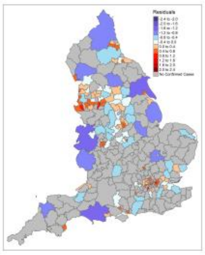	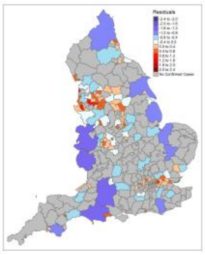	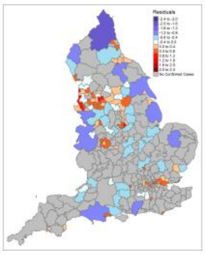	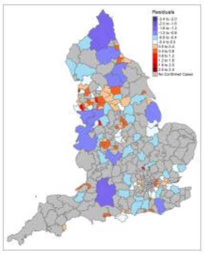
	27/10/2020	28/10/2020	29/10/2020	30/10/2020	31/10/2020
	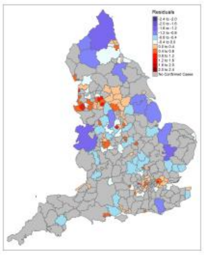	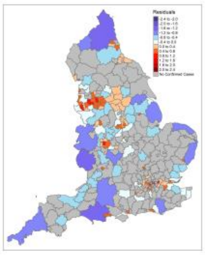	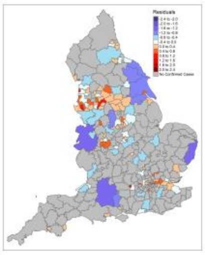	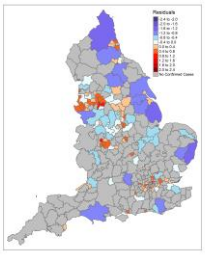	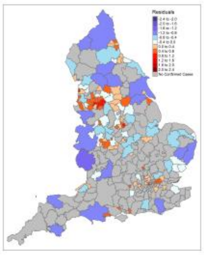
	01/11/2020	02/11/2020	03/11/2020	04/11/2020	05/11/2020
	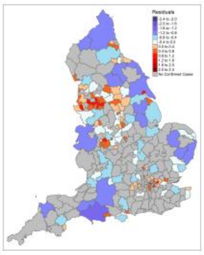	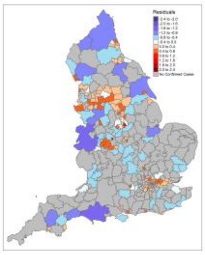	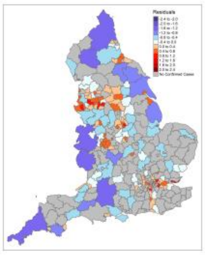	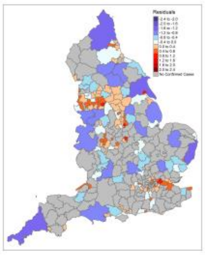	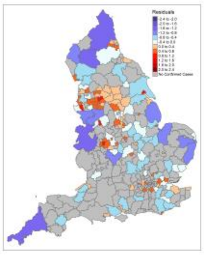
	06/11/2020	07/11/2020	08/11/2020	09/11/2020	10/11/2020

	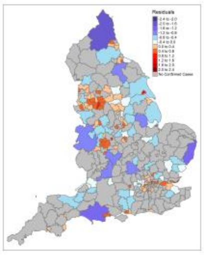	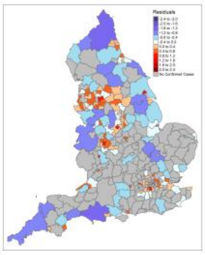	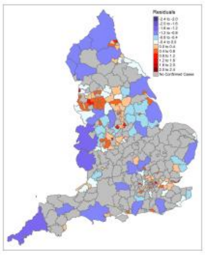	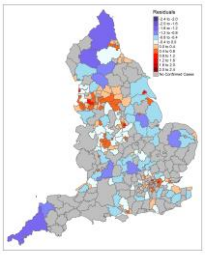	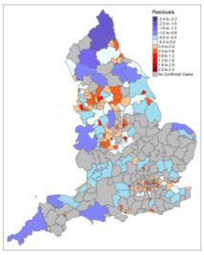
	11/11/2020	12/11/2020	13/11/2020	14/11/2020	15/11/2020
	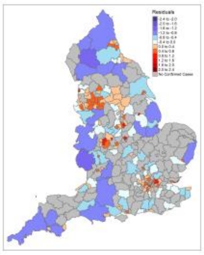	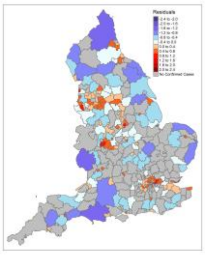	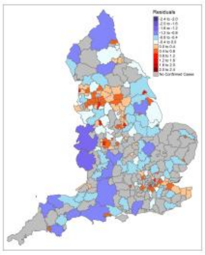	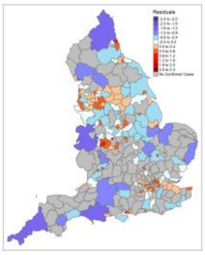	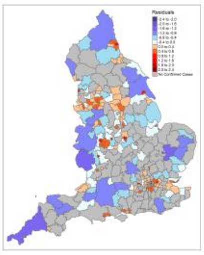
	16/11/2020	17/11/2020	18/11/2020	19/11/2020	20/11/2020
	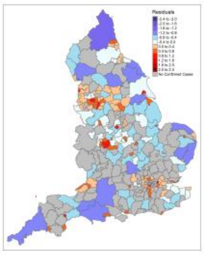	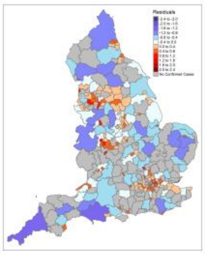	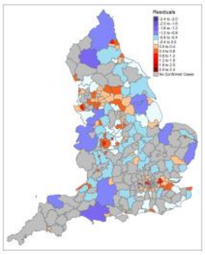	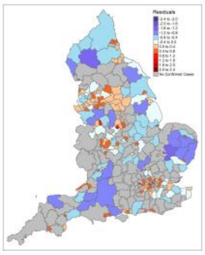	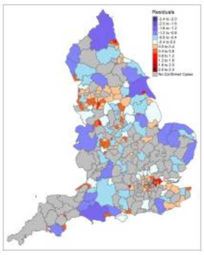
	21/11/2020	22/11/2020	23/11/2020	24/11/2020	25/11/2020
	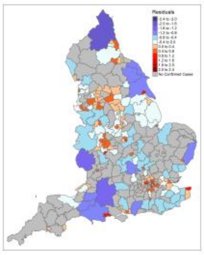	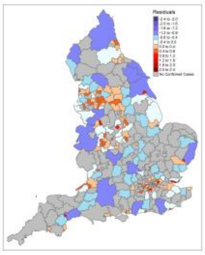	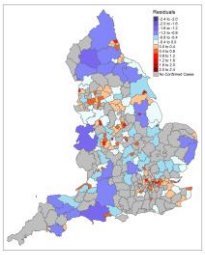	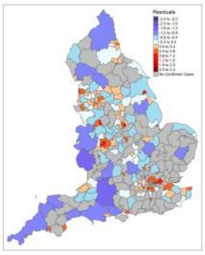	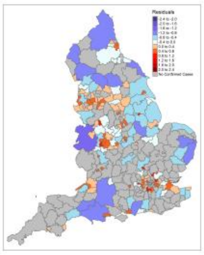
	26/11/2020	27/11/2020	28/11/2020	29/11/2020	30/11/2020
	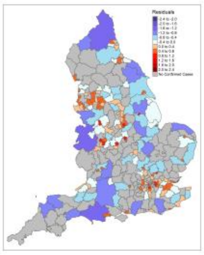	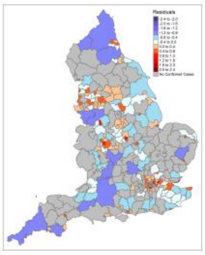	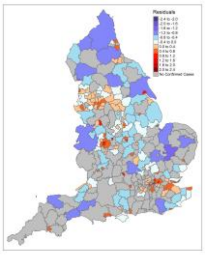	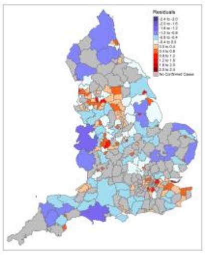	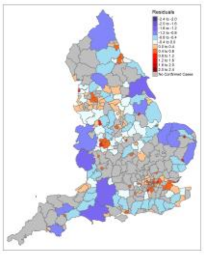
	01/12/2020	02/12/2020	03/12/2020	04/12/2020	05/12/2020

	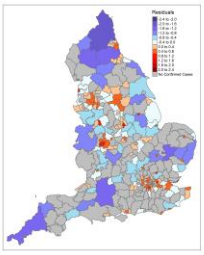	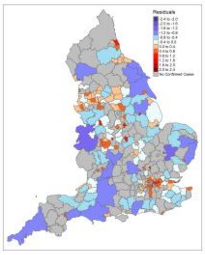	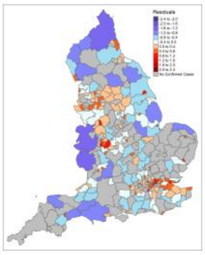	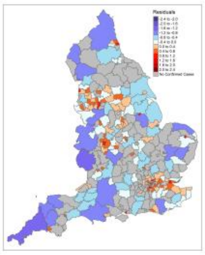	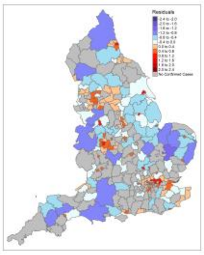
	06/12/2020	07/12/2020	08/12/2020	09/12/2020	10/12/2020
	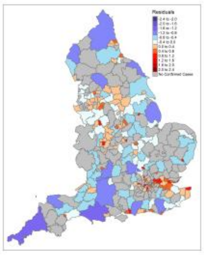	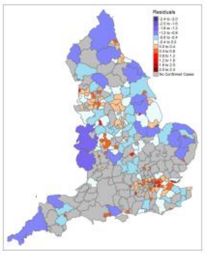	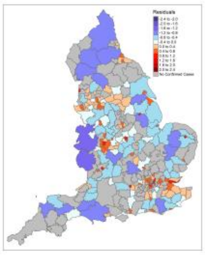	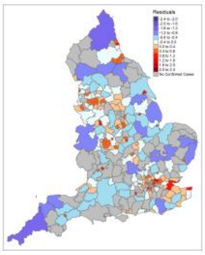	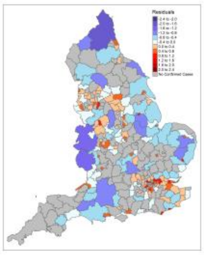
	11/12/2020	12/12/2020	13/12/2020	14/12/2020	15/12/2020
	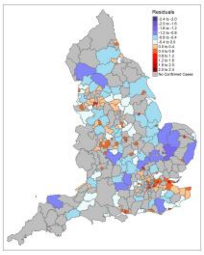	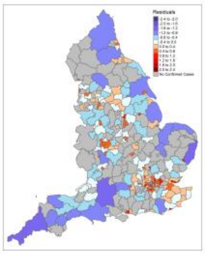	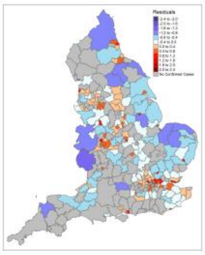	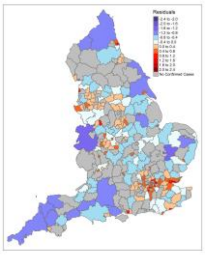	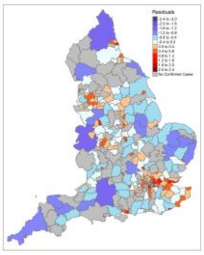
	16/12/2020	17/12/2020	18/12/2020	19/12/2020	20/12/2020
	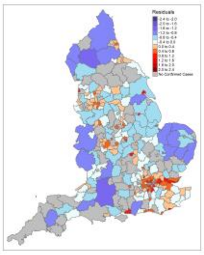	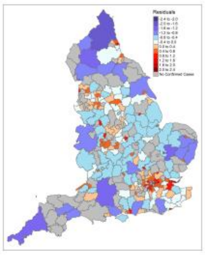	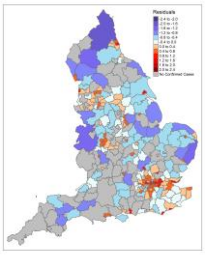	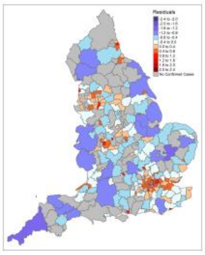	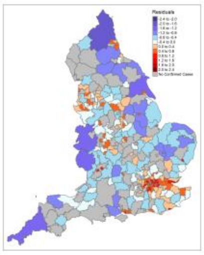
	21/12/2020	22/12/2020	23/12/2020	24/12/2020	25/12/2020
	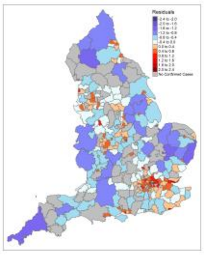	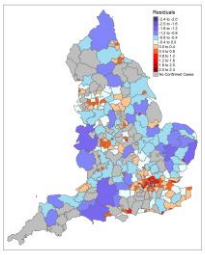	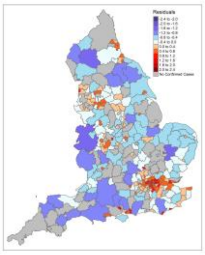	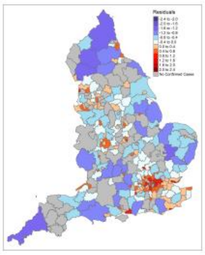	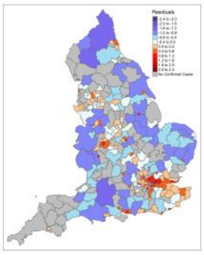
	26/12/2020	27/12/2020	28/12/2020	29/12/2020	30/12/2020

	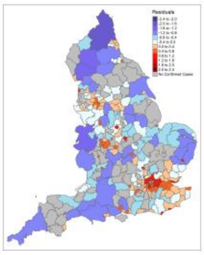	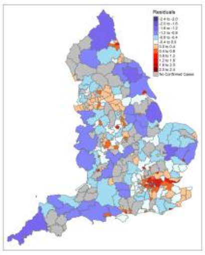	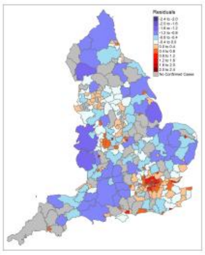	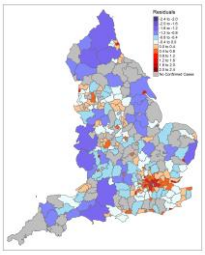	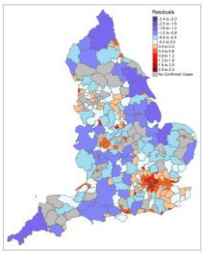
	31/12/2020	01/01/2021	02/01/2021	03/01/2021	04/01/2021
	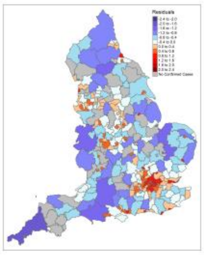	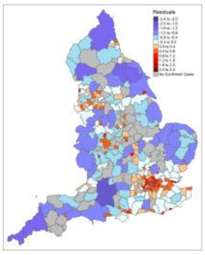	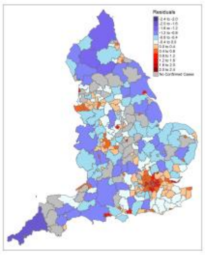	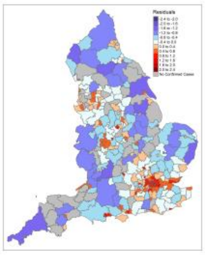	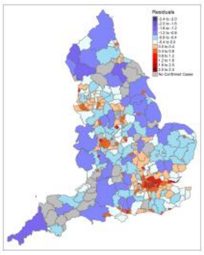
	05/01/2021	06/01/2021	07/01/2021	08/01/2021	09/01/2021
	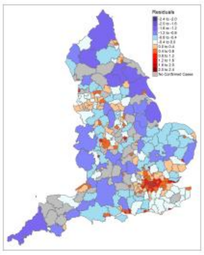	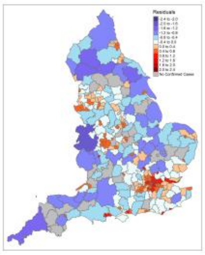	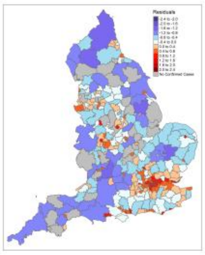	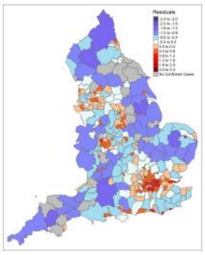	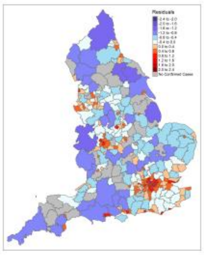
	10/01/2021	11/01/2021	12/01/2021	13/01/2021	14/01/2021
	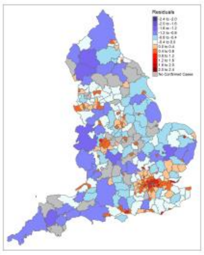	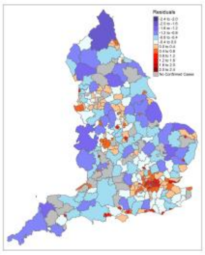	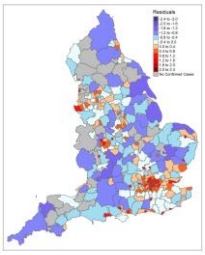	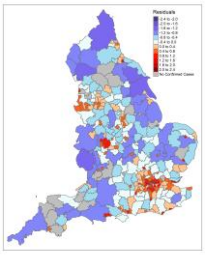	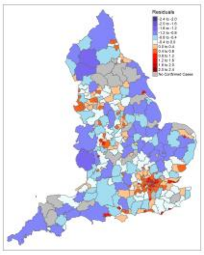
	15/01/2021	16/01/2021	17/01/2021	18/01/2021	19/01/2021
	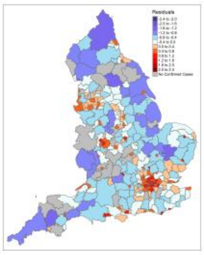	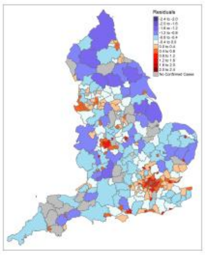	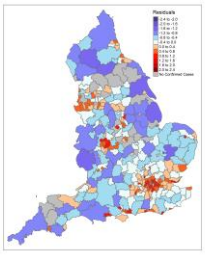	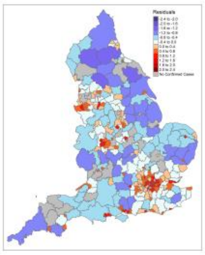	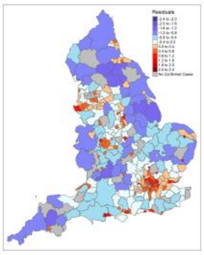
	20/01/2021	21/01/2021	22/01/2021	23/01/2021	24/01/2021

	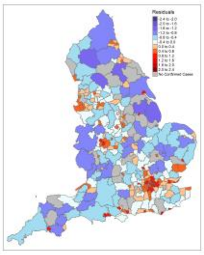	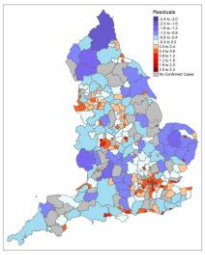	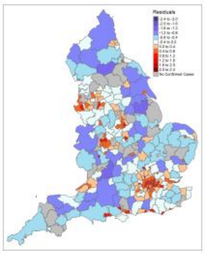	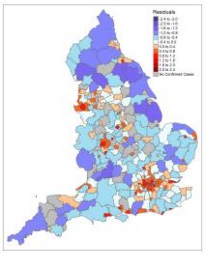	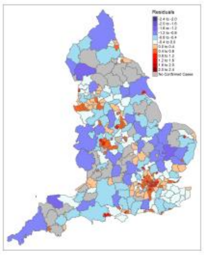
	25/01/2021	26/01/2021	27/01/2021	28/01/2021	29/01/2021
	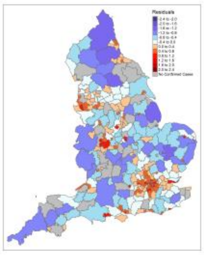	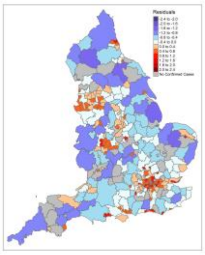	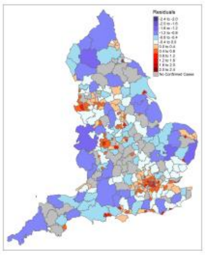	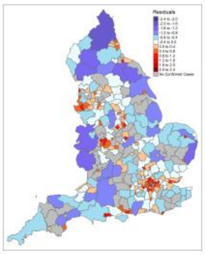	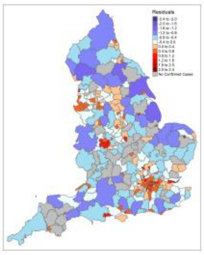
	30/01/2021	31/01/2021	01/02/2021	02/02/2021	03/02/2021
	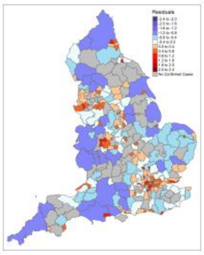	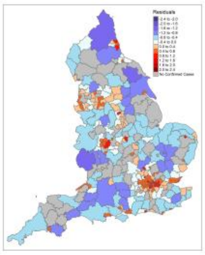	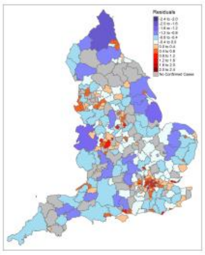	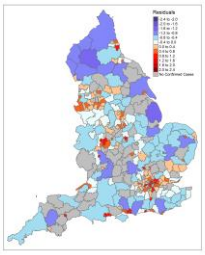	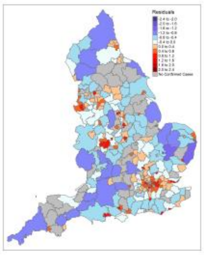
	04/02/2021	05/02/2021	06/02/2021	07/02/2021	08/02/2021
	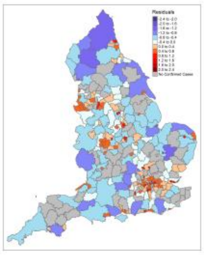	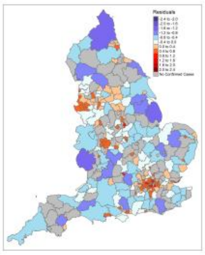	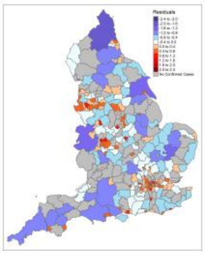	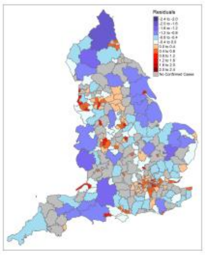	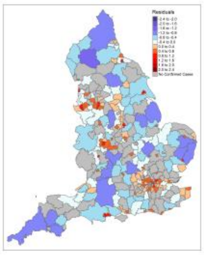
	09/02/2021	10/02/2021	11/02/2021	12/02/2021	13/02/2021
	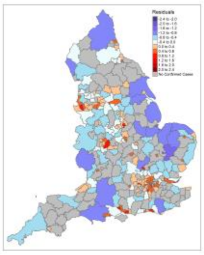	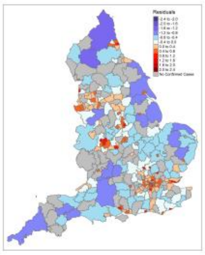	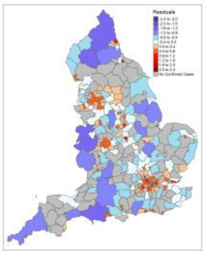	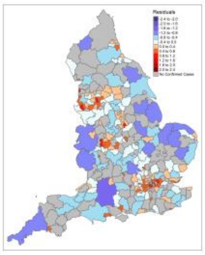	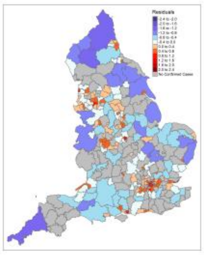
	14/02/2021	15/02/2021	16/02/2021	17/02/2021	18/02/2021

	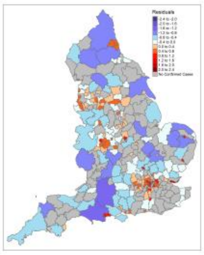	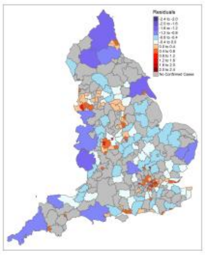	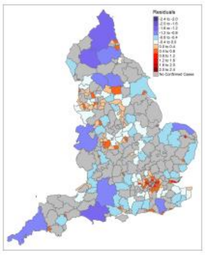	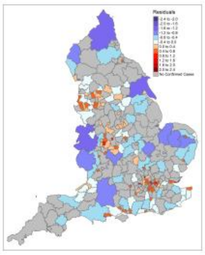	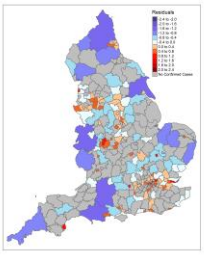
	19/02/2021	20/02/2021	21/02/2021	22/02/2021	23/02/2021
	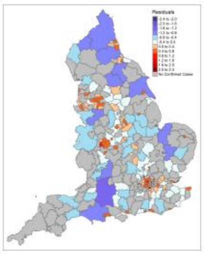	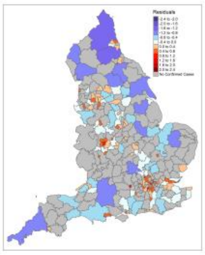	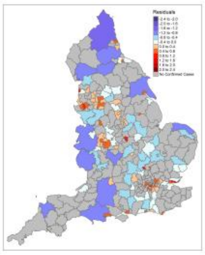	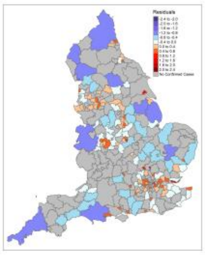	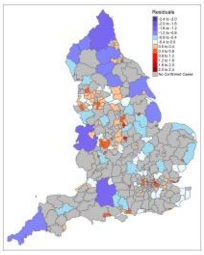
	24/02/2021	25/02/2021	26/02/2021	27/02/2021	28/02/2021
	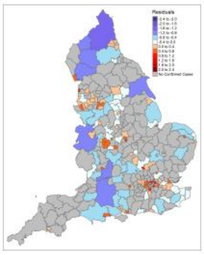	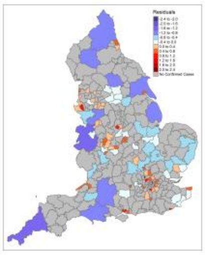	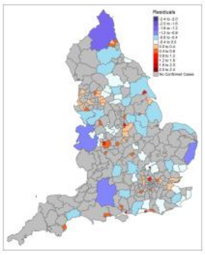	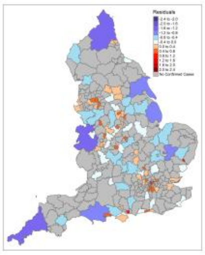	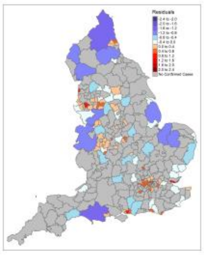
	01/03/2021	02/03/2021	03/03/2021	04/03/2021	05/03/2021
	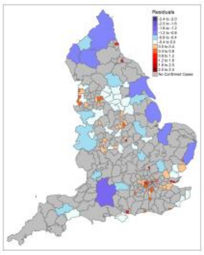	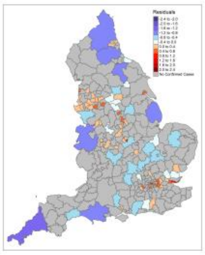	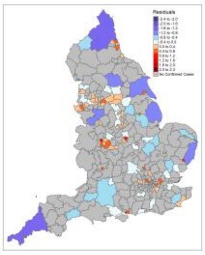	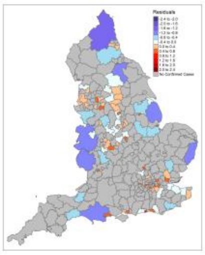	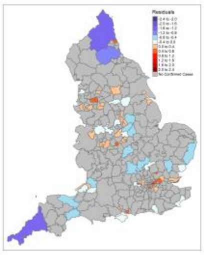
	06/03/2021	07/03/2021	08/03/2021	09/03/2021	10/03/2021
	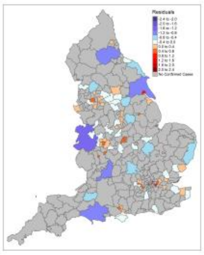	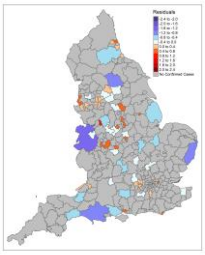	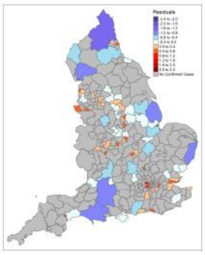	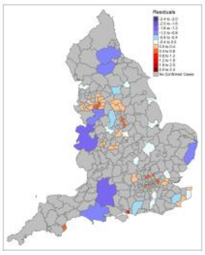	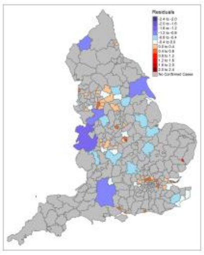
	11/03/2021	12/03/2021	13/03/2021	14/03/2021	15/03/2021

	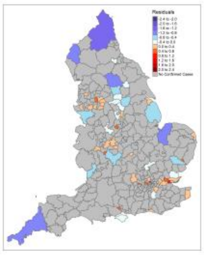	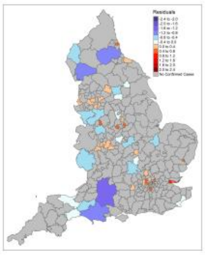	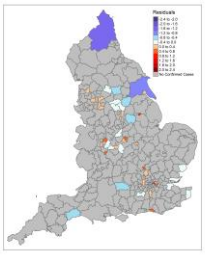	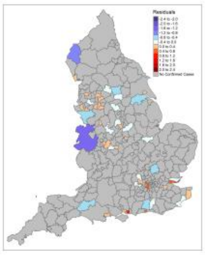	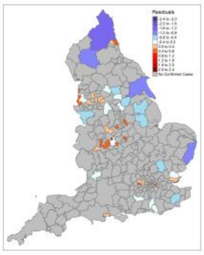
	16/03/2021	17/03/2021	18/03/2021	19/03/2021	20/03/2021
	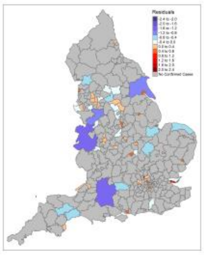	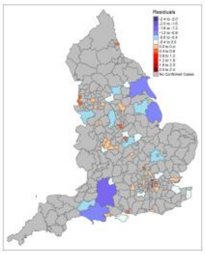	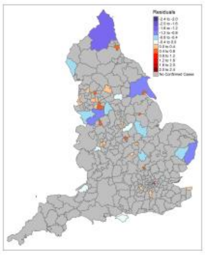	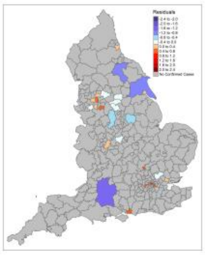	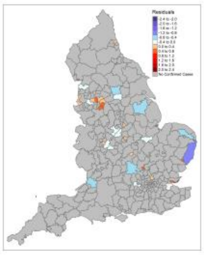
	21/03/2021	22/03/2021	23/03/2021	24/03/2021	25/03/2021
	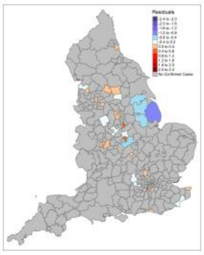	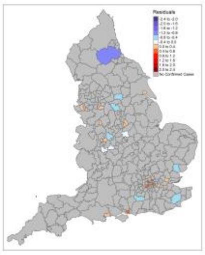	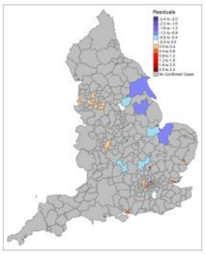	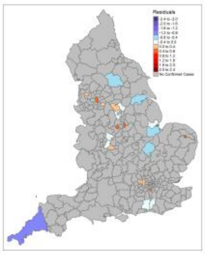	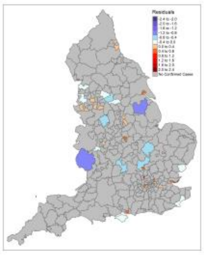
	26/03/2021	27/03/2021	28/03/2021	29/03/2021	30/03/2021
	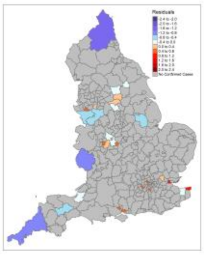	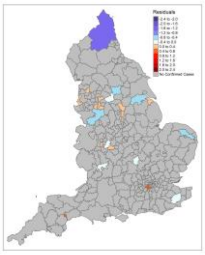	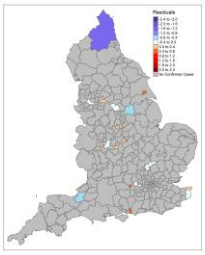	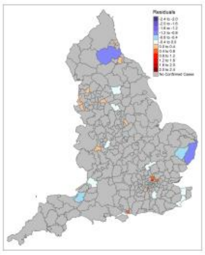	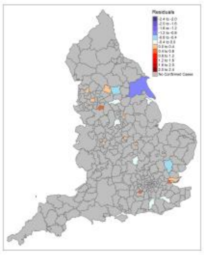
	31/03/2021	01/04/2021	02/04/2021	03/04/2021	04/04/2021
	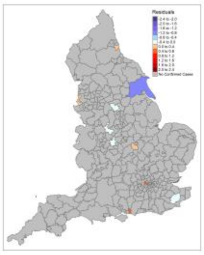	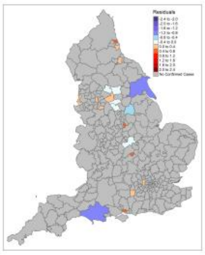	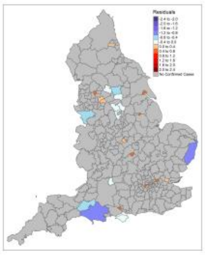	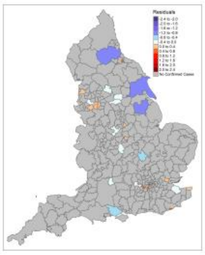	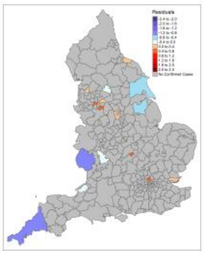
	05/04/2021	06/04/2021	07/04/2021	08/04/2021	09/04/2021

	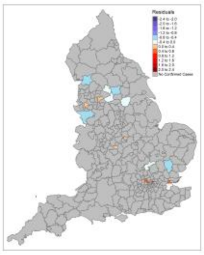	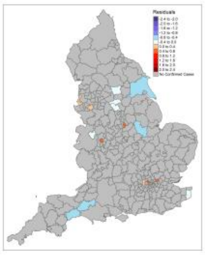	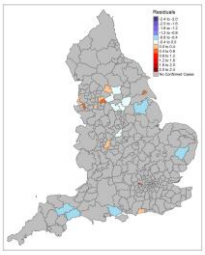	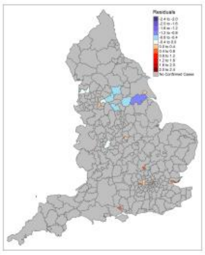	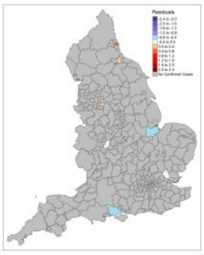
	10/04/2021	11/04/2021	12/04/2021	13/04/2021	14/04/2021
	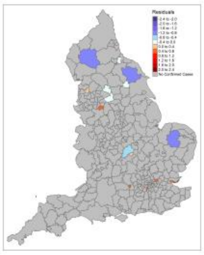	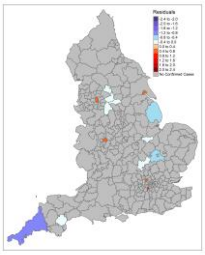	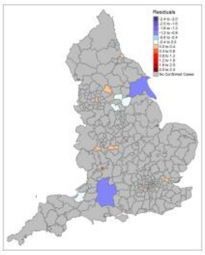	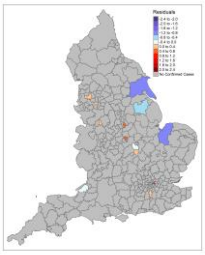	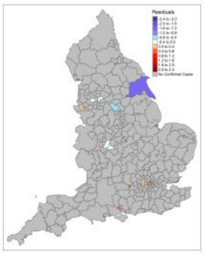
	15/04/2021	16/04/2021	17/04/2021	18/04/2021	19/04/2021
	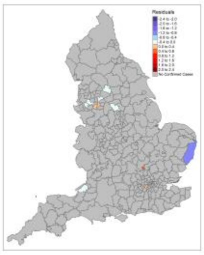	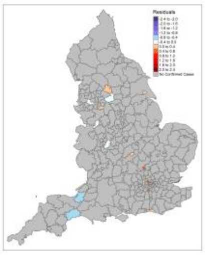	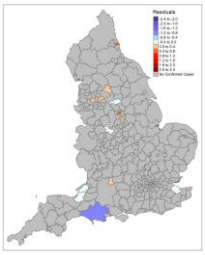	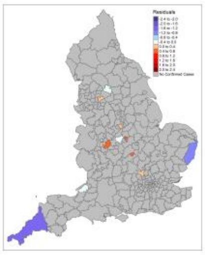	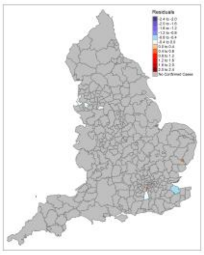
	20/04/2021	21/04/2021	22/04/2021	23/04/2021	24/04/2021
	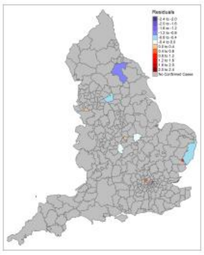	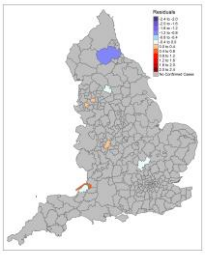	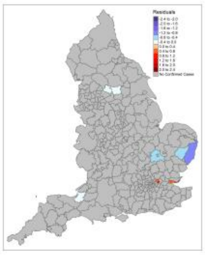	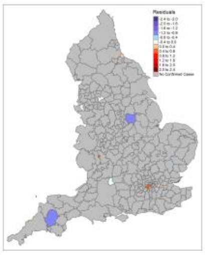	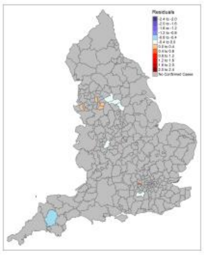
	25/04/2021	26/04/2021	27/04/2021	28/04/2021	29/04/2021
	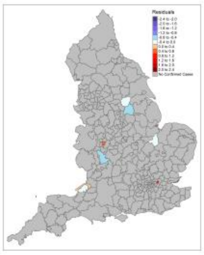	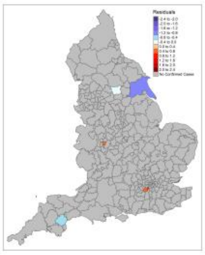	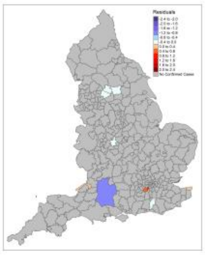	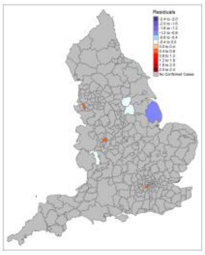	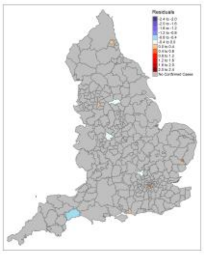
	30/04/2021	01/05/2021	02/05/2021	03/05/2021	04/05/2021

	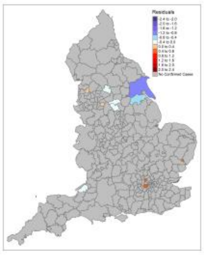	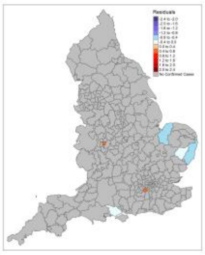	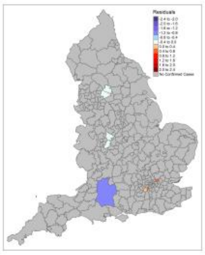	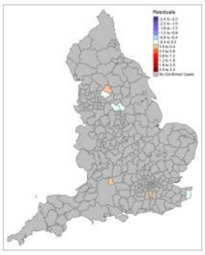	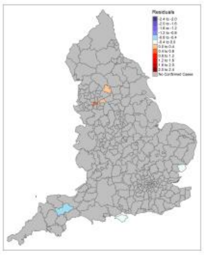
	05/05/2021	06/05/2021	07/05/2021	08/05/2021	09/05/2021
	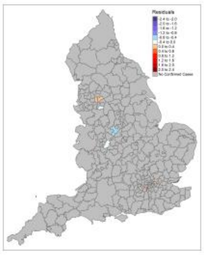	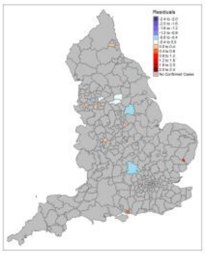	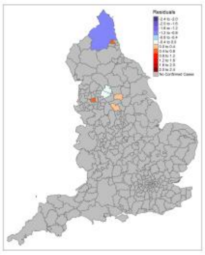	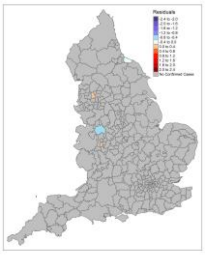	
	10/05/2021	11/05/2021	12/05/2021	13/05/2021	14/05/2021
	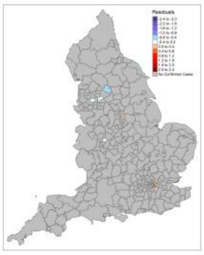	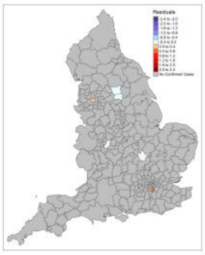	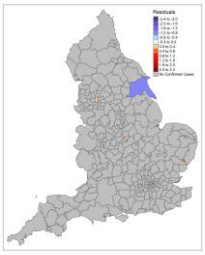	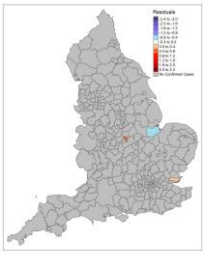	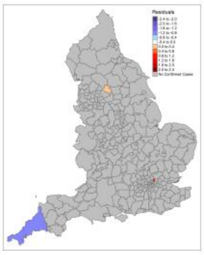
	15/05/2021	16/05/2021	17/05/2021	18/05/2021	19/05/2021
	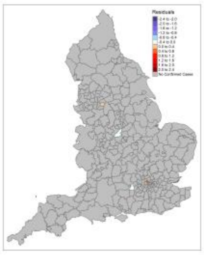				
	20/05/2021				

Fig S5. Daily geoplots of LTLA COVID-19 death residuals. Regions that are red are above expectation and blue is below. The darker the shade the further from the scaling law. The geoplots contain public sector information licensed under the Open Government Licence v3.0.
