# Supplementary material for: Population density and spreading of COVID-19 in England and Wales
Source: PLoS One. 2022 Mar 31;17(3):e0261725. doi: 10.1371/journal.pone.0261725 (PMC8970409; doi:10.1371/journal.pone.0261725)
Supplement: S6 Fig — Rank correlation of COVID-19 case residuals between all pairs of dates between 01/03/2020 and 20/05/2021. Red and blue colours refer to correlation values close to 1 and -1 respectively. The darker shade of colour is associated to a higher similarity between the two pairs. (PDF) [file pone.0261725.s006.pdf]

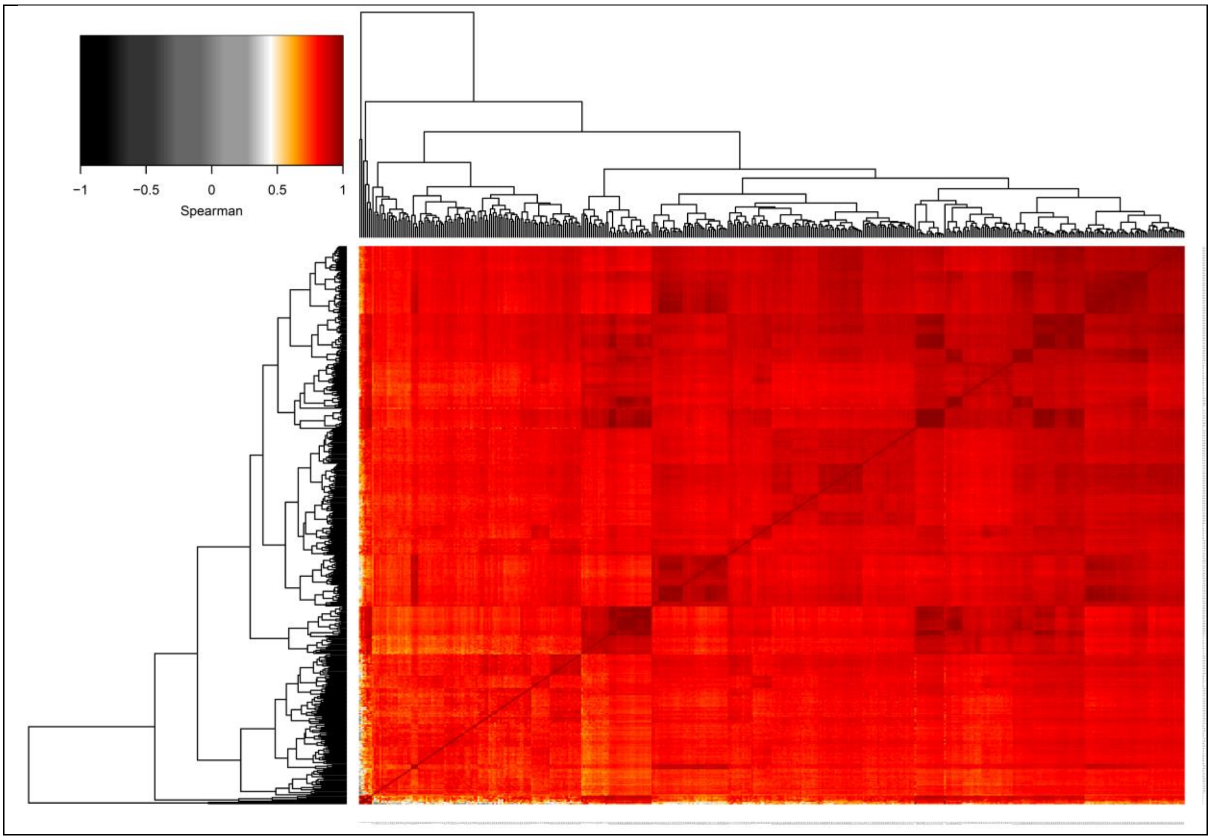

Fig S6. Spearman's rank correlation coefficient. Rank correlation of COVID-19 case residuals between all pairs of dates between 01/03/2020 and 20/05/2021. Shades of red indicate strong positive correlation while white and grayscale indicates low and negative correlation. The darker shade of colour is associated to a higher similarity between the two pairs.
