# Supplementary material for: Population density and spreading of COVID-19 in England and Wales
Source: PLoS One. 2022 Mar 31;17(3):e0261725. doi: 10.1371/journal.pone.0261725 (PMC8970409; doi:10.1371/journal.pone.0261725)
Supplement: S7 Fig — Single power law models are represented with one single red line. Double power law models are represented with a red line, below the critical density and a green line above the critical density. The black circle is the position of the critical density. (PDF) [file pone.0261725.s007.pdf]

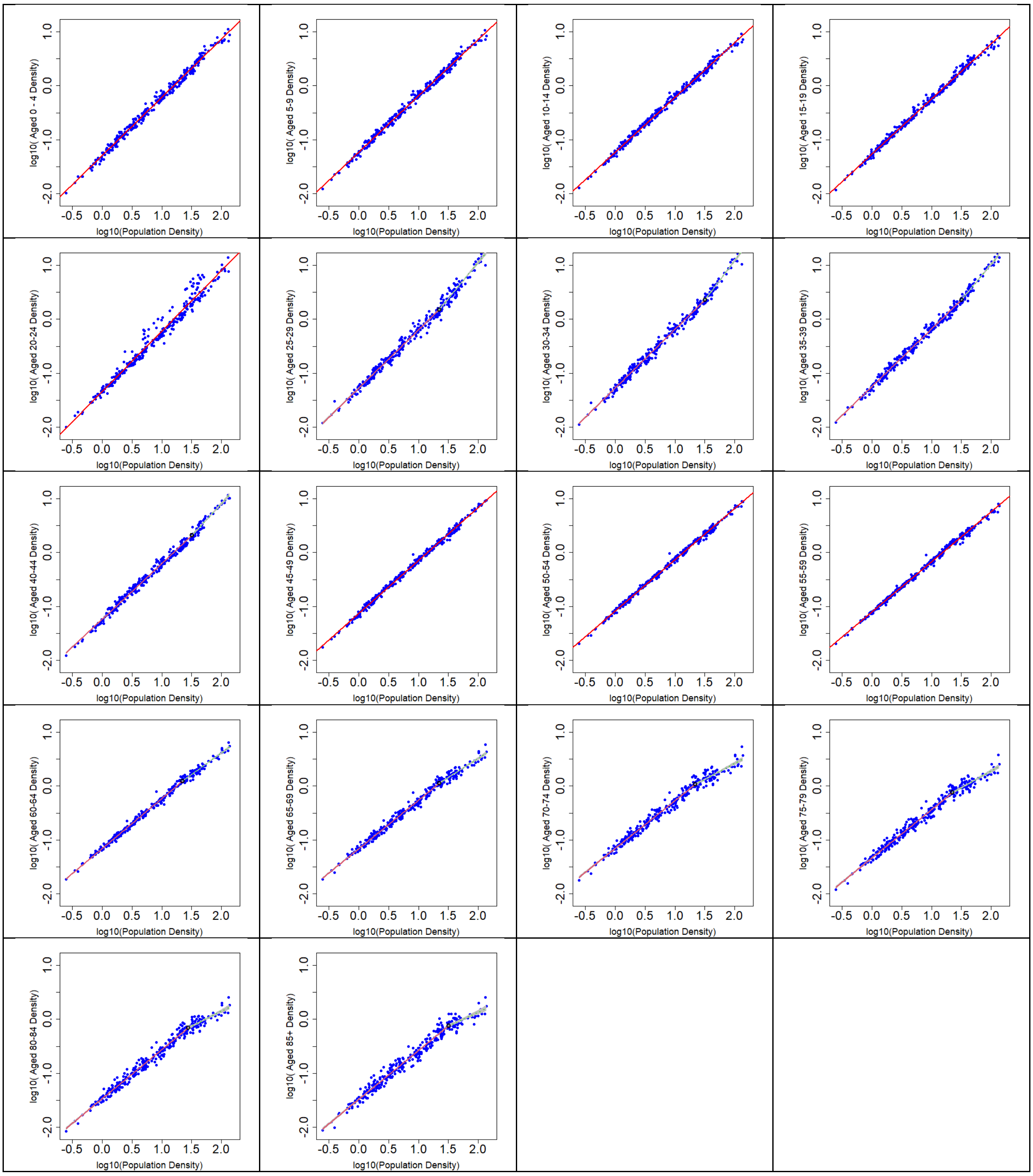

Fig S7. Single and double power law scaling models identified using a Davies test. Single power law models are represented with one single red line. Double power law models are represented with a red line, below the critical density and a green line above the critical density. The black circle is the position of the critical density.
