# Supplementary material for: Population density and spreading of COVID-19 in England and Wales
Source: PLoS One. 2022 Mar 31;17(3):e0261725. doi: 10.1371/journal.pone.0261725 (PMC8970409; doi:10.1371/journal.pone.0261725)
Supplement: S8 Fig — The lower triangle are residual scatter plots, the diagonal are density histograms and the upper triangle are the Spearman’s rank correlation coefficients. (PDF) [file pone.0261725.s008.pdf]

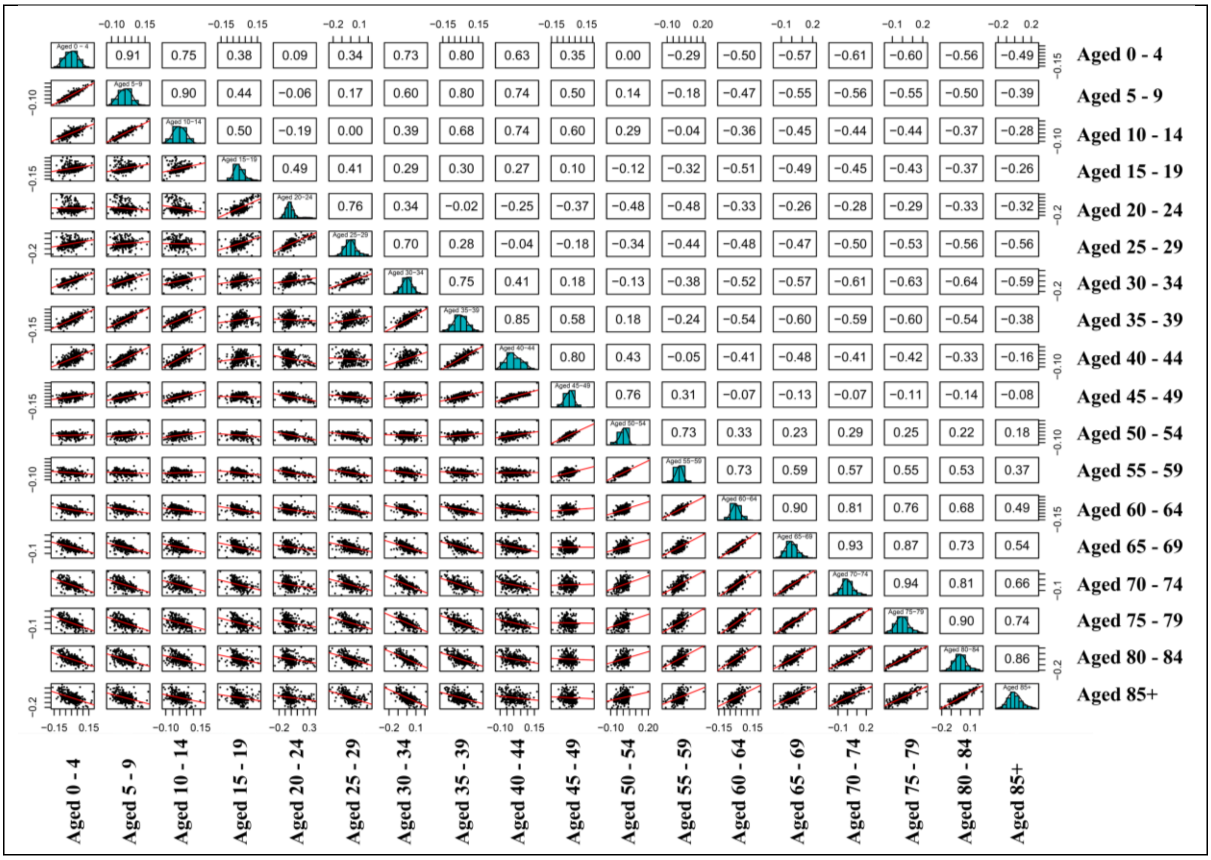

Fig S8. Residual relationships between all 18 age categories. The lower triangle are residual scatter plots, the diagonal are density histograms and the upper triangle are the Spearman's rank correlation coefficients.
